# Stereoselective Synthesis of Cannabidiol and its Unnatural Enantiomer to Evaluate Anxiolytic‐Like Properties and Memory Interactions

**DOI:** 10.1002/cbic.202500438

**Published:** 2025-07-24

**Authors:** Marcos Accioly Jr, Kawana Rosa, Andressa G. Soliani, Suzete M. Cerutti, Cristiano Raminelli

**Affiliations:** ^1^ Department of Chemistry Instituto de Ciências Ambientais Químicas e Farmacêuticas Universidade Federal de São Paulo Rua Prof. Artur Riedel, 275, Diadema São Paulo 09972‐270 Brazil; ^2^ Department of Biological Sciences Instituto de Ciências Ambientais Químicas e Farmacêuticas Universidade Federal de São Paulo Rua Prof. Artur Riedel, 275, Diadema São Paulo 09972‐270 Brazil

**Keywords:** anxiolytic‐like effects, cannabidiol, Friedel–Crafts reactions, pharmacological studies, stereoselective synthesis

## Abstract

(−)‐Cannabidiol (CBD) is a nonpsychotropic phytocannabinoid found in *Cannabis* strains with well‐established pharmacological applications. Conversely, (+)‐cannabidiol (*ent*‐CBD), the non‐natural enantiomer of CBD, has been involved in a limited number of pharmacological studies. Therefore, CBD and *ent*‐CBD are synthesized using (*R*)‐(−)‐ and (*S*)‐(+)‐carvone as starting materials, respectively, via a highly diastereoselective 10‐camphorsulfonic acid (CSA) catalyzed Friedel–Crafts reaction as the key step. Pharmacological studies are conducted using the plus‐maze discriminative avoidance task (PM‐DAT) to evaluate the interaction between memory and anxiety‐like behavior as well as spontaneous motor activity in both young adult and aged male mice. The results show that *ent*‐CBD at a dose of 20 mg kg^−1^ has an anxiolytic‐like effect in aged male mice. In addition, *ent*‐CBD did not impair discriminative avoidance memory formation at all doses evaluated.

## Graphical abstract

Both (−)‐ and (+)‐cannabidiol (CBD and *ent*‐CBD, respectively) are synthesized via a highly diastereoselective CSA‐catalyzed Friedel–Crafts reaction. *ent*‐CBD exhibits anxiolytic‐like effects in aged male mice without impairing long‐term memory formation.

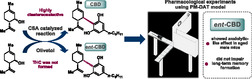

## 1 Introduction

**Scheme 1:**
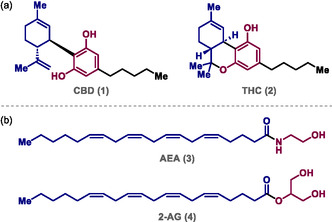
a) Structures of the phytocannabinoids CBD (**1**) and THC (**2**). b) Structures of the endocannabinoids AEA (**3**) and 2‐AG (**4**).

Marijuana (*Cannabis sativa* L.) has been used for over a thousand years for its therapeutic effects, attracting researchers worldwide.^[^ ^1^ ^]^ For example, several studies have reported its use in the treatment of Alzheimer's disease,^[^ ^2^ ^]^ Parkinson's disease,^[^ ^3^ ^]^ Crohn's disease,^[^ ^4^ ^]^ and multiple sclerosis.^[^ ^5^ ^]^ These uses are associated with compounds found in the plant called cannabinoids, molecules consisting of a monoterpene moiety attached to a resorcinol core, generally containing an alkyl chain.^[^ ^6^ ^]^ With over 100 cannabinoids present in cannabis, the two most abundant and well‐known are (−)‐cannabidiol (CBD, **1**), a nonpsychotropic compound, and (−)‐*trans*‐Δ^9^‐tetrahydrocannabinol (THC, **2**), a psychotropic substance^[^ ^6^, ^7^ ^]^ (**Scheme** **1**a).

The pharmacological properties of cannabinoids are related to the endocannabinoid system, a complex signaling system that regulates a range of neural processes, including cognition, memory, motivation, sleep, and pain, as well as immunological processes.^[^ ^8^ ^]^ The main receptors in this system are CB1 and CB2, the former being more abundant in the central nervous system^[^ ^9^ ^]^ and the latter in the peripheral nervous system.^[^ ^10^ ^]^ These receptors respond to the action of the endogenous cannabinoids anandamide (AEA, **3**) and 2‐arachidonoylglycerol (2‐AG, **4**),^[^ ^8^ ^]^ which are arachidonoyl acid derivatives (Scheme 1b). CBD (**1**) is known for its low affinity for CB1 and CB2 receptors, acting as their antagonist.^[^ ^11^ ^]^ Moreover, this phytocannabinoid does not act exclusively on CB1 and CB2 receptors, modulating the action of other receptors in the human body. Currently, the pharmaceutical use of CBD (**1**) is associated with its antiasthmatic,^[^ ^12^ ^]^ antiepileptic,^[^ ^13^ ^]^ anti‐inflammatory,^[^ ^14^ ^]^ anxiolytic‐like,^[^ ^15^ ^]^ antitumor,^[^ ^16^ ^]^ and chemoprotective^[^ ^17^ ^]^ properties. In 2018, Epidiolex, a CBD‐based drug manufactured by GW Pharmaceuticals, was approved by the US Food and Drug Administration (FDA) for the treatment of Lennox‐Gastaut syndrome and Dravet syndrome.^[^ ^13^ ^]^ In 2024, Wang, Chen, and coworkers reported results showing that the interaction of CBD (**1**) with the 5‐HT_1A_ receptor (in vitro and in vivo) mediates its anxiolytic‐like effect.^[^ ^15^ ^]^

**Scheme 2:**
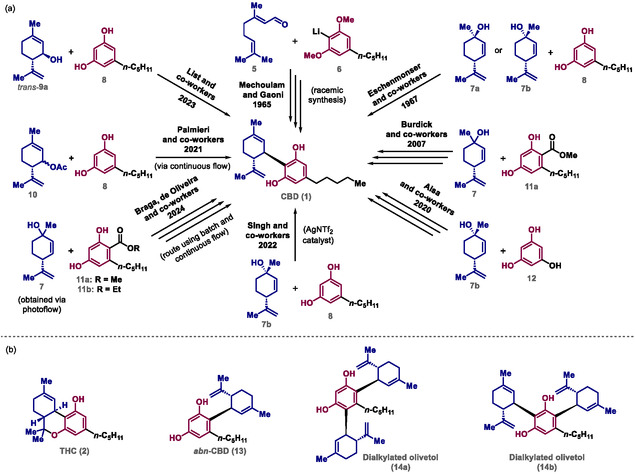
a) Synthetic routes to achieve CBD (**1**). b) Undesirable substances produced in CBD (**1**) syntheses.

Despite the importance of cannabinoids, current drug control policies in most countries prohibit the cultivation of marijuana because of its use for recreational purposes.^[^ ^18^ ^]^ In addition, agroclimatic factors and purification issues lead researchers to find alternative ways to obtain CBD (**1**) and other cannabinoids without the need to extract them from cannabis.^[^ ^19^ ^]^ Thus, the total synthesis of cannabinoids can be considered an important tool to obtain these compounds.^[^ ^6^, ^20^ ^]^ In 1965, Mechoulam and Gaoni reported the first total synthesis of (±)‐CBD [(±)‐**1**] using citral A (**5**) and a lithium derivative of olivetol dimethyl ether (**6**) as starting materials.^[^ ^21^, ^22^ ^]^ Several syntheses were developed after their publication,^[^ ^23^, ^24^, ^25^, ^26^, ^27^, ^28^, ^29^, ^30^, ^31^, ^32^, ^33^, ^34^ ^]^ among which we selected the first synthesis of enantiomerically pure CBD (**1**) by Eschenmoser and coworkers in 1967, involving Friedel–Crafts reactions between (+)‐*trans*‐ and (+)‐*cis*‐*p*‐mentha‐2,8‐dien‐1‐ol (**7a** and **7b**, respectively) and olivetol (**8**) in the presence of BF_3_ • OEt_2_, which allowed the direct formation of CBD (**1**).^[^ ^28^ ^]^ Over the years, other strategies somewhat related to Eschenmoser's approach^[^ ^28^ ^]^ have been applied to the synthesis of CBD (**1**). These include the use of isopiperitenol (*trans*‐**9a**)^[^ ^29^, ^30^ ^]^ or acetyl isopiperitenol (**10**)^[^ ^31^ ^]^ instead of alcohols **7**, alkyl olivetolates (**11**)^[^ ^32^, ^33^ ^]^ or phloroglucinol (**12**)^[^ ^34^ ^]^ instead of olivetol (**8**), and other Lewis acids such as AgNTf_2_^[^ ^19^ ^]^ (**Scheme** **2**a). Some of these approaches promote the formation of undesirable substances such as THC (**2**),^[^ ^28^, ^31^ ^]^ abnormal cannabidiol (*abn*‐CBD, **13**),^[^ ^28^, ^29^, ^31^ ^]^ and dialkylated olivetols (**14a,b**)^[^ ^28^, ^31^ ^]^ (Scheme 2b). THC (**2**) is obtained by intramolecular cyclization in acidic medium,^[^ ^23^, ^28^, ^31^ ^]^ and selectivity issues related to the Friedel–Crafts reaction favor the formation of *abn*‐CBD (**13**) and dialkylated olivetols (**14a,b**).^[^ ^23^, ^28^, ^31^ ^]^ Despite these problems, it is currently possible to synthesize natural and unnatural cannabinoids. Unnatural cannabinoids such as *ent*‐CBD (*ent*‐**1**), first synthesized in 1982,^[^ ^35^ ^]^ have been studied because of their greater affinity for CB1 and CB2 receptors compared to CBD (**1**).^[^ ^11^, ^36^ ^]^ Nevertheless, *ent*‐CBD (*ent*‐**1**) itself has been involved in a limited number of pharmacological studies.

In this contribution, CBD (**1**) and *ent*‐CBD (*ent*‐**1**) were synthesized using (*R*)‐(−)‐carvone (**15a**) and (*S*)‐(+)‐carvone (**15b**) as starting materials, respectively, by a simplified version of the refined route published by Maio and coworkers in 2021,^[^ ^37^ ^]^ through which *trans*:*cis* mixtures of isopiperitenol (**9a,b**) were synthesized. The development of a highly diastereoselective 10‐camphorsulfonic acid (CSA) catalyzed Friedel–Crafts reaction for the coupling of mixtures of **9a,b** with olivetol (**8**) completed the synthetic route.

Although a recent study has demonstrated that CBD (**1**) induces an anxiolytic‐like effect through the stress‐induced hyperthermia (SIH) model in male mice,^[^ ^15^ ^]^ we used the plus‐maze discriminative avoidance task (PM‐DAT) to further investigate the anxiolytic and cognitive properties of CBD (**1**) in young and aged male mice. While the effects of CBD (**1**) on anxiety‐like responses have been well documented,^[^ ^15^, ^38^ ^]^ its role in memory—both short‐ and long‐term—remains a subject of ongoing debate. To address this, we investigated the impact of different doses of CBD (**1**) and *ent*‐CBD (*ent*‐**1**), alongside diazepam (an agonist of the α subunit of the GABA_A_ receptor and a well‐established positive control known for its memory‐impairing effects) in male mice of different age groups.^[^ ^39^ ^]^ The plus‐maze discriminative avoidance task (PM‐DAT) has been used to explore anxiety‐like responses, hippocampal‐dependent memory, spontaneous motor activity, and their interactions in rodents under the effect of different drugs.^[^ ^39^, ^40^ ^]^ Our findings highlight the role of GABAergic modulation in this task, particularly involving the CA1 subfield of the hippocampus.^[^ ^41^, ^42^ ^]^ In addition, *ent*‐CBD (*ent*‐**1**) was included in the behavioral study due to its distinct interactions with receptors in the biological system when compared to CBD (**1**),^[^ ^11^, ^36^ ^]^ aiming to obtain a substance with a more pronounced anxiolytic‐like effect.

## 2 Results and Discussion

**Scheme 3:**
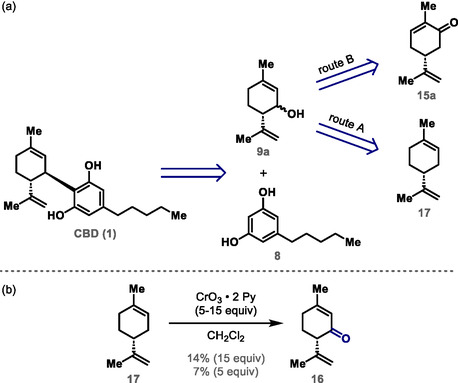
a) Retrosynthetic analysis for CBD (**1**). b) Oxidation of (*R*)‐(+)‐limonene (**17**) to (*S*)‐(+)‐isopiperitenone (**16**).

Initially, a diastereoselective synthesis of CBD (**1**) was planned by a coupling involving a *trans*:*cis* mixture of isopiperitenol (**9a**) and olivetol (**8**) revisited by our research group (*vide infra*). The synthesis of mixture **9a** was idealized by reduction of isopiperitenone (**16**), which would be obtained by selective oxidation of (*R*)‐(+)‐limonene (**17**)^[^ ^43^ ^]^ (route A) or by diastereoselective reactions starting with (*R*)‐(−)‐carvone (**15a**)^[^ ^37^ ^]^ (route B) (**Scheme** **3**a).

**Scheme 4:**
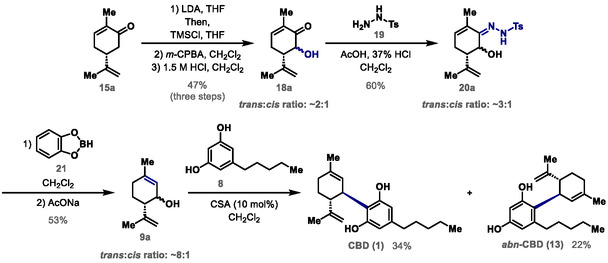
Diastereoselective synthesis of CBD (**1**).

Following *Route A* shown in the retrosynthetic analysis for CBD (**1**) (Scheme 3a), (*R*)‐(+)‐limonene (**17**) was subjected to oxidation with CrO_3_ (4 equiv) and *tert*‐butanol (10 equiv) in CCl_4_ under reflux for 3 h,^[^ ^43^ ^]^ but isopiperitenone (**16**) could not be isolated by column chromatography due to purification issues. The oxidation of (*R*)‐(+)‐limonene (**17**) to isopiperitenone (**16**) or directly to isopiperitenol (**9a**) was extensively studied using different oxidants (see Table S1 in the Supporting Information). However, the best result obtained was the oxidation of (*R*)‐(+)‐limonene (**17**) in the presence of an excess of Collins reagent (CrO_3_ • 2 Py) (15 equiv) in CH_2_Cl_2_ at room temperature for 24 h, which afforded isopiperitenone (**16**) in 14% isolated yield.^[^ ^44^ ^]^ When the same reaction was carried out in the presence of 5 equiv of Collins reagent, isopiperitenone (**16**) was isolated in 7% yield (Scheme 3b). These unpromising results led us to abandon *Route A* for the synthesis of CBD (**1**) (Scheme 3a). Considering *Route B* (Scheme 3a), the synthesis of CBD (**1**) was initiated by the three‐step conversion of (*R*)‐(−)‐carvone (**15a**) to intermediate **18a** in 47% yield as a *trans*:*cis* mixture with a ratio of ≈2:1. The diastereoisomeric mixture **18a** was subjected to condensation with *p*‐tosylhydrazide (**19**) in the presence of AcOH and 37% HCl to give intermediate **20a** in 60% yield as a *trans*:*cis* mixture with a ratio of ≈3:1. A one‐pot reduction/rearrangement of mixture **20a** was performed using catecholborane (**21**) and AcONa to afford isopiperitenol (**9a**) in 53% yield as a *trans*:*cis* mixture with a ratio of ≈8:1.^[^ ^37^ ^]^ In order to succeed in the key step of the synthesis, which consists of an acid‐catalyzed Friedel–Crafts reaction between mixture **9a** and olivetol (**8**), experiments were carried out using cyclohex‐2‐enol as a model compound and olivetol (**8**) in the presence of different acid catalysts. The best Brønsted acid was CSA (10 mol%) (see Table S2 in the Supporting Information), which allowed the coupling between the *trans*:*cis* mixture **9a** and olivetol (**8**) to afford CBD (**1**) and *abn*‐CBD (**13**) in yields of 34% and 22%, respectively (**Scheme** **4**). Furthermore, the most effective Lewis acids tested during the optimization were TMSOTf and AgOTf. The third‐best performance was obtained with FeCl_3_·6H_2_O, which suggests a potential limitation of the model compound used, given that this iron catalyst has previously proven effective in CBD synthesis^[^ ^45^ ^]^ (see Table S2 in the Supporting Information). Thus, when the coupling between the *trans*:*cis* mixture **9a** and olivetol (**8**) was performed with TMSOTf (20 mol%), only *abn*‐CBD (*abn*‐**13**) could be isolated in 10% yield (result not shown). As an alternative to TMSOTf, we used 20 mol% of AgOTf for the Friedel–Crafts reaction and obtained CBD (**1**) and *abn*‐CBD (**13**) in 30% yield each (see Supporting Information). In the presence of an appropriate acid, both *trans*‐**9a** and *cis*‐**9a** diastereoisomers generate the same carbocation intermediate, which reacts diastereoselectively with olivetol (**8**), leading exclusively to the formation of CBD (**1**) with a *trans* substitution pattern on the cyclohexene ring. The reactions with CSA and AgOTf did not produce dialkylated products (**14a,b**) or THC (**2**), possibly due to their short times, but we could not avoid the formation of *abn*‐**13**.

**Scheme 5:**
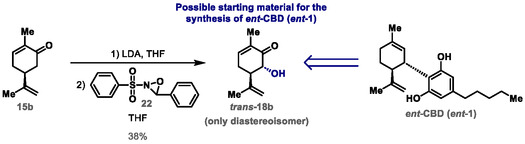
Diastereoselective synthesis of *trans*‐α‐hydroxycarvone (*trans*‐**18b**) targeting *ent*‐CBD (*ent*‐**1**).

To optimize the α‐hydroxylation of carvone shown in Scheme 4, we performed several experiments to convert (*S*)‐(+)‐carvone (**15b**) to α‐hydroxycarvone (**18b**) by a Davis oxidation targeting *ent*‐CBD (*ent*‐**1**)^[^ ^46^ ^]^ (see Table S3 in the Supporting Information). The best result for the transformation allowed the exclusive formation of *trans*‐α‐hydroxycarvone (*trans*‐**18b**) in 38% isolated yield. This was achieved when the enolate of compound **15b** was generated with 1 equiv of LDA, initially at 0 °C, and then at room temperature for 30 min, followed by reaction with 1.5 equiv of oxaziridine **22** (Davis reagent) at −78 °C for 60 min (**Scheme** **5**). Although the transformation shown in Scheme 5 is highly diastereoselective and leads exclusively to the formation of compound *trans*‐**18b**, we used the synthetic route shown in Scheme 4 from (*S*)‐(+)‐carvone (**15b**) to synthesize *ent*‐CBD (*ent*‐**1**).

**Figure 1:**
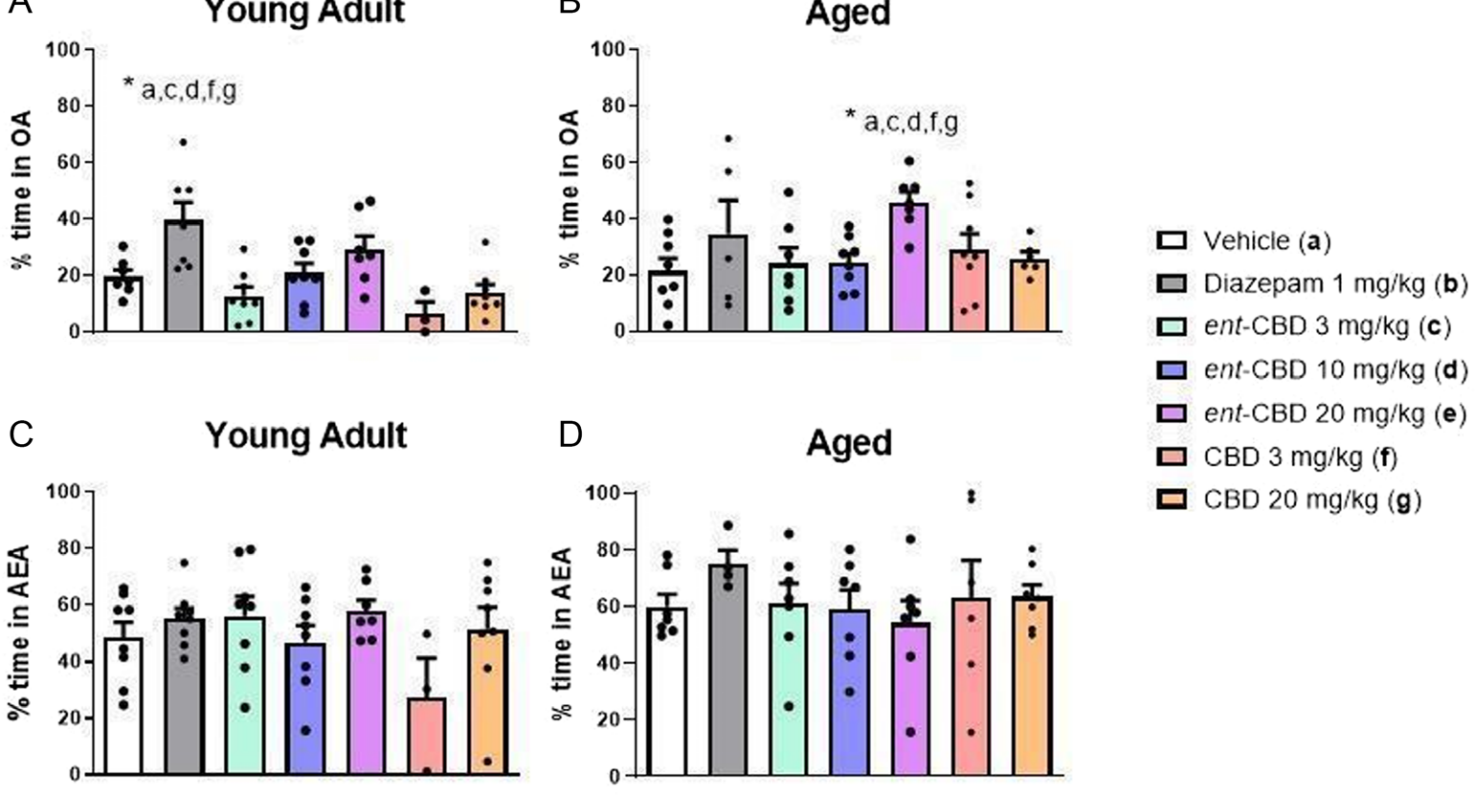
A) Percentage of time in open arms (OA) for treatments (**a–g**) with young adult male mice (3–6 months old). B) Percentage of time in open arms (OA) for treatments (**a–g**) with aged male mice (15–18 months old). C) Percentage of time in the aversive enclosed arm (AEA) for treatments (**a–g**) with young adult male mice (3–6 months old). D) Percentage of time in the aversive enclosed arm (AEA) for treatments (**a–g**) with aged male mice (15–18 months old). Images (A) and (B) correspond to the training session. Images (C) and (D) correspond to the test session.

Pharmacological effects of CBD (**1**) and *ent*‐CBD (*ent*‐**1**) in young and aged male mice were conducted using PM‐DAT (**Figure** **1**) to assess memory, anxiety, spontaneous motor activity, and their respective interactions.^[^ ^39^ ^]^ Despite recommendations to include both male and female mice in behavioral experiments,^[^ ^47^ ^]^ intrinsic genetic and hormonal differences, as well as age‐related sex‐specific variations, can significantly affect responses to cannabinoid treatments such as CBD (**1**),^[^ ^48^ ^]^ which seems to increase anxiety in female mice.^[^ ^49^ ^]^ Therefore, to characterize the effects of CBD (**1**) on anxiety‐like responses, spontaneous motor activity, and memory, this study focused on male mice. We administered synthesized cannabinoids in doses ranging from 3 to 20 mg kg^−1^, which is within the ideal range for anxiety studies with CBD (**1**), as noted by Guimarães and co‐workers.^[^ ^38^, ^50^ ^]^ This dosage range is within the inverted U‐shaped dose‐response curve observed in behavioral experiments with CBD (**1**) in rats, indicating that doses below 2.5 mg kg^−1^ and above 20 mg kg^−1^ are ineffective.^[^ ^50^ ^]^

The anxiolytic‐like effect was assessed by the percentage of time each animal spent in open arms (OA) during the training session, as mice naturally prefer protected, unlit spaces and have an aversion to unprotected, lit spaces.^[^ ^39^ ^]^ Figure 1A shows that young adult male mice (3–6 months old) treated with 1 mg kg^−1^ of diazepam (**b**) spent more time in OA than those treated with vehicle (**a**), *ent*‐CBD (*ent*‐**1**) at doses of 3 mg kg^−1^ (**c**) and 10 mg kg^−1^ (**d**), and CBD (**1**) at doses of 3 mg kg^−1^ (**f**) and 20 mg kg^−1^ (**g**), demonstrating the anxiolytic effect of diazepam. In addition, treatment with *ent*‐**1** at a dose of 20 mg kg^−1^ (**e**) was not significantly different from vehicle (**a**) and diazepam (1 mg kg^−1^) (**b**) [F_(6,41)_ = 6.399; *P* < 0.0001]. In Figure 1B, aged male mice (15–18 months old) treated with 20 mg kg^−1^ of *ent*‐CBD (*ent*‐**1**) spent considerable time in OA, supporting the anxiolytic properties of *ent*‐**1** (**e**). No significant difference was observed between the aged mice treated with *ent*‐**1** at a dose of 20 mg kg^−1^ (**e**) and those treated with diazepam (1 mg kg^−1^) (**b**). Further, no significant difference was found between vehicle‐treated mice (**a**) and those treated with *ent*‐**1** at doses of 3 mg kg^−1^ (**c**) and 10 mg kg^−1^ (**d**), and with CBD (**1**) at doses of 3 mg kg^−1^ (**f**) and 20 mg kg^−1^ (**g**) [F_(6,42)_ = 2.440; *P* = 0.0409]. The results indicate that the anxiolytic effect of *ent*‐**1** at the dose of 20 mg kg^−1^ (**e**) is more pronounced in older male mice compared to younger male mice [F_(3,26)_ = 25.53; *P* ≤ 0.0001] [the corresponding vehicles (**a**) were included in the analysis]. The findings related to *ent*‐**1** suggest that further experiments, including molecular analysis of brain structures underlying anxiety and long‐term memory formation, as well as toxicological studies, are required before *ent*‐**1** can be considered as a potential drug candidate. Furthermore, mice treated with CBD (**1**) showed no considerable anxiolytic‐like effect (Figure 1A,B). The lack of anxiolytic‐like effects for CBD (**1**) is in contrast to the literature,^[^ ^15^ ^]^ but different animal models, doses, and treatment times were used [stress‐induced hyperthermia (SIH) (doses: 1, 3, and 10 mg kg^−1^)^[^ ^15^ ^]^ versus PM‐DAT (doses: 3, 10, and 20 mg kg^−1^)], both using intraperitoneal (IP) injections to administrate the drugs.

The effects of *ent*‐CBD (*ent*‐**1**) at doses of 3 mg kg^−1^ (**c**), 10 mg kg^−1^ (**d**), and 20 mg kg^−1^ (**e**) and CBD (**1**) at doses of 3 mg kg^−1^ (**f**) and 20 mg kg^−1^ (**g**) on the long‐term discriminative avoidance memory of young male mice (Figure 1C) and aged male mice (Figure 1D) were evaluated by the percentage of time spent in the aversive enclosed arm (AEA) during the test session.^[^ ^39^ ^]^ No effects of treatments were found in both young [F_(6,43)_ = 1.512; *P* = 0.973] and aged [F_(6,41)_ = 0.6622; *P* = 0.6835] mice, suggesting that *ent*‐**1** and CBD (**1**) did not impair long‐term memory formation at all doses evaluated. The number of entries in the nonaversive enclosed arm (NAEA) was used as a measure of spontaneous motor activity and was evaluated during the training session under the effect of the drugs. *ent*‐CBD (*ent*‐**1**) at a dose of 20 mg kg^−1^ increased the number of entries (exploratory behavior) of young mice compared to all groups [F_(6,43)_ = 7.590; *P* < 0.0001]. Moreover, aged mice treated with 1 mg kg^−1^ of diazepam (**b**) exhibited a significantly reduced number of entries in the NAEA compared to aged mice treated with *ent*‐**1** at doses of 3 mg kg^−1^ and 10 mg kg^−1^ [F_(6,41)_ = 3.223; *P* = 0.0110]. Aged mice treated with *ent*‐**1** at a dose of 20 mg kg^−1^ did not differ in the number of entries in the NAEA from the aged mice treated with vehicle (**a**) (results not shown). In addition, experiments showed that CBD (**1**) at a dose of 3 mg kg^−1^ (**f**) had a sedative effect in more than half of the young male mice (Figure 1A,C). Animals that did not explore the maze were excluded from data analysis.

## 3 Conclusions

In summary, CBD (**1**) and *ent*‐CBD (*ent*‐**1**) were synthesized using (*R*)‐(−)‐carvone (**15a**) and (*S*)‐(+)‐carvone (**15b**) as starting materials, respectively, by a route that involved a highly diastereoselective CSA catalyzed Friedel–Crafts reaction for the coupling of *trans*:*cis* mixtures of isopiperitenol (**9a,b**) with olivetol (**8**). The use of isopiperitenol mixtures (**9a,b**) has the advantage of reducing the number of purification steps compared to routes that separate the diastereoisomers of alcohol **9**. The *trans*‐**9** and *cis*‐**9** compounds in the presence of CSA lead to the formation of the same carbocation, which reacts in a highly diastereoselective manner with olivetol (**8**), promoting the exclusive formation of the *trans* diastereoisomer of the cannabinoid of interest (**1** and *ent*‐**1**). No dialkylated products (**14a,b**) or THC (**2**) were generated through this route, but the formation of *abn*‐CBD (*abn*‐**13**) could not be avoided due to regioselectivity problems of the Friedel–Crafts reaction. The use of CSA represents a metal‐free option for obtaining CBD (**1**) and *ent*‐CBD (*ent*‐**1**). Pharmacological studies using the PM‐DAT assessed anxiolytic‐like properties and memory interactions of CBD (**1**) and *ent*‐CBD (*ent*‐**1**) in young adult and aged male mice. The results indicated that CBD (**1**) did not exhibit significant anxiolytic‐like effect in mice at any of the tested doses. Conversely, aged mice treated with *ent*‐**1** at a dose of 20 mg kg^−1^ displayed a notable anxiolytic‐like effect. In addition, neither CBD (**1**) nor *ent*‐**1** impaired long‐term memory formation in both young and aged mice. Further experiments with *ent*‐**1**, including molecular analysis of brain structures and toxicological studies, may help to understand its effects and potential as a drug candidate.

## 4 Experimental Section

4.1

4.1.1

##### Synthetic Procedure

*Preparation of (−)‐cannabidiol (CBD, **1**) and (+)‐cannabidiol (ent‐CBD, ent‐**1**)*: CSA (23.2 mg, 0.1 mmol, 10 mol%) was added to a round‐bottomed flask (25 mL), which was quickly sealed with a rubber septum and maintained under a nitrogen atmosphere. A solution of olivetol (**8**) (196 mg, 1.1 mmol, 1.1 equiv) in dichloromethane (7.5 mL) was added and magnetic stirring was started. After 1 min, a solution of the appropriate *trans*:*cis* mixture of isopiperitenol (**9**) (152 mg, 1 mmol) in dichloromethane (7.5 mL) was added dropwise. The reaction mixture was kept at room temperature under magnetic stirring and a nitrogen atmosphere for 2 h. Afterward, a saturated solution of NaHCO_3_ (15 mL) was added to the reaction. The aqueous phase was extracted with dichloromethane (3 × 15 mL). The organic phase was washed with brine (15 mL) and dried over Na_2_SO_4_. After filtration, the solvent was removed under reduced pressure. The material was purified by column chromatography on silica gel 60 using hexane/ethyl acetate (85:15) as eluent to give CBD (**1**) and *abn*‐CBD (**13**) or *ent*‐CBD (e*nt*‐**1**) and *ent*‐*abn*‐CBD (*ent*‐**13**).

*(−)‐Cannabidiol (CBD, **1**) (CAS Number: 13956‐29‐1)*: Yield: 107 mg, 34%; yellowish oil; R_*f*_ = 0.53 [hexane/ethyl acetate (85:15)]; ${\left[\alpha \right]}_{D}^{23}$ = −124.4 (*c* = 1.0, EtOH) {lit.^19^ ${\left[\alpha \right]}_{D}^{20}$ = −124 (*c* = 1.0, EtOH)}. ^1^H NMR (300 MHz, CDCl_3_): *δ* 6.32‐6.13 (m, 2 H), 5.97 (br., 1H), 5.57 (s, 1H), 4.80‐4.72 (br., 1H), 4.67‐4.63 (m, 1H), 4.56 (s, 1H), 3.90‐3.82 (m, 1H), 2.46‐2.35 (m, 3 H), 2.28‐2.05 (m, 2 H), 1.85‐1.74 (m, 5 H), 1.66 (s, 3 H), 1.60‐1.50 (m, 2 H), 1.33‐1.25 (m, 4 H), 0.88 (t, *J* = 6.6 Hz, 3 H); ^13^C NMR (75 MHz, CDCl_3_): *δ* 155.9, 154.0, 149.2, 143.0, 139.9, 124.1, 113.8, 110.8, 109.6, 108.0, 46.2, 37.1, 35.5, 31.5, 30.6, 30.4, 28.3, 23.6, 22.5, 20.3, 14.0; IR (KBr, cm^−1^): 3435, 2957, 2926, 2857, 2833, 1630, 1583, 1445, 1377, 1217, 1026; GC/MS (*m/z*, %): 314 (9.8), 299 (4.9), 246 (11.8), 231 (100.0), 193 (7.5), 174 (9.1), 121 (7.5), 91 (4.6). Characterization data are in accordance with the literature.^[^ ^19^ ^]^

*(+)‐Cannabidiol (ent‐CBD, ent‐**1**) (CAS Number: 74219‐29‐7):* Yield: 97.4 mg, 31%; yellowish oil; R_*f*_ = 0.53 [hexane/ethyl acetate (85:15)]; ${\left[\alpha \right]}_{D}^{23}$ = +121.0 (*c* = 1.0, EtOH) {lit.^29^ ${\left[\alpha \right]}_{D}^{20}$ = +124 (c = 1.0, EtOH)}. ^1^H NMR (300 MHz, CDCl_3_): *δ* 6.32–6.13 (m, 2H), 5.97 (br., 1H), 5.56 (s, 1H), 4.68–4.65 (m, 1H), 4.55 (s, 1H), 3.89–3.82 (m, 1H), 2.46–2.35 (m, 3H), 2.24–2.04 (m, 2H), 1.86–1.74 (m, 5H), 1.65 (s, 3H), 1.60–1.49 (m, 2H), 1.35–1.25 (m, 4H), 0.88 (t, *J* = 6.6 Hz, 3H). ^13^C NMR (75 MHz, CDCl_3_): *δ* 156.0, 153.9, 149.3, 143.0, 140.0, 124.0, 113.7, 110.8, 109.7, 108.0, 46.0, 37.2, 35.4, 31.4, 30.6, 30.3, 28.3, 23.6, 22.5, 20.4, 14.0. IR (KBr, cm^−1^): 3427, 2956, 2926, 2856, 2830, 1624, 1585, 1446, 1377, 1217, 1026. GC/MS (*m/z*, %): 314 (8.9), 299 (6.3), 246 (8.9), 231 (100.0), 193 (8.4), 174 (13.0), 121 (8.9), 91 (12.9). Characterization data are in accordance with the literature.^[^ ^37^ ^]^

##### Behavioral Procedure

The PM‐DAT is an adaptation of the well‐established elevated plus maze, which has been used to assess hippocampal‐dependent memory, anxiety‐like behavior, and spontaneous motor activity in mice. Animals were individually placed in the PM‐DAT apparatus (see Figure S37 in the Supporting Information). During the training session (acquisition memory), mice were placed in the center of the apparatus facing an open arm (OA) and their spontaneous locomotor activity in all arms was recorded for 10 min. Upon entering the aversive enclosed arm (AEA) with all paws, aversive stimuli (a 100 W light and 85 dB noise) were simultaneously activated and remained on until the animal exited the AEA. Twenty‐four hours later, during the test session (evocation memory), the mice were reintroduced to the apparatus and allowed to explore for 3 min without any aversive stimuli; only cues proximal and distal were maintained. Both sessions were recorded with a digital camera mounted on the ceiling, and the light level in the room was controlled throughout. The apparatus was disinfected with 10% ethanol before each new animal was introduced for testing.^[^ ^39^ ^]^

##### Behavioral Analysis

Anxiety‐like behavior was assessed by calculating the percentage of time spent in open arms (OA) [% time in OA = (time spent in OA/total time spent in all arms) × 100]. Memory performance was assessed by calculating the percentage of time spent in the aversive enclosed arm (AEA) [% time in AEA = (time spent in AEA/total time spent in both enclosed arms) × 100] during the test session, reflecting long‐term memory retention.^[^ ^39^ ^]^ Spontaneous motor activity was evaluated using the number of entries into nonaversive enclosed arm (NAEA), as anxiety can reduce overall motor activity and decrease both the percentage of time spent in and the number of entries into aversive arms (open and enclosed).

## Conflict of Interest

The authors declare no conflict of interest.

## Supporting information

Supplementary Material

## Data Availability

General methods and experimental procedures for the preparation of compounds, characterization data, NMR spectra, Table S1–S4, and description of pharmacological assessments [animals, drugs and administration, apparatus (Figure S37, Supporting Information), handling, behavioral procedure, behavioral analysis, and statistical analysis] have been included in the Supporting Information.
