# Supplementary material for: Stereoselective Synthesis of Cannabidiol and its Unnatural Enantiomer to Evaluate Anxiolytic‐Like Properties and Memory Interactions
Source: Chembiochem. 2025 Jul 24;26(17):e202500438. doi: 10.1002/cbic.202500438 (PMC12442214; doi:10.1002/cbic.202500438)
Supplement: Supplementary file 1 — Supplementary Material [file CBIC-26-e202500438-s001.pdf]

Supporting Information

Stereoselective Synthesis of Cannabidiol and Its Unnatural Enantiomer to Evaluate Anxiolytic-like Properties and Memory Interactions

Marcos Accioly Jr, Kawana Rosa, Andressa G. Soliani, Suzete M. Cerutti, and Cristiano Raminelli*

Instituto de Ciências Ambientais, Químicas e Farmacêuticas, Universidade Federal de São Paulo, Diadema, São Paulo 09972-270, Brazil

*Corresponding author. Email address: raminelli@unifesp.br

Table of Contents

General methods.. 2S
Table S1.. 3S
Preparation of (*S*)-(+)-isopiperitenone (**16**).. 4S
Preparation of Collins reagent (CrO_3 • 2 Py).. 4S
Preparation of *trans*- and *cis*-(5*S*)-6-hydroxy-2-methyl-5-(prop-1-en-2-yl)cyclohex-2-enone (*trans*-**18a** and *cis*-**18a**) and *trans*- and *cis*-(5*R*)-6-hydroxy-2-methyl-5-(prop-1-en-2-yl)cyclohex-2-enone (*trans*-**18b** and *cis*-**18b**).. 5S
Preparation of *p*-tosylhydrazide (**19**).. 7S
Preparation of *trans*- and *cis*-*N*'-((5*S*)-6-hydroxy-2-methyl-5-(prop-1-en-2-yl)cyclohex-2-en-1-ylidene)-4-methylbenzenesulfonohydrazide (*trans*-**20a** and *cis*-**20a**) and *trans*- and *cis*-*N*'-((5*R*)-6-hydroxy-2-methyl-5-(prop-1-en-2-yl)cyclohex-2-en-1-ylidene)-4-methylbenzenesulfonohydrazide (*trans*-**20b** and *cis*-**20b**).. 8S
Preparation of *trans*- and *cis*-(6*S*)-isopiperitenol (*trans*-**9a** and *cis*-**9a**) and *trans*- and *cis*-(6*R*)-isopiperitenol (*trans*-**9b** and *cis*-**9b**).. 10S
Preparation of cyclohex-2-enol (**24**).. 13S
Table S2.. 14S
Preparation of 2-(cyclohex-2-enyl)-5-pentylbenzene-1,3-diol (**26**) and 4-(cyclohex-2-enyl)-5-pentylbenzene-1,3-diol (**27**).. 15S
Preparation of (-)-cannabidiol (CBD, **1**) and (-)-abnormal cannabidiol (*abn*-CBD, **13**) and (+)-cannabidiol (*ent*-CBD, *ent*-**1**) and (+)-abnormal cannabidiol (*ent*-*abn*-CBD, *ent*-**13**)............ 16S
Preparation of *N*-benzylidenebenzenesulfonamide (**28**).. 19S
Preparation of 3-phenyl-2-(phenylsulfonyl)-1,2-oxaziridine (**22**).. 20S
Table S3.. 21S
Preparation of *trans*-(5*R*,6*R*)-6-hydroxy-2-methyl-5-(prop-1-en-2-yl)cyclohex-2-enone (*trans*-**18b**).. 22S
^{1}H and ^{13}C NMR spectra.. 23S
Determination of ^{1}H NMR yields.. 59S
Table S4.. 59S
Biological assays.. 60S
Animals.. 60S
Drugs and administration.. 60S
Apparatus.. 60S
Handling.. 61S
Behavioral procedure.. 61S
Behavioural analysis.. 62S
Statistical analysis.. 62S
References.. 63S

General methods

Chemicals were purchased from commercial suppliers and used without purification unless otherwise stated. Dichlorometane, diisopropylamine (DIPA), hexamethylphosphoramide (HMPA), and trimethylsilyl chloride (TMSCl) were distilled from calcium hydride prior to use.[51,52] Diethyl ether and tetrahydrofuran (THF) were distilled from sodium/benzophenone under a nitrogen atmosphere before use. [51,52] *n*-Butyllithium (*n*-BuLi) was titrated against isopropanol using 1,10-phenanthroline as an indicator under a nitrogen atmosphere.[53] Lithium diisopropylamide (LDA) was generated following typical procedure prior to use.[54] Molecular sieves (4Å) were activated before use. Purifications by column chromatography were performed using 70-230 mesh silica gel 60. Organic solvents were evaporated under reduced pressure in a rotary evaporator. Melting point values are uncorrected. The values of specific rotations ($[\alpha]_D^T$) were determined in a digital polarimeter, equipped with a sodium lamp (589 nm), using a cell with an optical path of 50 mm. ^{1}H and ^{13}C NMR spectra were acquired using a NMR spectrometer operating at 300 and 75 MHz, respectively. ^{1}H and ^{13}C NMR spectra were obtained in $CDCl_3$ at 298 K and the chemical shifts reported in ppm with respect to tetramethylsilane (TMS) used as an internal standard or with respect to the deuterated solvent used as a reference. Infrared spectra were obtained employing attenuated total reflectance (ATR) or KBr pellets in the wavenumber range of 4000-400 cm^{-1}. Mass spectra were carried out using a gas chromatograph connected to a mass spectrometer with electron impact ionization at 70 eV. High-resolution mass spectra were obtained employing a time-of-flight mass spectrometer.

Table S1. Reactions aiming at the preparation of (*S*)-(+)-isopiperitenone (**16**) or isopiperitenol (**9a**).[a]

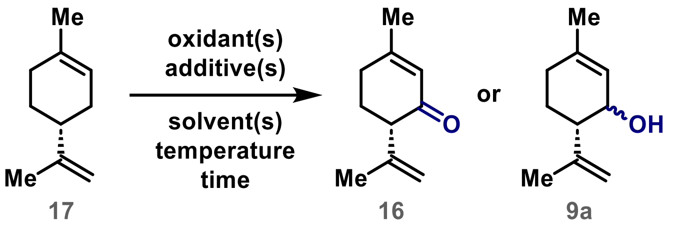

Entry	Oxidant(s) (equiv)	Additive(s) (equiv)	Solvent(s)	Temp. (°C)	Time (h)	Isolated yield of **16** (%)	Isolated yield of **9a** (%)
1[43]	CrO_3 (4)	*t*-BuOH (10)	CCl_4	reflux	3	0[b,c]	-
2[43]	CrO_3 (4)	*t*-BuOH (10)	CH_2Cl_2	reflux	3	0[d]	-
3[55]	CrO_3 (4)	*t*-BuOH (8) and Ac_2O (10)	CH_2Cl_2/AcOH (3:1)	r.t.	12	0[e,c]	-
4[44]	CrO_3 • 2 Py (15)	-	CH_2Cl_2	r.t.	24	14[c]	-
5[44]	CrO_3 • 2 Py (5)	-	CH_2Cl_2	r.t.	24	7[c]	-
6[56]	SeO_2 (1.1)	-	1,4-dioxane	85	0.5	-	0[f]
7[57]	SeO_2 (0.5)	TBHP in H_2O (4)	CH_2Cl_2	r.t.	24	-	0[f]
8[58]	NaClO (2)	TBHP in H_2O (7) and K_2CO_3 (0.1)	CH_3CN/H_2O (3:1)	5	9	0[b,g]	-
9[59]	TBHP in H_2O (7)	CuI (0.01)	CH_3CN	50	24	0[b,g]	-
10[60]	TBHP in decane (4)	CuBr (0.01)	CH_3CN	50	24	0[g]	-
11[61]	TBHP in decane (4)	Pd/C (0.1) and K_2CO_3 (0.2)	CH_2Cl_2	0 to r.t.	15	0[g]	-
12[62]	TBHP in decane (4) and O_2	$Mn(OAc)_3$ (0.1)	EtOAc	r.t.	48	0[g]	-

[a] Reaction conditions: (*R*)-(+)-limonene (**16**) (5 mmol), oxidant(s), additive(s), and solvent(s) were stirred at the indicated temperature for the indicated time. [b] Compound **16** was formed (TLC and GC/MS), but compound **16** could not be isolated with satisfactory purity. [c] (*S*)-(+)-Carvone was formed (TLC and GC/MS). [d] A small amount of compound **16** was formed (TLC and GC/MS). [e] A complex mixture containing compound **16** was formed (TLC and GC/MS) and compound **16** could not be isolated. [f] A complex mixture containing a product of *m/z* 152 was formed (GC/MS) and this product of *m/z* 152 could not be isolated. [g] A complex mixture containing (*S*)-(+)-carvone was formed (TLC and GC/MS) and (*S*)-(+)-carvone could not be isolated.

Preparation of (*S*)-(+)-isopiperitenone (16)[43]

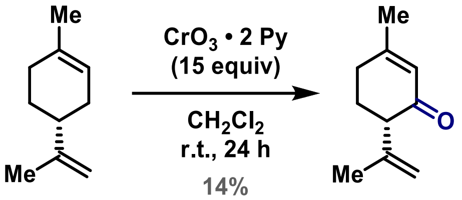

17 16

Dichloromethane (70 mL) was added to a round-bottomed flask (250 mL) containing (*R*)-(+)-limonene (**17**) (680 mg, 5 mmol). Then, a slurry of Collins reagent (CrO_3 • 2 Py) (23.1 g, 74.8 mmol, 15 equiv) in dichloromethane (40 mL) was carefully added. The reaction mixture was magnetically stirred at room temperature for 24 h. Afterwards, the mixture was washed with brine (50 mL) and the aqueous phase was extracted with dichloromethane (3 x 50 mL). The organic phase was washed with a saturated solution of $CuSO_4$ (2 x 50 ml) and with water (2 x 50 ml) and then dried over Na_2SO_4. After filtration, the solvent was evaporated under reduced pressure. The material obtained was purified by column chromatography on silica gel 60 using hexane/ethyl acetate (4:1) as eluent to afford (*S*)-(+)-isopiperitenone (**16**).

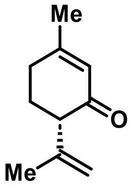

16

(*S*)-(+)-isopiperitenone (16) (CAS Number: 16750-82-6): Yield: 97.4 mg, 14%; pinkish oil; R_f = 0.39 [hexane/ethyl acetate (4:1)]; $[\alpha]_D^{23}$ = +30.4 (*c* = 0.54, $CHCl_3$) {lit.[43] $[\alpha]_D^{28}$ = +27.9 (*c* = 0.54, $CHCl_3$)}. **^{1}H NMR (300 MHz, $CDCl_3$):** δ 5.85 (m, 1H), 4.92-4.87 (m, 1H), 4.71 (s, 1H), 2.90 (dd, *J* = 10.4, 5.2 Hz, 1H), 2.33-2.26 (m, 2H), 2.09-1.97 (m, 2H), 1.91 (s, 3H), 1.70 (s, 3H). **^{13}C NMR (75 MHz, $CDCl_3$):** δ 199.0, 162.0, 143.2, 126.5, 113.4, 53.6, 30.2, 27.5, 24.1, 20.5. **IR (ATR, cm^{-1}):** 3076, 2972, 2924, 2826, 1661, 1435, 1379, 1200, 1024, 889, 876, 763. **GC/MS (*m/z*, %):** 150 (9.1), 135 (20.4), 122 (5.6), 107 (6.6), 91 (5.6), 82 (100), 67 (6.2), 54 (22.8), 41 (7.8). Characterization data are in accordance with the literature.[43]

Preparation of Collins reagent (CrO_3 • 2 Py)[44]

Anhydrous pyridine (Py) (90 mL, 1.2 mol, 12 equiv) was added to a two-neck round-bottomed flask (250 mL) through one of the necks, which was closed with a rubber septum. The round-bottomed flask was cooled to -15 °C under magnetic stirring and chromium trioxide (CrO_3) (10.0 g, 0.1 mol) was carefully added through the other neck, which was

connected to a drying tube. *Caution! Avoid reaction of CrO_3 with pyridine at room temperature, which is extremely exothermic and spontaneously ignites.* The reaction mixture was magnetically stirred at -15 °C for 6 h. Afterwards, the mixture was vacuum filtered and the solid obtained was dried in a vacuum desiccator over phosphorus pentoxide for 24 h. CrO_3 • 2 Py was obtained (23.2 g, 90%) and used without further purification.

Preparation of *trans*- and *cis*-(5*S*)-6-hydroxy-2-methyl-5-(prop-1-en-2-yl)cyclohex-2-enone (*trans*-18a and *cis*-18a) and *trans*- and *cis*-(5*R*)-6-hydroxy-2-methyl-5-(prop-1-en-2-yl)cyclohex-2-enone (*trans*-18b and *cis*-18b)[37,63]

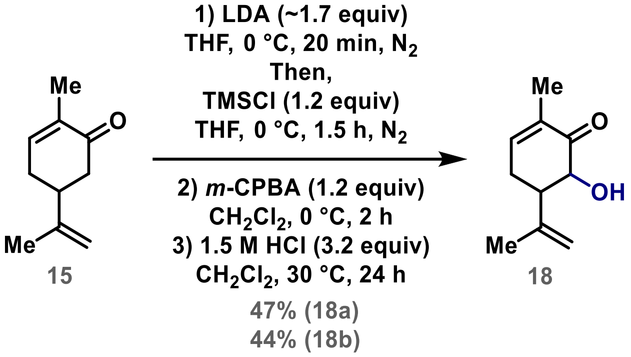

THF (45 mL) was added to a round-bottomed flask (250 mL) containing DIPA (4.8 mL, 33.4 mmol, 1.7 equiv). The round-bottomed flask was cooled to -78 °C under magnetic stirring and a nitrogen atmosphere. *n*-BuLi (28.2 mL of a 1.24 mol.L^{-1} solution in hexane, 35 mmol, 1.8 equiv) was added and the mixture was heated to 0 °C. After 1.5 h, a solution of the appropriate carvone (**15**) (3.00 g, 20 mmol) in THF (35 mL) was added and the reaction was maintained at 0 °C. After 20 min, TMSCl (6 mL, 24 mmol, 1.2 equiv.) was added and the mixture was maintained at 0 °C under magnetic stirring and a nitrogen atmosphere for 1.5 h. Brine (25 mL) and a saturated solution of $NaHCO_3$ (25 mL) were then added to the reaction mixture. The aqueous phase was extracted with ethyl acetate (3 x 100 mL). The organic phase was dried over Na_2SO_4. After filtration, the solvent was evaporated under reduced pressure. The material obtained was used in the next step without purification.

Dichloromethane (100 mL) was added to a round-bottomed flask (250 mL) containing the material from the first step. The round-bottomed flask was cooled to 0 °C and *m*-chloroperbenzoic acid (*m*-CPBA) <77% (4.50 g, 24 mmol, 1.2 equiv) was added in portions under magnetic stirring over 2 h. A saturated solution of Na_2SO_3 (100 mL) was then added to the reaction mixture. The organic phase was washed with a saturated solution of $NaHCO_3$ (2 x 100 mL). The aqueous phase was extracted with dichloromethane (2 x 50 mL). The

organic phase was dried over Na_2SO_4. After filtration, the solvent was evaporated under reduced pressure. The material obtained was used in the next step without purification.

Dichloromethane (50 mL) was added to a round-bottomed flask (250 mL) containing the material from the second step. The round-bottomed flask was heated to 30 °C and a 1.5 mol.L^{-1} solution of HCl (42.7 mL, 64 mmol, 3.2 equiv) was added. The reaction was magnetically stirred at 30 °C for 24 h. Afterwards, the reaction mixture was neutralized using a 5% (w/v) solution of NaOH and washed with brine (2 x 50 mL). The aqueous phase was extracted with dichloromethane (3 x 100 mL). The organic phase was dried over Na_2SO_4. After filtration, the solvent was evaporated under reduced pressure. The residue obtained was purified by column chromatography on silica gel 60 using hexane/ethyl acetate (80:20) as eluent to give the diastereoisomeric mixture containing either ***trans*-18a** and ***cis*-18a** or ***trans*-18b** and ***cis*-18b**.

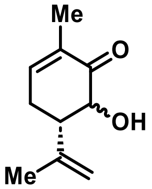

18a

trans*- and *cis*-(5*S*)-6-Hydroxy-2-methyl-5-(prop-1-en-2-yl)cyclohex-2-enone (*trans*-18a and *cis*-18a) (CAS Number: 144850-52-2, *trans*-18a; 168039-50-7, *cis*-18a):** Yield: 1.57 g, 47% (three steps); *trans*:*cis* ratio = ~2:1 (GC/MS and ^{1}H NMR); yellowish oil; R_f = 0.36 and 0.27 (*trans* and *cis*, respectively) [hexane/ethyl acetate (80:20)]. **^{1}H NMR (300 MHz, CDCl$_3$):** δ 6.79-6.73 (m, 1H, ***trans*-18a**), 6.70-6.65 (m, 0.45H, ***cis*-18a**), 4.96-4.93 (m, 2H, ***trans*-18a**), 4.88-4.84 (m, 0.45H, ***cis*-18a**), 4.71 (s, 0.45H, ***cis*-18a**), 4.43 (d, *J* = 5.9 Hz, 0.45H, ***cis*-18a**), 4.16 (d, *J* = 12.7 Hz, 1H, ***trans*-18a**), 3.80 (br., 1H, ***trans*-18a**), 3.57 (br., 0.45H, ***cis*-18a**), 3.22-3.17 (m, 0.45H, ***cis*-18a**), 2.77-2.64 (m, 1.45H, 1H ***trans*-18a** + 0.45H ***cis*-18a**), 2.59-2.31 (m, 2.45H, 2H ***trans*-18a** + 0.45H ***cis*-18a**), 1.85 (s, 7.35, 6H ***trans*-18a** + 1.35H ***cis*-18a**), 1.70 (s, 1.35H, ***cis*-18a**). **^{13}C NMR (75 MHz, CDCl$_3$):** δ 200.6 (trans*-18a**), 199.8 (***cis*-18a**), 145.6 (***trans*-18a**), 144.2 (***trans*-18a**), 144.1 (***cis*-18a**), 143.1 (***cis*-18a**), 133.8 (***cis*-18a**), 133.1 (***trans*-18a**), 113.8 (***cis*-18a**), 113.6 (***trans*-18a**), 74.7 (***cis*-18a**), 74.4 (***trans*-18a**), 51.1 (***trans*-18a**), 47.0 (***cis*-18a**), 30.7 (***trans*-18a**), 29.8 (***cis*-18a**), 23.1 (***cis*-18a**), 18.8 (***trans*-18a**), 15.4 (***trans*-18a**), 15.3 (***cis*-18a**). **IR (ATR, cm^{-1}):** 3477, 3076, 2922, 2856, 1724, 1645, 1435, 1136, 1032, 893. **GC/MS (*m/z*, %), *trans*-18a:** 148 (23.5), 137 (13.8), 120 (15.0), 105 (8.8), 84 (36.8), 82 (100), 67 (9.8), 54 (36.8), 41 (15.1). **GC/MS (*m/z*,**

%), ***cis*-18a:** 148 (8.8), 137 (11.3), 120 (11.0), 109 (14.8), 84 (26.3), 82 (100), 67 (14.1), 54 (45.2), 41 (21.3). Characterization data are in accordance with the literature.[63]

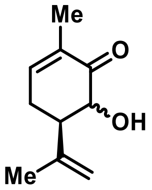

18b

trans*- and *cis*-(5*R*)-6-Hydroxy-2-methyl-5-(prop-1-en-2-yl)cyclohex-2-enone (*trans*-18b and *cis*-18b) (CAS Numbers: 423764-67-4, *trans*-18b; 1309581-71-2, *cis*-18b):** Yield: 1.43 g, 44% (three steps); *trans*:*cis* ratio = ~2:1 (GC/MS and ^{1}H NMR); yellowish oil; R_f = 0.36 and 0.27 (*trans* and *cis*, respectively) [hexane/ethyl acetate (8:2)]. **^{1}H NMR (300 MHz, CDCl$_3$):** δ 6.77-6.75 (m, 1H, ***trans*-18b**), 6.68-6.65 (m, 0.45H, ***cis*-18b**), 4.96-4.93 (m, 2H, ***trans*-18b**), 4.87-4.85 (m, 0.45H, ***cis*-18b**), 4.71 (s, 0.45H, ***cis*-18b**), 4.43 (dd, *J* = 6.0 Hz, 2.3 Hz, 0.45H, ***cis*-18b**), 4.14 (dd, *J* = 11.8 Hz, 1.6 Hz, 1H, ***trans*-18b**), 3.80 (d, *J* = 1.6 Hz, 1H, ***trans*-18b**), 3.59 (d, *J* = 2.4 Hz, 0.45H, ***cis*-18b**), 3.22-3.17 (m, 0.45H, ***cis*-18b**), 2.75-2.66 (m, 1.35H, 1H ***trans*-18b** + 0.35H ***cis*-18b**), 2.62-2.29 (m, 2.45H, 2H ***trans*-18b** + 0.45H ***cis*-18b**), 1.85 (s, 7.35 H, 6H ***trans*-18b** + 1.35H ***cis*-18b**), 1.69 (s, 1.35H, ***cis*-18b**). **^{13}C NMR (75 MHz, CDCl$_3$):** δ 200.6 (trans*-18b**), 199.8 (***cis*-18b**), 145.7 (***trans*-18b**), 144.2 (***trans*-18b**), 144.1 (***cis*-18b**), 143.1 (***cis*-18b**), 133.8 (***cis*-18b**), 133.1 (***trans*-18b**), 113.8 (***cis*-18b**), 113.6 (***trans*-18b**), 74.7 (***cis*-18b**), 74.4 (***trans*-18b**), 51.1 (***trans*-18b**), 47.0 (***cis*-18b**), 30.7 (***trans*-18b**), 29.8 (***cis*-18b**), 23.1 (***cis*-18b**), 18.8 (***trans*-18b**), 15.4 (***trans*-18b**), 15.3 (***cis*-18b**). **IR (ATR, cm^{-1}):** 3475, 2922, 1670, 1645, 1435, 1136, 1031, 891. **GC/MS (*m/z*, %), *trans*-18b:** 166 (0.7), 148 (24.4), 137 (14.5), 120 (15.5), 109 (8.6), 84 (35.5), 82 (100), 67 (9.7), 54 (37.6), 41 (15.9). **GC/MS (*m/z*, %), *cis*-18b:** 166 (2.6), 148 (8.8), 137 (12.7), 120 (11.7), 109 (15.5), 84 (26.6), 82 (100), 67 (12.8), 54 (43.2), 41 (19.1). Characterization data are in accordance with the literature.[37]

Preparation of *p*-tosylhydrazide (19)[64]

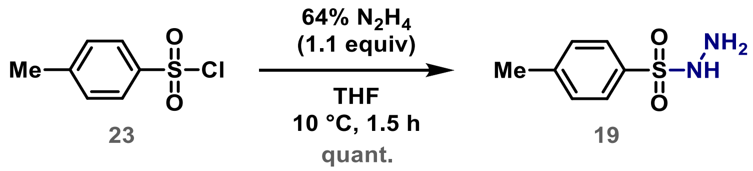

THF (4 mL) was added to a round-bottomed flask (50 mL) containing *p*-toluenesulfonyl chloride (**23**) (1.91 g, 10 mmol). The round-bottomed flask was cooled to 10 °C under

magnetic stirring and a 64% (w/v) hydrazine monohydrate solution (1.06 g, 11 mmol, 1.1 equiv) diluted in water (0.4 mL) was added dropwise every 30 s for about 20 min. The reaction mixture was magnetically stirred at room temperature for 1.5 h. Ethyl acetate (10 mL) and water (10 mL) were then added to the mixture. The aqueous phase was extracted with ethyl acetate (3 x 20 mL). The organic phase was dried over $MgSO_4$. After filtration, the solvent was evaporated under reduced pressure. *p*-Tosylhydrazide (**19**) was used without further purification.

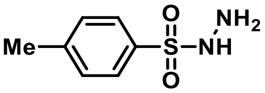

19

***p*-Tosylhydrazide (19) (CAS Number: 1576-35-8):** Yield: 1.84 g, quantitative; off-white solid; R_f = 0.73 (ethyl acetate); m.p. = 108-111 °C (lit.[65] m.p. = 108-110 °C). **^{1}H NMR (300 MHz, $CDCl_3$):** *δ* 7.80 (d, *J* = 8.1 Hz, 2H), 7.36 (d, *J* = 8.1 Hz, 2H), 5.78 (s, 1H), 3.61 (br., 2H), 2.45 (s, 3H). **^{13}C NMR (75 MHz, $CDCl_3$):** *δ* 144.6, 133.1, 129.9, 128.2, 21.6. **IR (KBr, cm^{-1}):** 3385, 3256, 1915, 1649, 1597, 1305, 1155, 837, 469. Characterization data are in accordance with the literature.[64,65]

Preparation of *trans*- and *cis*-*N'*-((5*S*)-6-hydroxy-2-methyl-5-(prop-1-en-2-yl)cyclohex-2-en-1-ylidene)-4-methylbenzenesulfonohydrazide (*trans*-20a and *cis*-20a) and *trans*- and *cis*-*N'*-((5*R*)-6-hydroxy-2-methyl-5-(prop-1-en-2-yl)cyclohex-2-en-1-ylidene)-4-methylbenzenesulfonohydrazide (*trans*-20b and *cis*-20b)[37]

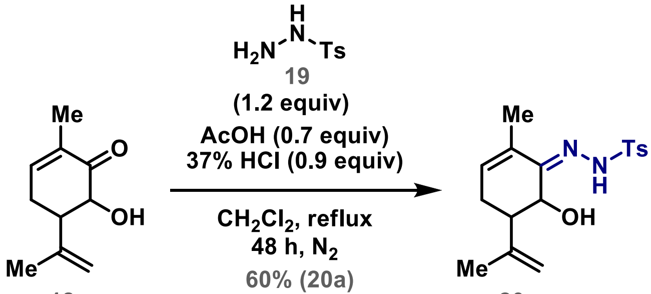

18 20

p-Tosylhydrazide (**19**) (1.12 g, 6 mmol, 1.2 equiv), acetic acid (200 µL, 3.5 mmol, 0.7 equiv), hydrochloric acid 37% (110 µL, 4.5 mmol, 0.9 equiv), and dichloromethane (75 mL) were added to a round-bottomed flask (125 mL) containing the appropriate diastereoisomeric mixture of α-hydroxycarvone **18** (830 mg, 5 mmol). The reaction mixture was magnetically stirred under reflux for 48 h. Afterwards, the mixture was washed with a 1.0 mol.L^{-1} solution of HCl (50 mL) and the aqueous phase was extracted with dichloromethane (3 x 50 mL).

The organic phase was washed with a saturated solution of $NaHCO_3$ (50 mL) and with brine (50 mL) and then dried over $MgSO_4$. After filtration, the solvent was evaporated under reduced pressure. The material obtained was purified by column chromatography on silica gel 60 using hexane/ethyl acetate (85:15 to 70:30) as eluent to afford a diastereoisomeric mixture containing ***trans*-20a** and ***cis*-20a** or ***trans*-20b** and ***cis*-20b**.

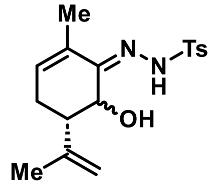

20a

trans*- and *cis*-*N'*-((5*S*)-6-Hydroxy-2-methyl-5-(prop-1-en-2-yl)cyclohex-2-en-1-ylidene)-4-methylbenzenesulfonohydrazide (*trans*-20a and *cis*-20a) (CAS Numbers: 499781-54-3, *trans*-20a; 499781-55-4, *cis*-20a):** Yield: 1.04 g, 60%; *trans*:*cis* ratio = ~3.4:1 (^{1}H NMR); orange oil; R_f = 0.53 and 0.45 (*trans* and *cis*, respectively) [hexane/ethyl acetate (67:33)]. **^{1}H NMR (300 MHz, $CDCl_3$):** δ 10.31 (br., 1H, ***trans*-20a**), 8.44 (br., 0.29H, ***cis*-20a**), 7.86-7.82 (m, 2.58H, 2H ***trans*-20a** + 0.58H ***cis*-20a**), 7.31-7.26 (m, 2.58H, 2H ***trans*-20a** + 0.58H ***cis*-20a**), 6.10 (d, J = 6.2 Hz, 0.29H, ***cis*-20a**), 5.99-5.96 (m, 1H, ***trans*-20a**), 5.11 (s, 0.29H, ***cis*-20a**), 5.04-5.00 (m, 1H, ***trans*-20a**), 4.91 (s, 1H, ***trans*-20a**), 4.86 (s, 0.29H, ***cis*-20a**), 4.65 (d, J = 2.7 Hz, 0.29H, ***cis*-20a**), 4.45 (d, J = 11.1 Hz, 1H, ***trans*-20a**), 2.57-2.37 (m, 5.74H, 4H ***trans*-20a** + 1.74H ***cis*-20a**), 2.18-2.13 (m, 2H, ***trans*-20a**), 1.82 (s, 0.87H, ***cis*-20a**), 1.79 (s, 3.87H, 3H ***trans*-20a** + 0.87H ***cis*-20a**), 1.72 (s, 3H, ***trans*-20a**). **^{13}C NMR (75 MHz, $CDCl_3$):** δ 153.5 (cis*-20a**), 149.0 (***trans*-20a**), 144.0 (***cis*-20a**), 143.5 (***trans*-20a**), 143.4 (***trans*-20a**), 143.3 (***cis*-20a**), 135.8 (***trans*-20a**), 135.2 (***cis*-20a**), 133.8 (***cis*-20a**), 133.2 (***trans*-20a**), 131.3 (***trans*-20a**), 130.8 (***cis*-20a**), 129.4 (***trans*-20a**), 129.3 (***cis*-20a**), 128.2 (***cis*-20a**), 128.0 (***trans*-20a**), 115.7 (***trans*-20a**), 114.1 (***cis*-20a**), 70.4 (***trans*-20a**), 62.4 (***cis*-20a**), 51.2 (***trans*-20a**), 45.6 (***cis*-20a**), 28.1 (***trans*-20a**), 23.8 (***cis*-20a**), 22.5 (***cis*-20a**), 21.6 (***trans*-20a** e ***cis*-20a**), 18.6 (***trans*-20a**), 18.0 (***trans*-20a**), 17.3 (***cis*-20a**). **IR (KBr, cm^{-1}):** 3472, 3311, 2920, 2843, 1645, 1597, 1167, 1028. **GC/MS (*m/z*, %):** 150 (18.9), 135 (12.8), 122 (14.1), 107 (33.4), 91 (36.2), 82 (100), 67 (14.5), 54 (18.5), 41 (11.2). Characterization data are in accordance with the literature.[37]

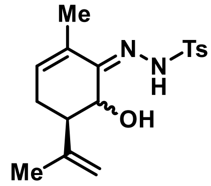

20b

trans*- and *cis-N'*-((5*R*)-6-hydroxy-2-methyl-5-(prop-1-en-2-yl)cyclohex-2-en-1-ylidene)-4-methylbenzenesulfonohydrazide (*trans*-20a and *cis*-20a) (CAS Numbers: 499781-54-3, *trans*-20b; 499781-55-4, *cis*-20b):** Yield: 965 mg, 56%; *trans*:*cis* ratio = ~3:1 (^{1}H NMR); orange oil; R_f = 0.51 and 0.44 (*trans* and *cis*, respectively) [hexane/ethyl acetate (67:33)]. **^{1}H NMR (300 MHz, $CDCl_3$):** *δ* 10.35 (s, 1H, ***trans*-20b**), 8.49 (s, 0.30H, ***cis*-20b**), 7.86-7.82 (m, 2.6H, 2H ***trans*-20b** + 0.6H ***cis*-20b**), 7.30-7.26 (m, 2.60H, 2H, ***trans*-20b** + 0.60 ***cis*-20b**), 6.10 (d, *J* = 6.2 Hz, 0.30H, ***cis*-20b**), 5.97 (br., 1H, ***trans*-20b**), 5.10 (s, 0.30H, ***cis*-20b**), 5.01 (s, 1H, ***trans*-20b**), 4.90 (s, 1H, ***trans*-20b**), 4.85 (s, 0.30H, ***cis*-20b**), 4.65 (br., 0.30H, ***cis*-20b**), 4.45 (d, *J* = 11.1 Hz, 1H, ***trans*-20b**), 2.54-2.40 (m, 5.80H, 4H ***trans*-20b** + 1.80H ***cis*-20b**), 2.18-2.12 (m, 2H, ***trans*-20b**), 1.81 (s, 0.90H, ***cis*-20b**), 1.78 (s, 3.90H, 2H , ***trans*-20b** + 0.90H ***cis*-20b**), 1.71 (s, 3H, ***trans*-20b**). **^{13}C NMR (75 MHz, $CDCl_3$):** *δ* 153.6 (cis*-20b**), 149.2 (***trans*-20b**), 144.0 (***cis*-20b**), 143.6 (***trans*-20b**), 143.4 (***trans*-20b**), 143.3 (***cis*-20b**), 135.8 (***trans*-20b**), 135.2 (***cis*-20b**), 133.9 (***cis*-20b**), 133.1 (***trans*-20b**), 131.3 (***trans*-20b**), 130.9 (***cis*-20b**), 129.4 (***trans*-20b**), 129.3 (***cis*-20b**), 128.2 (***cis*-20b**), 128.0 (***trans*-20b**), 115.6 (***trans*-20b**), 114.1 (***cis*-20b**), 70.5 (***trans*-20b**), 62.5 (***cis*-20b**), 51.1 (***trans*-20b**), 45.7 (***cis*-20b**), 28.2 (**24a**), 23.9 (***cis*-20b**), 22.5 (***cis*-20b**), 21.6 (***trans*-20b** and ***cis*-20b**), 18.7 (***trans*-20b**), 18.1 (***trans*-20b**), 17.4 (***cis*-20b**). **IR (KBr, cm^{-1}):** 3472, 3311, 2920, 1645, 1597, 1166, 1028. **GC/MS (*m/z*, %):** 150 (15.4), 135 (9.7), 122 (12.9), 107 (30.2), 91 (35.5), 82 (100), 67 (13.4), 54 (20.3), 41 (11.8). Characterization data are in accordance with the literature.[37]

Preparation of *trans*- and *cis*-(6*S*)-isopiperitenol (*trans*-9a and *cis*-9a) and *trans*- and *cis*-(6*R*)-isopiperitenol (*trans*-9b and *cis*-9b)[37]

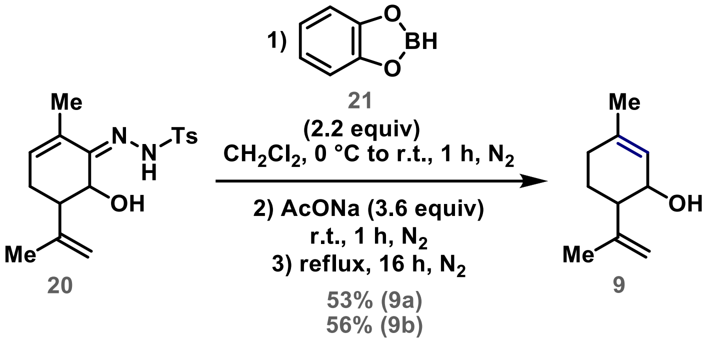

Dry dichloromethane (50 mL) was added to a flame dried round-bottom flask (125 mL) containing the appropriate diastereoisomeric mixture of hydrazone **20** (835 mg, 2.5 mmol). The round-bottomed flask was cooled to 0 °C under magnetic stirring and a nitrogen atmosphere. Catecholborane (**21**) (5.5 mL of a 1.0 mol.L^{-1} solution in THF, 5.5 mmol, 2.2 equiv) was added dropwise and the mixture was heated to room temperature. After 1 h, anhydrous sodium acetate (1.23 g, 9.0 mmol, 3.6 equiv) was added rapidly and the mixture was maintained at room temperature. After 1 h, the reaction was heated to reflux under magnetic stirring and a nitrogen atmosphere for 16 h. The reaction mixture was then washed with a saturated solution of $NaHCO_3$ (50 mL), brine (50 mL), and water (50 mL). The aqueous phase was extracted with dichloromethane (2 x 50 mL). The organic phase was dried over Na_2SO_4. After filtration, the solvent was evaporated under reduced pressure. The material obtained was purified by column chromatography on silica gel 60 using hexane/ethyl acetate (67:33) as eluent to give a diastereoisomeric mixture containing ***trans*-9a** and ***cis*-9a** or ***trans*-9b** and ***cis*-9b**.

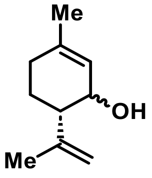

9a

***trans*- and *cis*-(6*S*)-Isopiperitenol (*trans*-9a and *cis*-9a) (CAS Numbers: 4017-77-0, *trans*-9a; 4017-76-9, *cis*-9a):** Yield: 201 mg, 53%; *trans*:*cis* ratio = ~8:1 (GC/MS and ^{1}H NMR); yellowish oil; R_f = 0.52 (*trans* and *cis*) [hexane/ethyl acetate (67:33)]. **^{1}H NMR (300 MHz, $CDCl_3$):** δ 5.70-5.66 (m, 0.13H, ***cis*-9a**), 5.45 (s, 1H, ***trans*-9a**), 5.00 (s, 0.13H, ***cis*-9a**), 4.90-4.88 (m, 1H, ***trans*-9a**), 4.85 (s, 1H, ***trans*-9a**), 4.81 (s, 0.13H, ***cis*-9a**), 4.14-4.09 (m, 1.13H, 1H ***trans*-9a** + 0.13H ***cis*-9a**), 2.14-2.00 (m, 2.39H, 2H ***trans*-9a** + 0.39H ***cis*-9a**), 1.97-1.87 (m, 1H, ***trans*-9a**), 1,83 (s, 0.39H, ***cis*-9a**), 1.76-1.51 (m, 8.65H, 8H ***trans*-9a** +

0.65H ***cis*-9a**). **^{13}C NMR (75 MHz, $CDCl_3$):** δ 146.5 (***cis*-9a**), 146.4 (***trans*-9a**), 139.7 (***cis*-9a**), 136.7 (***trans*-9a**), 124.3 (***trans*-9a**), 122.4 (***cis*-9a**), 112.3 (***trans*-9a**), 111.7 (***cis*-9a**), 68.7 (***trans*-9a**), 63.8 (***cis*-9a**), 50.9 (***trans*-9a**), 46.1 (***cis*-9a**), 31.1 (***cis*-9a**), 30.2 (***trans*-9a**), 26.2 (***trans*-9a**), 23.4 (***cis*-9a**), 23.0 (***trans*-9a**), 22.6 (***cis*-9a**), 20.8 (***cis*-9a**), 19.3 (***trans*-9a**). **IR (ATR, cm^{-1}):** 3363, 2964, 2922, 2833, 1674, 1643, 1438, 1375, 1151, 1029. **GC/MS(*m/z*, %), *trans*-9a:** 134 (62.9), 119 (80.1), 105 (26.7), 91 (100), 77 (27.0), 65 (13.5), 41 (19.0). **GC/MS (*m/z*, %), *cis*-9a:** 134 (49.3), 119 (65.8), 105 (23.9), 91 (100.0), 77 (27.9), 65 (15.0), 41 (19.2). Characterization data are in accordance with the literature.[66]

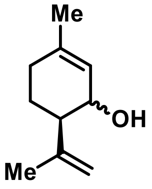

9b

trans*- and *cis*-(6*R*)-Isopiperitenol (*trans*-9b and *cis*-9b) (CAS Numbers: 74410-00-7, *trans*-9b; 96555-02-1, *cis*-9b):** Yield: 212 mg, 56%; *trans:cis* ratio = ~6:1 (GC/MS and ^{1}H NMR); yellowish oil; R_f = 0.52 (*trans* and *cis*) [hexane/ethyl acetate (67:33)]. **^{1}H NMR (300 MHz, $CDCl_3$):** δ 5.70-5.66 (m, 0.19H, ***cis*-9b**), 5.44 (s, 1H, ***trans*-9b**), 5.00 (s, 0.19H, ***cis*-9b**), 4.90-4.86 (m, 1H, ***trans*-9b**), 4.85 (s, 1H, ***trans*-9b**), 4.81 (s, 0.19H, ***cis*-9b**), 4.15-4.08 (m, 1.19H, 1H ***trans*-9b** + 0.19H ***cis*-9b**), 2.13-2.02 (m, 2.57H, 2H ***trans*-9b** + 0.57H ***cis*-9b**), 1.98-1.96 (m, 1H, ***trans*-9b**), 1.83 (s, 0.57H, ***cis*-9b**), 1.73-1.61 (m, 8.95H, 8H ***trans*-9b** + 0.95H ***cis*-9b**). **^{13}C NMR (75 MHz, $CDCl_3$):** δ 146.7 (cis*-9b**), 146.6 (***trans*-9b**), 139.9 (***cis*-9b**), 136.9 (***trans*-9b**), 124.4 (***trans*-9b**), 122.5 (***cis*-9b**), 112.5 (***trans*-9b**), 111.8 (***cis*-9b**), 68.8 (***trans*-9b**), 63.9 (***cis*-9b**), 51.1 (***trans*-9b**), 46.2 (***cis*-9b**), 31.3 (***cis*-9b**), 30.3 (***trans*-9b**), 26.3 (***trans*-9b**), 23.5 (***cis*-9b**), 23.2 (***trans*-9b**), 22.7 (***cis*-9b**), 21.0 (***cis*-9b**), 19.4 (***trans*-9b**). **IR (RTA, cm^{-1}):** 3367, 2926, 1641, 1438, 1375, 1151, 1031. **GC/MS (*m/z*, %), *trans*-9b:** 134 (62.8), 119 (80.2), 105 (26.8), 91 (100), 77 (27.1), 65 (13.6), 41 (19.3). **GC/MS (*m/z*, %), *cis*-9b:** 134 (49.4), 119 (65.9), 105 (23.8), 91 (100.0), 77 (27.8), 65 (15.2), 41 (19.0). Characterization data are in accordance with the literature.[37]

Preparation of cyclohex-2-enol (24)[67]

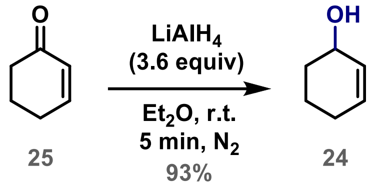

Diethyl ether (50 mL) was added to a round-bottomed flask (125 mL) containing $LiAlH_4$ (1.37 g, 36 mmol, 3.6 equiv) and magnetic stirring was started at room temperature under a nitrogen atmosphere. A solution of cyclohex-2-enone (**25**) (960 mg, 10 mmol) in diethyl ether (70 mL) was slowly added. The reaction mixture was magnetically stirred under reflux and a nitrogen atmosphere for 5 min. Afterwards, the mixture was quenched with water (20 mL) and neutralized with a 10% solution of HCl. The aqueous phase was extracted with diethyl ether Et_2O (3 x 50 mL) and washed with brine (100 mL). The organic phase was dried over $MgSO_4$. After filtration, the solvent was evaporated under reduced pressure. Cyclohex-2-enol (**24**) was used without further purification.

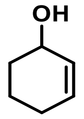

24

Cyclohex-2-enol (24) (CAS Number: 822-67-3): Yield: 910 mg, 93%; colorless oil; R_f = 0.31 [hexanes/ethyl acetate (7:3)]. **^{1}H NMR (300 MHz, $CDCl_3$):** δ 5.87-5.80 (m, 1H), 5.78-5.72 (m., 1H), 4.20 (br., 1H), 2.03-1.84 (m, 3H), 1.73-1.58 (m, 4H). **^{13}C NMR (75 MHz, $CDCl_3$):** δ 130.5, 129.8, 65.4, 31.9, 25.0, 18.9. **IR (ATR, cm^{-1}):** 3329, 2932, 2860, 2835, 1651, 1450, 1435, 1265, 1001. **GC/MS (*m/z*, %):** 98 (40.6), 97(44.7), 70 (100), 55 (28.1). Characterization data are in accordance with the literature.[67]

Table S2. Optimization of Friedel-Crafts reaction conditions between olivetol (**8**) and cyclohex-2-enol (**24**).[a]

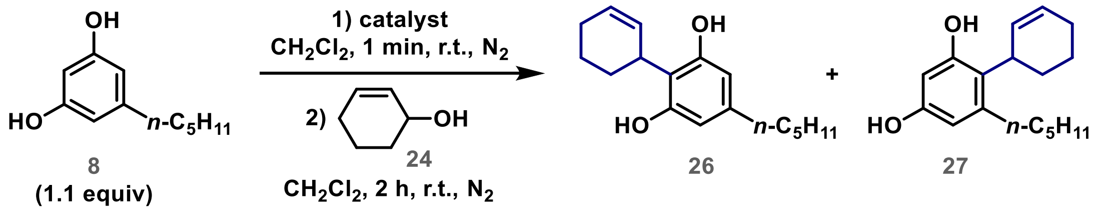

Entry	Catalyst (mol%)	NMR yield of **26** (%)[b]	NMR yield of **27** (%)[b]	Isolated yield of **26** (%)	Isolated yield of **27** (%)
1[43]	TMSOTf (20)[c]	42	38	32	26
2 [19]	AgOTf (20)[d,e]	38	34	26	19
3[43]	$Cu(OTf)_2$ (20)[d]	7	7	<5	<5
4[43]	$Sc(OTf)_3$ (20)[d]	0	0	0	0
5[43]	$AlCl_3$ (20)	14	14	<5	<5
6[68]	$FeCl_3 \cdot 6H_2O$ (20)[c]	31	29	19[f]	13
7[43]	$SnCl_4$ (20)[c]	24	20	13	6
8[43]	MsOH (20)[d]	42	33	30	19
9[43]	$TsOH.H_2O$ (20)[d]	0	0	0	0
10[69]	CSA (20)	40	29	33	18
11[69]	CSA (10)	44	38	35	22
12[69]	CSA (5)	32	30	26	16

[a] Reaction conditions: cyclohex-2-enol (**24**) (0.3 mmol), 1.1 equiv of olivetol (**8**), and the catalyst (5-20 mol%) were stirred at room temperature for 2 hours under N_2 atmosphere. [b] NMR yield calculated using 1,3-dinitrobenzene as internal standard (see **Table S4** for more details). [c] A by-product of *m/z* 340 was formed (GC/MS). [d] The starting alcohol **24** was partially recovered (GC/MS and ^{1}H NMR). [e] The reaction was protected from light with aluminum foil. [f] It presented a laborious purification by chromatography.

Preparation of 2-(cyclohex-2-enyl)-5-pentylbenzene-1,3-diol (26) and 4-(cyclohex-2-enyl)-5-pentylbenzene-1,3-diol (27)[69]

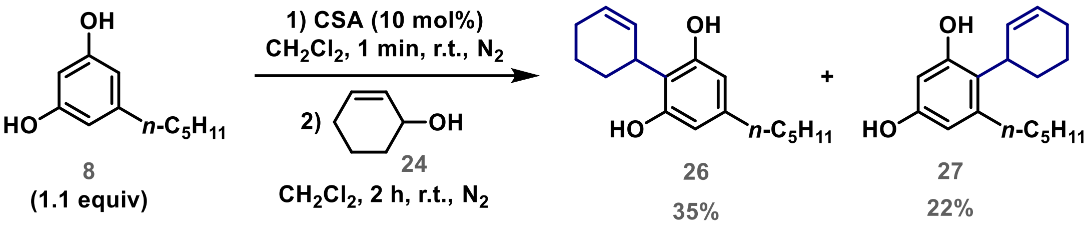

CSA (7.0 mg, 0.03 mmol, 10 mol%) was added to a round-bottomed flask (10 mL), which was quickly sealed with a rubber septum and maintained under a nitrogen atmosphere. A solution of olivetol (**8**) (58.8 mg, 0.33 mmol, 1.1 equiv) in dichloromethane (2.5 mL) was added and magnetic stirring was started. After 1 min, a solution of cyclohex-2-enol (**24**) (29.4 mg, 0.3 mmol) in dichloromethane (2.5 mL) was added dropwise. The reaction mixture was kept at room temperature under magnetic stirring and a nitrogen atmosphere for 2 h. A saturated solution of $NaHCO_3$ (5 mL) was then added to the reaction. The aqueous phase was extracted with dichloromethane (3 x 5 mL). The organic phase was washed with brine (5 mL) and dried over Na_2SO_4. After filtration, the solvent was removed under reduced pressure. The residue was purified by column chromatography on silica gel 60 using hexane/ethyl acetate (85:15) as eluent to afford 2-(cyclohex-2-enyl)-5-pentylbenzene-1,3-diol (**26**) and 4-(cyclohex-2-enyl)-5-pentylbenzene-1,3-diol (**27**).

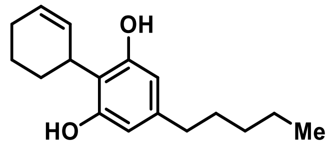

26

2-(Cyclohex-2-enyl)-5-pentylbenzene-1,3-diol (26) (CAS Number: 117824-40-5): Yield: 27.2 mg, 35%; yellowish oil; R_f = 0.61 [hexane/ethyl acetate (7:3)]. **^{1}H NMR (300 MHz, $CDCl_3$):** δ 6.22 (br., 2H), 6.19-6.12 (m, 1H), 5.95-5.90 (m, 1H), 5.53 (br., 2H), 3.98-3.91 (m, 1H), 2.44 (t, J = 7.8 Hz, 2H), 2.17-2.14 (m, 2H), 2.02-1.95 (m, 1H), 1.90-1.86 (m, 1H), 1.72-1.66 (m, 2H), 1.59-1.53 (m, 2H), 1.33-1.28 (m, 4H), 0.88 (t, J = 6.9 Hz, 3H). **^{13}C NMR (75 MHz, $CDCl_3$):** δ 154.9, 143.0, 132.9, 130.3, 114.1, 108.5, 35.5, 32.0, 31.5, 30.7, 28.6, 25.0, 22.5, 22.0, 14.0. **IR (KBr, cm^{-1}):** 3446, 2927, 2856, 1627, 1581, 1445, 1340, 1217, 1049. **GC/MS (*m/z*, %):** 260 (42.0), 217 (29.3), 204 (100), 193 (7.5), 161 (17.3), 136 (26.2), 123 (11.7), 81 (10.8). Characterization data are in accordance with the literature.[70]

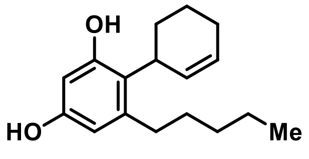

27

4-(Cyclohex-2-enyl)-5-pentylbenzene-1,3-diol (**27**) **(CAS Number: 151705-45-2)**: Yield: 17.6 mg, 22%; yellowish oil; R_f = 0.40 [hexane/ethyl acetate (7:3)]. **^{1}H NMR (300 MHz, $CDCl_3$):** *δ* 6.25-6.20 (dd, *J* = 7.1, 2.6 Hz, 2H), 6.17-6.10 (m, 1H), 6.04 (br., 1H), 5.87 (d, *J* = 9.9 Hz, 1H), 4,93 (br., 1H), 3.64 (br., 1H), 2.58-2.44 (m, 2H), 2.16 (br., 2H), 1.95-1.87 (m, 2H), 1.78-1.66 (m, 2H), 1.55-1.47 (m, 2H), 1.37-1.31 (m, 4H), 0.90 (t, *J* = 6.9 Hz, 3H).**^{13}C NMR (75 MHz, $CDCl_3$):** *δ* 156.7, 154.6, 142.9, 132.6, 130.7, 120.4, 108.9, 102.1, 35.0, 33.9, 31.8, 31.3, 28.7, 24.8, 22.5, 22.5, 14.0. **IR (KBr, cm^{-1}):** 3447, 3017, 2929, 2857, 1620, 1593, 1450, 1377, 1142. **GC/MS (*m/z*, %):** 260 (81.1), 232 (10.4), 217 (50.3), 204 (57.5) 189 (46.9), 175 (68.2), 161 (100), 147 (23.0), 81 (19.9). Characterization data are in accordance with the literature.[70]

Preparation of (-)-cannabidiol (CBD, 1) and (-)-abnormal cannabidiol (*abn*-CBD, 13) and (+)-cannabidiol (*ent*-CBD, *ent*-1) and (+)-abnormal cannabidiol (*ent*-*abn*-CBD, *ent*-13)[69]

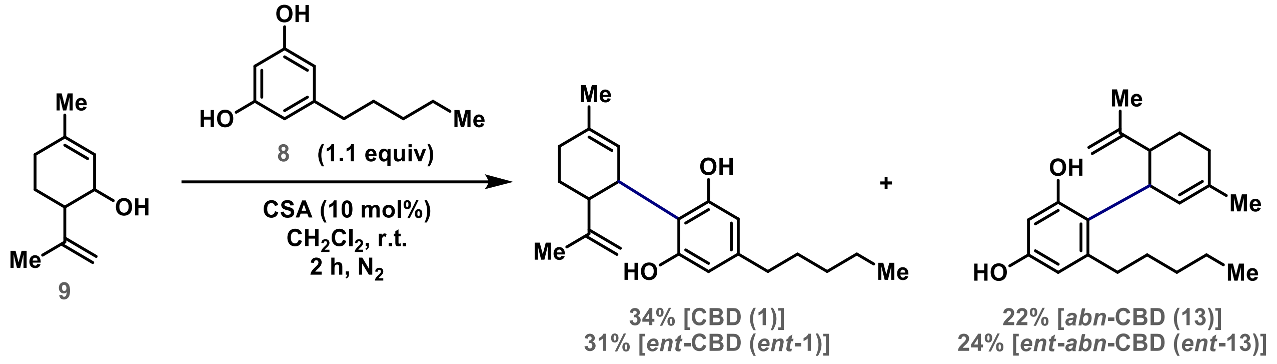

CSA (23.2 mg, 0.1 mmol, 10 mol%) was added to a round-bottomed flask (25 mL), which was quickly sealed with a rubber septum and maintained under a nitrogen atmosphere. A solution of olivetol (**8**) (196 mg, 1.1 mmol, 1.1 equiv) in dichloromethane (7.5 mL) was added and magnetic stirring was started. After 1 min, a solution of the appropriate *trans*:*cis* mixture of isopiperitenol (**9**) (152 mg, 1 mmol) in dichloromethane (7.5 mL) was added dropwise. The reaction mixture was kept at room temperature under magnetic stirring and a nitrogen atmosphere for 2 h. Afterwards, a saturated solution of $NaHCO_3$ (15 mL) was added to the reaction. The aqueous phase was extracted with dichloromethane (3 x 15 mL). The organic phase was washed with brine (15 mL) and dried over Na_2SO_4. After filtration, the solvent

was removed under reduced pressure. The material was purified by column chromatography on silica gel 60 using hexane/ethyl acetate (85:15) as eluent to give CBD (**1**) and *abn*-CBD (**13**) or *ent*-CBD (*ent*-**1**) and *ent*-*abn*-CBD (*ent*-**13**).

NOTE: When the reaction was carried out with AgOTf, after adding the catalyst (51.4 mg, 0.2 mmol, 20 mol%) to the round-bottomed flask (25 mL), the flask was quickly sealed with a rubber septum, protected from light with aluminum foil, and maintained under a nitrogen atmosphere. The procedure described for CSA was then followed.

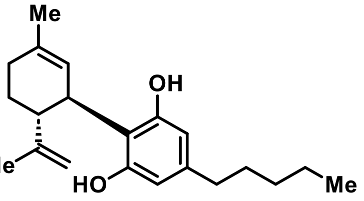

CBD (1)

(-)-Cannabidiol (CBD, 1) (CAS Number: 13956-29-1): Yield: 106.8 mg, 34% (CSA), 94.6 mg, 30% (AgOTf); yellowish oil; R_f = 0.53 [hexane/ethyl acetate (85:15)]; $[\alpha]_D^{22}$ = -124.4 (*c* = 1.0, EtOH) (CSA), $[\alpha]_D^{23}$ = -121.3 (*c* = 1.0, EtOH) (AgOTf) {lit.[19] $[\alpha]_D^{20}$ = -124 (*c* = 1.0, EtOH)}. **^{1}H NMR (300 MHz, $CDCl_3$):** δ 6.32-6.13 (m, 2H), 5.97 (br., 1H), 5.57 (s, 1H), 4.80-4.72 (br., 1H), 4.67-4.63 (m, 1H), 4.56 (s, 1H), 3.90-3.82 (m, 1H), 2.46-2.35 (m, 3H), 2.28-2.05 (m, 2H), 1.85-1.74 (m, 5H), 1.66 (s, 3H), 1.60-1.50 (m, 2H), 1.33-1.25 (m, 4H), 0.88 (t, *J* = 6.6 Hz, 3H); **^{13}C NMR (75 MHz, $CDCl_3$):** δ 155.9, 154.0, 149.2, 143.0, 139.9, 124.1, 113.8, 110.8, 109.6, 108.0, 46.2, 37.1, 35.5, 31.5, 30.6, 30.4, 28.3, 23.6, 22.5, 20.3, 14.0; **IR (KBr, cm^{-1}):** 3435, 2957, 2926, 2857, 2833, 1630, 1583, 1445, 1377, 1217, 1026; **GC/MS (*m/z*, %):** 314 (9.8), 299 (4.9), 246 (11.8), 231 (100.0), 193 (7.5), 174 (9.1), 121 (7.5), 91 (4.6). Characterization data are in accordance with the literature.[19]

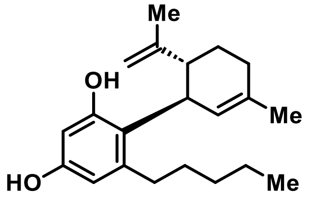

***abn*-CBD (13)**

(-)-Abnormal cannabidiol (*abn*-CBD, 13) (CAS Number: 22972-55-0): Yield: 69.0 mg, 22% (CSA), 93.9 mg, 30% (AgOTf); yellowish oil; R_f = 0.17 [hexane/ethyl acetate (85:15)]; $[\alpha]_D^{22}$ = -77.2 (*c* = 0.40, $CHCl_3$) (CSA), $[\alpha]_D^{23}$ = -72.3 (*c* = 0.40, $CHCl_3$) (AgOTf) {lit. [71] $[\alpha]_D$ = -76 ($CHCl_3$)}. **^{1}H NMR (300 MHz, $CDCl_3$):** δ 6.21-6.16 (m, 2H), 6.04 (s, 1H), 5.52 (s, 1H), 4.64 (s, 1H), 4.57 (s, 1H), 4.46 (s, 1H), 3.56-3.50 (m, 1H) , 2.62 -2.43 (m, 2H), 2.31-2.20 (m, 2H), 2.12-2.05 (m, 1H) 1.84-1.74 (m, 5H), 1.53 (s, 3H), 1.49-1.42 (m, 2H), 1.32-1.24 (m,

4H), 0.88 (t, J = 6.8 Hz, 3H). **^{13}C NMR (75 MHz, $CDCl_3$):** δ 156.5, 154.5, 147.7, 144.0, 139.8, 124.7, 119.9, 111.4, 108.5, 102.1, 44.9, 40.0, 34.0, 31.9, 31.1, 30.2, 28.1, 23.6, 22.5, 21.3, 14.0. **IR (KBr, cm^{-1}):** 3427, 2957, 2926, 2857, 2833, 1620, 1593, 1449, 1375, 1265, 1150. **GC/MS (*m/z*, %):** 314 (29.8), 299 (5.1), 246 (45.6), 231 (100.0), 203 (45.0), 189 (59.4), 175 (90.8), 161 (32.9) 147 (16.4). Characterization data are in accordance with the literature.[72]

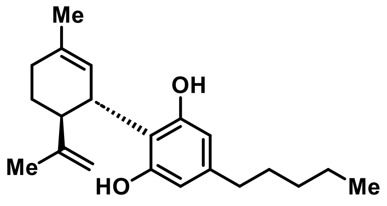

***ent*-CBD (*ent*-1)**

(+)-Cannabidiol (*ent*-CBD, *ent*-1) (CAS Number: 74219-29-7): Yield: 97.4 mg, 31% (CSA), 72.5 mg, 23% (AgOTf); yellowish oil; R_f = 0.53 [hexane/ethyl acetate (85:15)]; $[\alpha]_D^{22}$ = +121.0 (c = 1.0, EtOH) (CSA), $[\alpha]_D^{23}$ = +125.2 (c = 1.0, EtOH) (AgOTf) {lit.[37] $[\alpha]_D^{20}$ = +124 (c = 1.0, EtOH)}. **^{1}H NMR (300 MHz, $CDCl_3$):** δ 6.32-6.13 (m, 2H), 5.97 (br., 1H), 5.56 (s, 1H), 4.68-4.65 (m, 1H), 4.55 (s, 1H), 3.89-3.82 (m, 1H), 2.46-2.35 (m, 3H), 2.24-2.04 (m, 2H), 1.86-1.74 (m, 5H), 1.65 (s, 3H), 1.60-1.49 (m, 2H), 1.35-1.25 (m, 4H), 0.88 (t, J = 6.6 Hz, 3H). **^{13}C NMR (75 MHz, $CDCl_3$):** δ 156.0, 153.9, 149.3, 143.0, 140.0, 124.0, 113.7, 110.8, 109.7, 108.0, 46.0, 37.2, 35.4, 31.4, 30.6, 30.3, 28.3, 23.6, 22.5, 20.4, 14.0. **IR (KBr, cm^{-1}):** 3427, 2956, 2926, 2856, 2830, 1624, 1585, 1446, 1377, 1217, 1026. **GC/MS (*m/z*, %):** 314 (8.9), 299 (6.3), 246 (8.9), 231 (100.0), 193 (8.4), 174 (13.0), 121 (8.9), 91 (12.9). Characterization data are in accordance with the literature.[37]

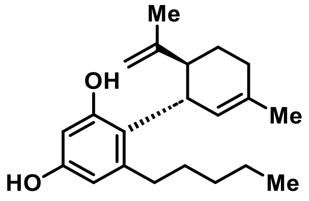

***ent-abn*-CBD (*ent*-13)**

(+)-Abnormal cannabidiol (*ent-abn*-CBD, *ent*-13) (CAS Number: 2608848-53-7): Yield: 75.4 mg, 24% (CSA), 63.0 mg, 20% (AgOTf); yellowish oil; R_f = 0.17 [hexane/ethyl acetate (85:15)]; $[\alpha]_D^{22}$ = +79.8 (c = 0.40, $CHCl_3$) (CSA), $[\alpha]_D^{23}$ = +72.3 (c = 0.40, $CHCl_3$) (AgOTf) {lit.[37] $[\alpha]_D^{20}$ = +68.8 (c = 0.40, $CHCl_3$)}. **^{1}H NMR (300 MHz, $CDCl_3$):** δ 6.21-6.16 (m, 2H), 6.04 (s, 1H), 5.52 (s, 1H), 4.64 (s, 1H), 4.58 (s, 1H), 4.45 (s, 1H), 3.56-3.49 (m, 1H), 2.63-2.43 (m, 2H), 2.30-2.18 (m, 2H), 2.12-2.04 (m, 1H), 1.84-1.74 (m, 5H), 1.53 (s, 3H), 1.49-1.41 (m, 2H), 1.35-1.23 (m, 4H), 0.89 (t, J = 6.8 Hz, 3H). **^{13}C NMR (75 MHz, $CDCl_3$):** δ 156.4, 154.6, 147.6, 144.0, 139.8, 124.7, 119.9, 111.4, 108.6, 102.1, 44.9, 40.0, 34.0, 31.9, 31.1,

30.2, 28.1, 23.6, 22.5, 21.3, 14.0. **IR (KBr, cm^{-1}):** 3419, 2956, 2926, 2857, 2833, 1620, 1593, 1446, 1375, 1265, 1150. **GC/MS (*m/z*, %):** 314 (26.4), 299 (8.1), 246 (32.8), 231 (100.0), 203 (38.9), 189 (46.6), 175 (72.1), 161 (30.3), 147 (15.7). Characterization data are in accordance with the literature.[37]

Preparation of *N*-benzylidenebenzenesulfonamide (28)[73]

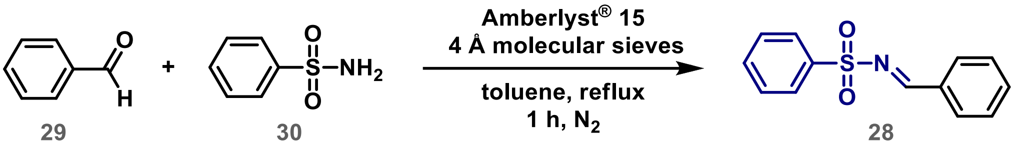

29 30 84% 28

Amberlyst® 15 hydrogen form (Dry) (100 mg), benzaldehyde (**29**) (530.6 mg, 5 mmol), benzenesulfonamide (**30**) (786.0 mg, 5 mmol, 1 equiv), and toluene (10 mL) were added to a round-bottomed flask (125 mL) containing 4Å molecular sieves (1 g). The reaction mixture was refluxed under magnetic stirring and a nitrogen atmosphere for 1 h. The reaction was then cooled to room temperature, filtered through a celite pad, and washed with toluene. The solvent was evaporated under reduced pressure and the material was maintained under refrigeration for 30 min. The solid obtained was washed with *n*-pentane and the residual solvent was removed under reduced pressure. *N*-benzylidenebenzenesulfonamide (**28**) was used without further purification.

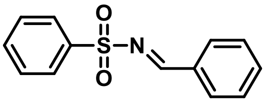

28

***N*-benzylidenebenzenesulfonamide (28) (CAS number: 13909-34-7):** Yield: 60.9 mg, 84%; off-white solid; R_f = 0.46 [hexane/ethyl acetate (67:33)]; m.p. = 73.0-75.0 °C (lit.[74] m.p. = 75-77 °C). **^{1}H NMR (300 MHz, CDCl$_3$):** δ 9.06 (s, 1H), 8.03-8.00 (m, 2H), 7.95-7.90 (m, 2H), 7.65-7.46 (m, 6H). **^{13}C NMR (75 MHz, CDCl$_3$):** δ 170.6, 138.1, 135.0, 133.5, 132.2, 131.3, 129.1, 127.9, 126.3. **IR (KBr, cm^{-1}):** 3348, 1664, 1311, 1159, 748, 682. **GC/MS (*m/z*, %):** 245 (10.7), 181 (6.4), 141 (43.5), 104 (43.5), 77 (100.0), 51 (18.4). Characterization data are in accordance with the literature.[73,74]

Preparation of 3-phenyl-2-(phenylsulfonyl)-1,2-oxaziridine (22)[46]

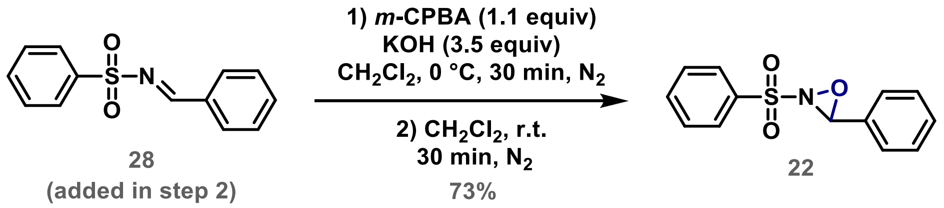

Dichloromethane (30 mL) and KOH (1.96, 3.5 mmol, 3.5 equiv) were added to a round-bottomed flask (125 mL) containing *m*-CPBA <77% (1.9 g, 1.1 mmol, 1.1 equiv). The round-bottomed flask was cooled to 0 °C under magnetic stirring and a nitrogen atmosphere. After 30 min, a solution of *N*-benzylidenebenzenesulfonamide (**28**) in dichloromethane (30 mL) was added. The reaction mixture was heated to room temperature and maintained under magnetic stirring for 30 min. Afterwards, the reaction was filtered and the solvent was removed under reduced pressure. 3-Phenyl-2-(phenylsulfonyl)-1,2-oxaziridine (**22**) was used without further purification.

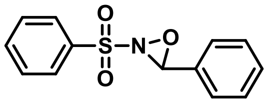

22

3-Phenyl-2-(phenylsulfonyl)-1,2-oxaziridine (22) (CAS number: 63160-13-4): Yield: 190 mg, 73%; off-white solid; R_f = 0.56 [hexane/ethyl acetate (1:1)]; m.p. = 93.0-94.0 °C (lit.[75] m.p. = 96-97 °C). **^{1}H NMR (300 MHz, $CDCl_3$):** δ 8.08-8.02 (m, 2H), 7.77-7.60 (m, 3H), 7.47-7.36 (m, 5H), 5.48 (s, 1H). **^{13}C NMR (75 MHz, $CDCl_3$):** δ 135.0, 134.7, 131.5, 130.4, 129.4, 129.4, 128.8, 128.2, 76.3. **IR (KBr, cm^{-1}):** 3064, 1350, 1168, 729, 578. **GC/MS (*m/z*, %):** 245 (5.1), 141 (31.9), 104 (36.7), 77 (100.0), 51 (21.3). Characterization data are in accordance with the literature.[75,76]

Table S3. Optimization of Davis oxidation conditions to obtain α-hydroxycarvone (**18b**).[a]

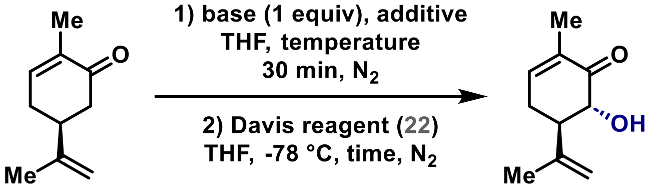

Entry	Base	Additive (equiv)	Temp. (°C)	Davis reagent **22** (equiv)	Time (min)	Isolated yield of **18b** (%)[b]
1	LDA	-	-78	2	20	0
2	*t*-BuOK	-	-78	2	20	0
3	LDA	-	-78	2	60	26[c]
4	*t*-BuOK	-	-78	2	60	0
5	KHMDS	-	-78	2	60	0
6	LiHMDS	-	-78	2	60	< 2
7	NaHMDS	-	-78	2	60	0
8	LDA	-	r.t.	2	60	37[c]
9	LDA	-	r.t.	1.5	60	38[c]
10	LDA	HMPA (3)	r.t.	1.5	60	21[c]
11	LDA	-	r.t.	1.5	180	0[d]
12	LDA	-	r.t.	1.5	60	35[c,e]

[a] Reaction conditions: (*S*)-(+)-carvone (**15b**) (1 mmol), 1 equiv of the indicated base, 3 equiv of the indicated additive, and the indicated amount of Davis reagent (**22**) in THF were stirred at the indicated temperature for the indicated time under N_2 atmosphere. [b] (*S*)-(+)-carvone (**15b**) and Davis reagente **22** were partially recovered in all reactions (GC/MS). [c] Only the *trans* diastereoisomer of **18b** was formed (GC/MS). [d] A diastereoisomeric mixture of **18b** was formed (GC/MS), which could not be isolated with satisfactory purity. [e] Reaction performed on a 5 mmol scale.

Preparation of *trans*-(5*R*,6*R*)-6-hydroxy-2-methyl-5-(prop-1-en-2-yl)cyclohex-2-enone (*trans*-18b)

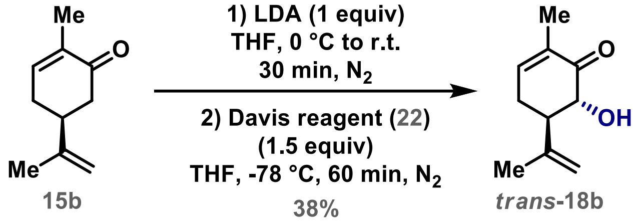

THF (25 mL) was added to a round-bottomed flask (125 mL) containing DIPA (750 µL, 5 mmol, 1 equiv). The round-bottomed flask was cooled to -78 °C under magnetic stirring and a nitrogen atmosphere. *n*-BuLi (3.0 mL of a 1.71 mol.L^{-1} solution in hexane, 5 mmol, 1 equiv) was added and the mixture was heated to 0 °C. After 1.5 h, *S*-(+)-carvone (**15b**) (750 mg, 5 mmol) was added and the reaction was heated to room temperature. After 30 min, the mixture was cooled again to -78 °C and a solution of 3-phenyl-2-(phenylsulfonyl)-1,2-oxaziridine (**22**) (1.96 g, 7.5 mmol, 1.5 equiv) in THF (50 mL) was added. The reaction was maintained at -78 °C under magnetic stirring and a nitrogen atmosphere for 60 min. A saturated solution of NH_4Cl was then added until precipitation of LiCl. The mixture was concentrated under reduced pressure, extracted with diethyl ether (3 x 50 mL), and dried over Na_2SO_4. After filtration, the solvent was evaporated under reduced pressure. The material was purified by column chromatography on silica gel 60 using *n*-hexane/ethyl acetate (8:2) as eluent to afford *trans*-(5*R,6R*)-6-hydroxy-2-methyl-5-(prop-1-en-2-yl)cyclohex-2-enone (***trans*-18b**).

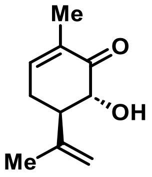

trans-18b

***trans*-(5*R,6R*)-6-Hydroxy-2-methyl-5-(prop-1-en-2-yl)cyclohex-2-enone (*trans*-18b) (CAS number: 423764-67-4)**: Yield: 63 mg, 38%; yellowish oil; R_f = 0.36 [*n*-hexane/ethyl acetate (8:2)]; $[\alpha]_D^{23}$ = +27.9 (*c* = 1.0, EtOH) {lit.[77] $[\alpha]_D^{24}$ = +28.1 (c = 0.50, CH_2Cl_2)}. **^{1}H NMR (300 MHz, $CDCl_3$):** δ 6.76-6.74 (m, 1H), 4.95-4.94 (m, 2H), 4.16 (d, *J* = 12.7 Hz, 1H), 3.79 (br., 1H), 2.75-2.65 (m, 1H), 2.54-2.32 (m, 2H), 1.84 (s, 6H). **^{13}C NMR (75 MHz, $CDCl_3$):** δ 200.6, 145.7, 144.2, 133.1, 113.6, 74.4, 51.1, 30.8, 18.8, 15.4. **IR (KBr, cm^{-1}):** 3481, 3412, 2922, 1676, 1138. **GC/MS (*m/z*, %):** 148 (22.8), 137 (15.5), 120 (18.4), 105 (11.1), 84 (35.1), 82 (100.0), 67 (12.3), 54 (39.6), 41 (18.1). Characterization data are in accordance with the literature.[37]

^{1}H and ^{13}C NMR spectra

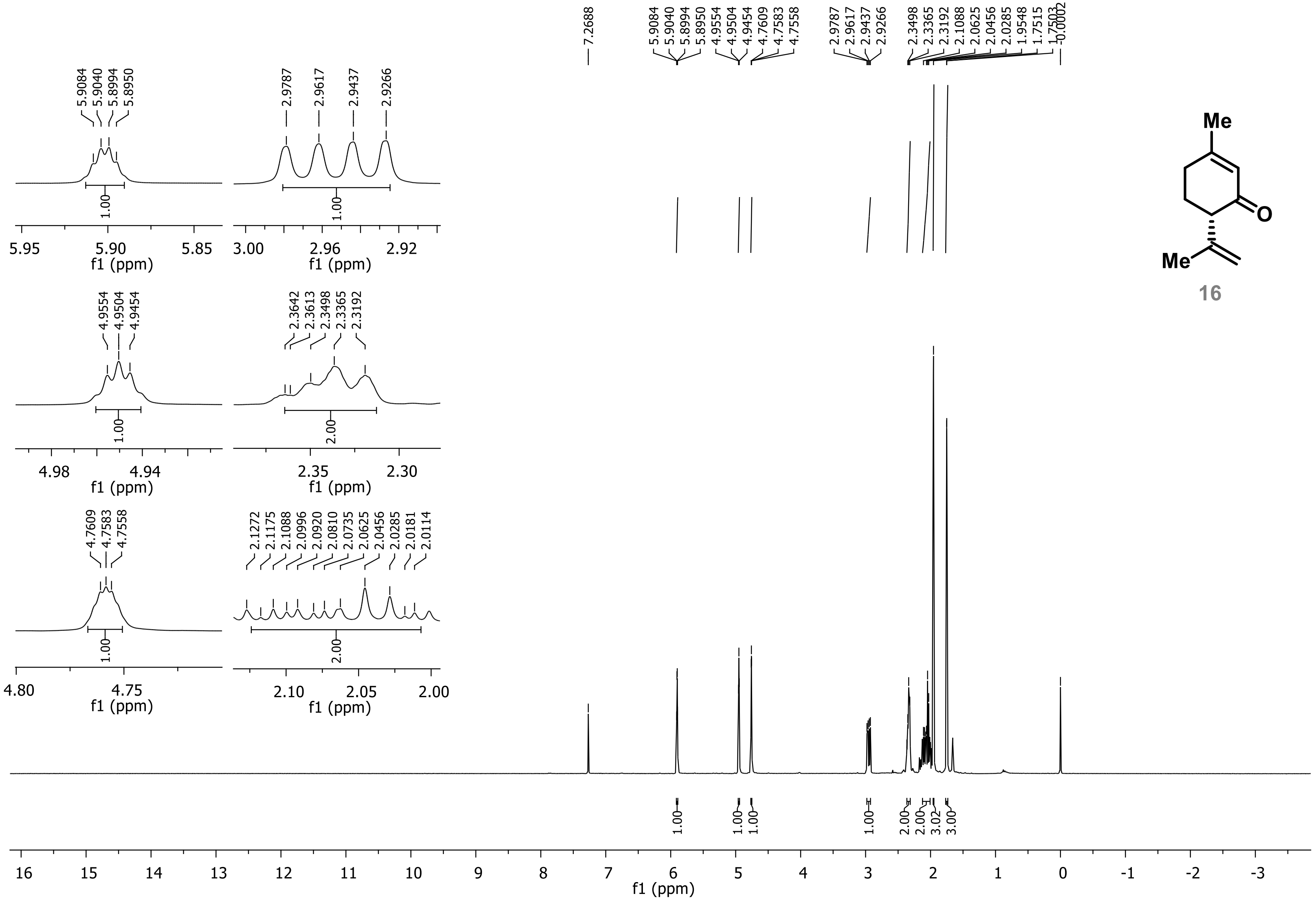

Figure S1. ^{1}H NMR spectrum (300 MHz, $CDCl_3$) of (*S*)-(+)-isopiperitenone (**16**).

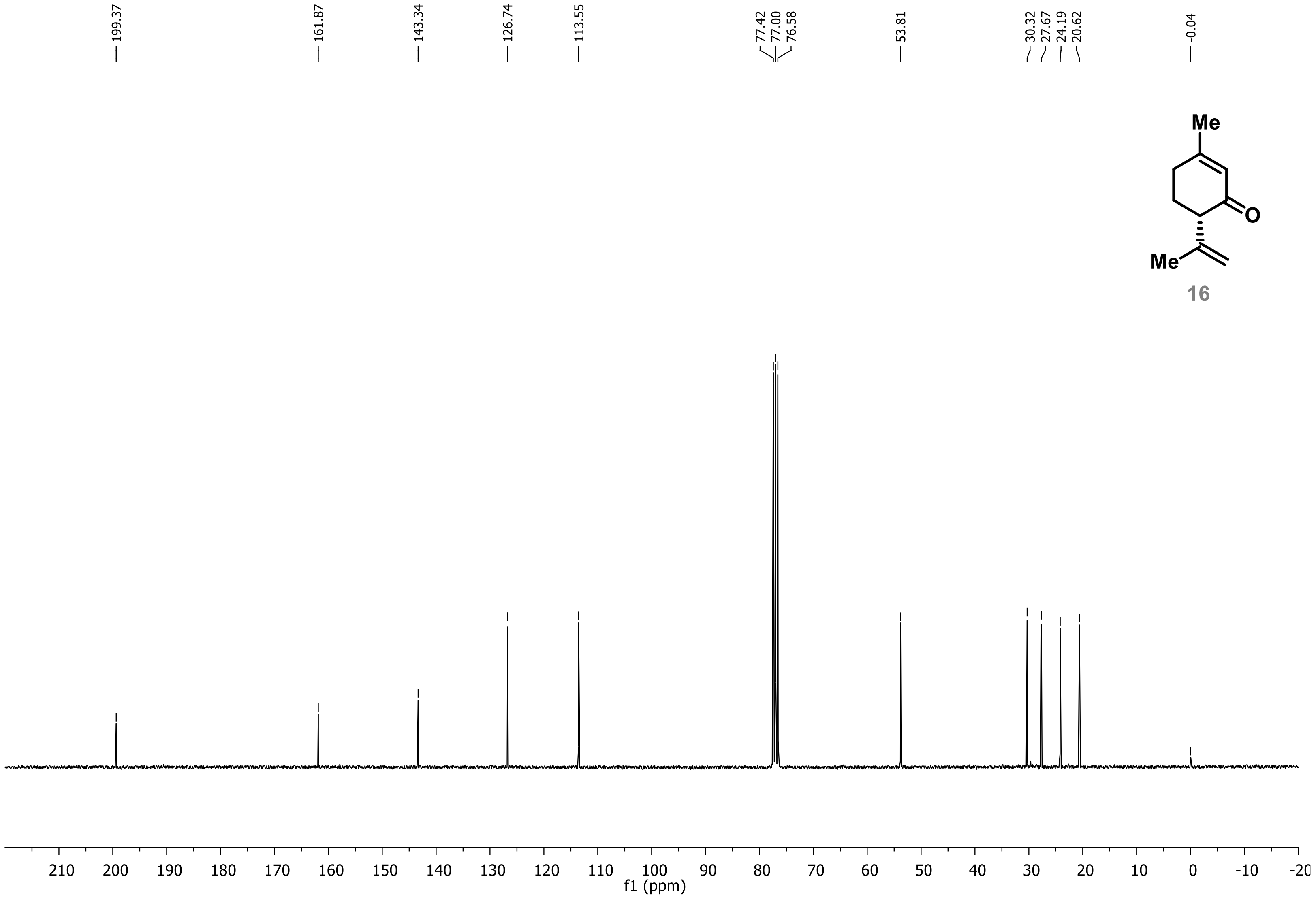

Figure S2. ^{13}C NMR spectrum (75 MHz, $CDCl_3$) of (*S*)-(+)-isopiperitenone (**16**).

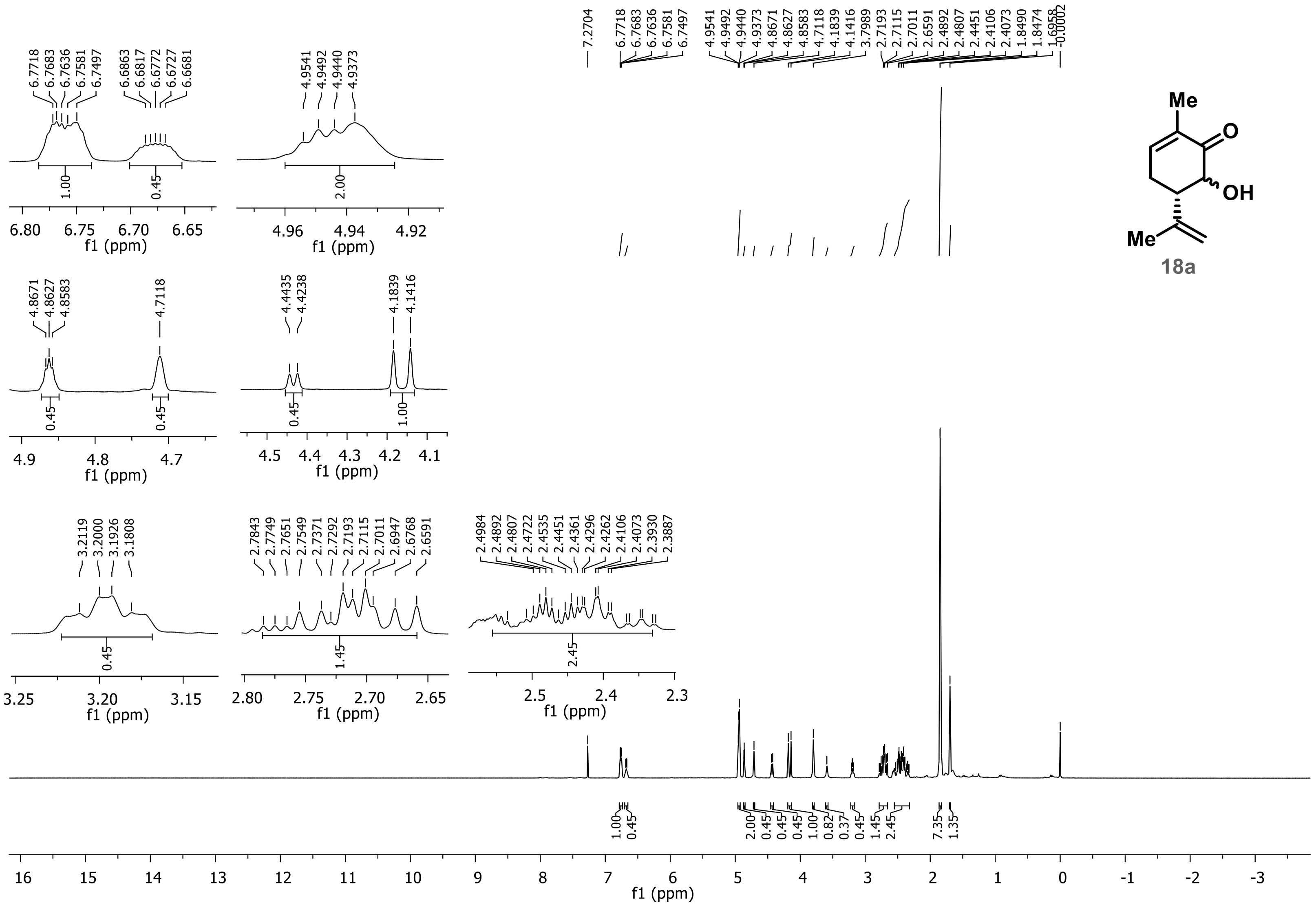

Figure S3. ^{1}H NMR spectrum (300 MHz, $CDCl_3$) of the mixture *trans*- and *cis*-(5*S*)-6-hydroxy-2-methyl-5-(prop-1-en-2-yl)cyclohex-2-enone (**18a**).

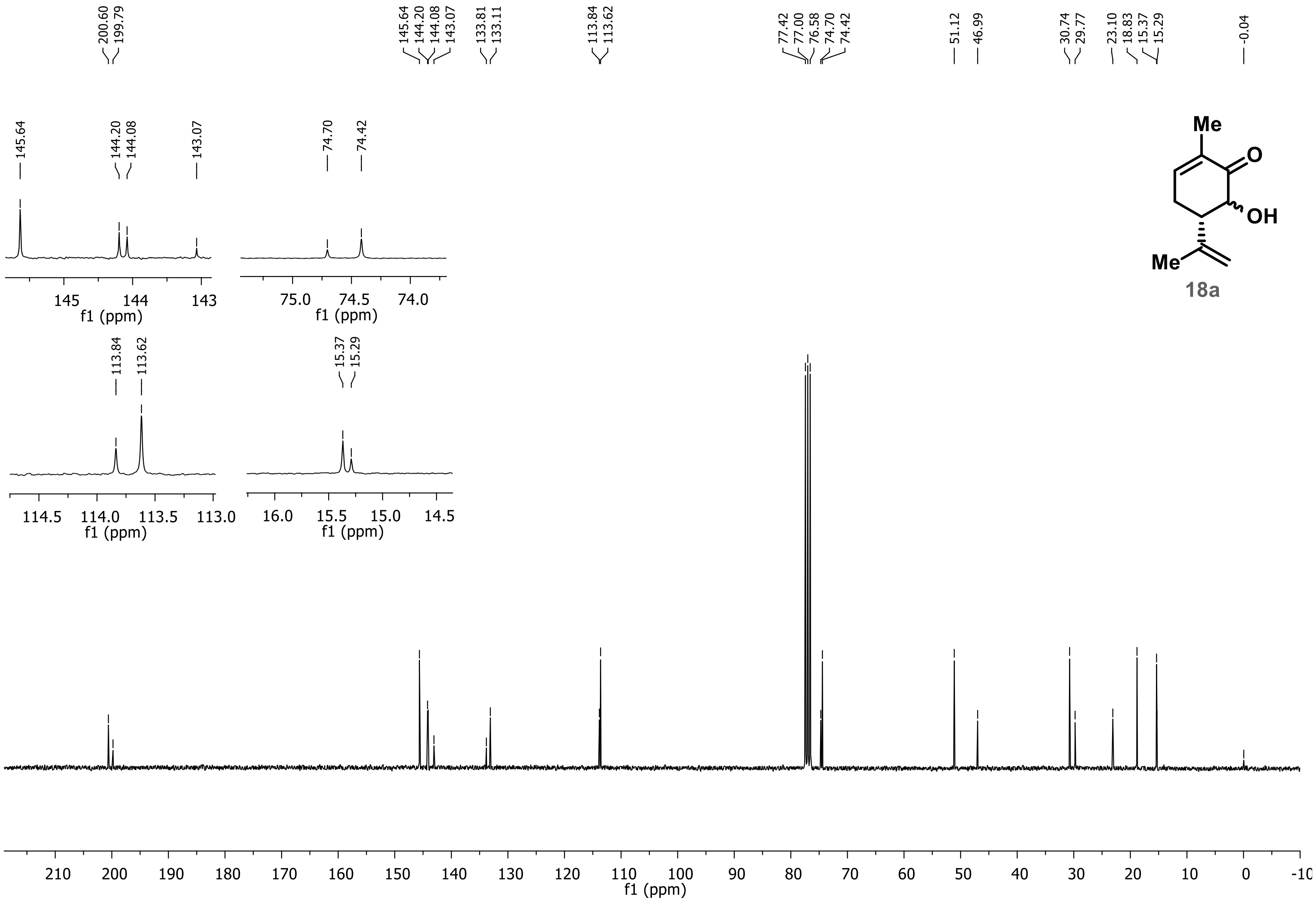

Figure S4. ^{13}C NMR spectrum (75 MHz, $CDCl_3$) of the mixture *trans*- and *cis*-(5*S*)-6-hydroxy-2-methyl-5-(prop-1-en-2-yl)cyclohex-2-enone (**18a**).

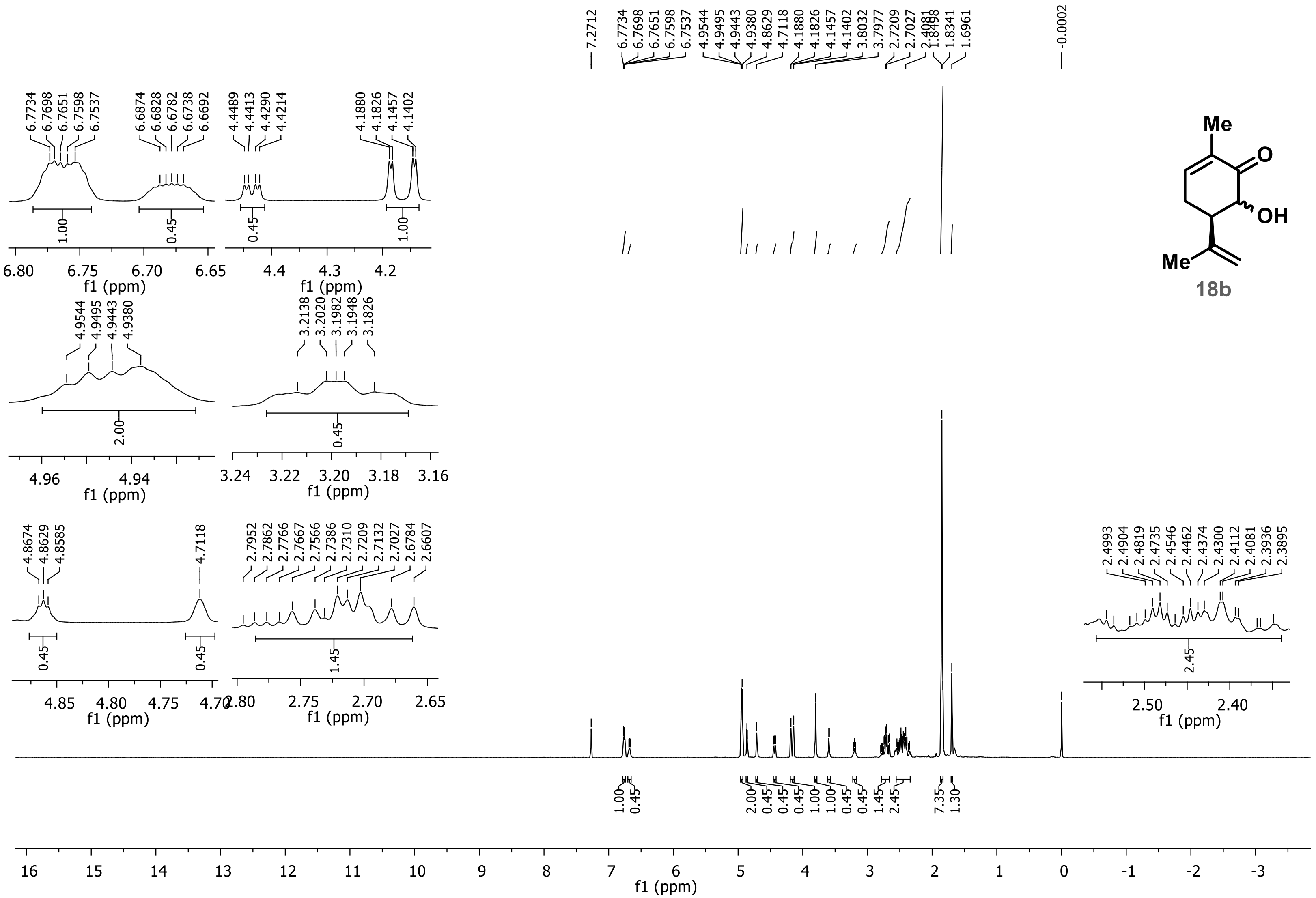

Figure S5. ^{1}H NMR spectrum (300 MHz, $CDCl_3$) of the mixture *trans*- and *cis*-(5*R*)-6-hydroxy-2-methyl-5-(prop-1-en-2-yl)cyclohex-2-enone (**18b**).

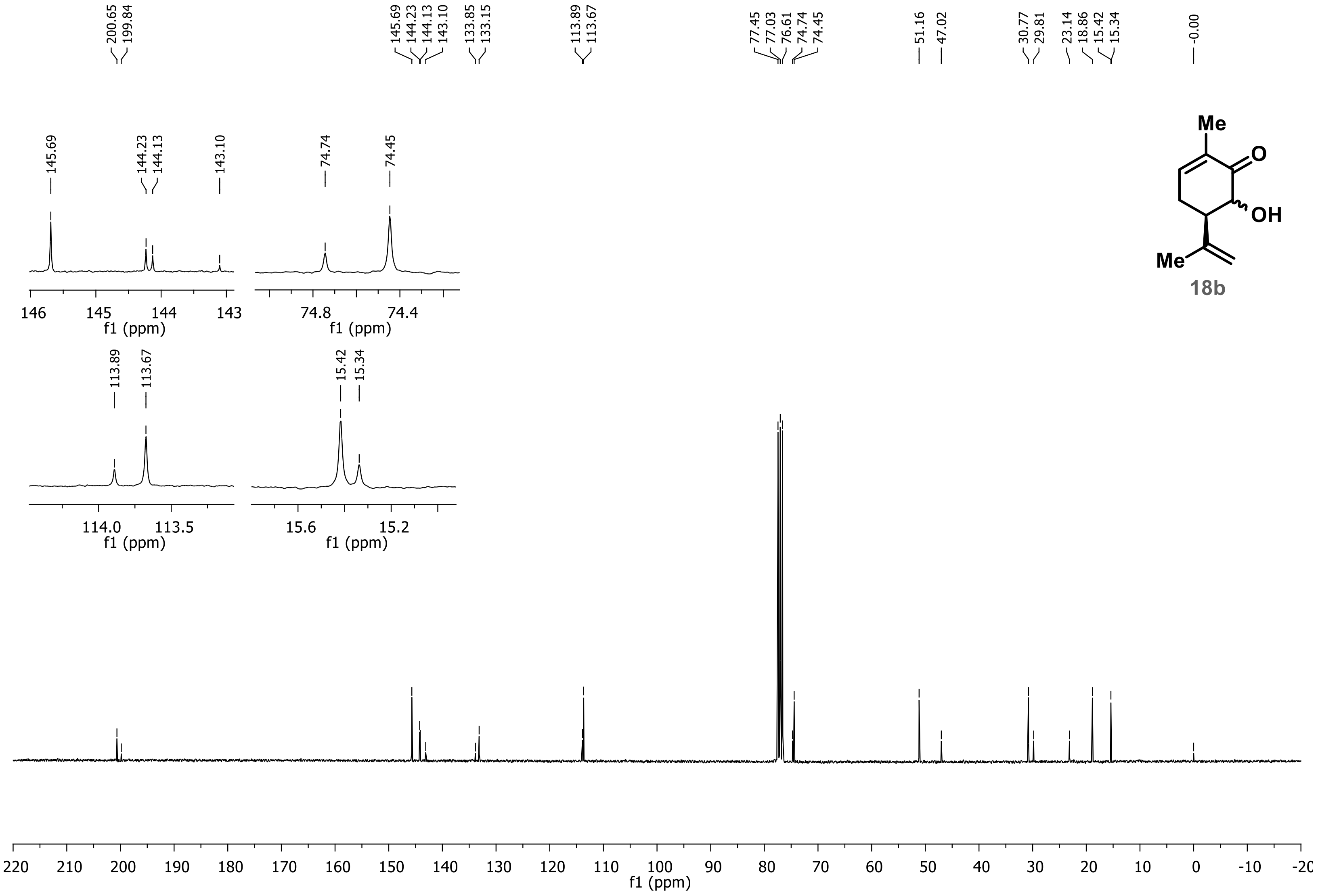

Figure S6. ^{13}C NMR spectrum (75 MHz, $CDCl_3$) of the mixture *trans*- and *cis*-(5*R*)-6-hydroxy-2-methyl-5-(prop-1-en-2-yl)cyclohex-2-enone (**18b**).

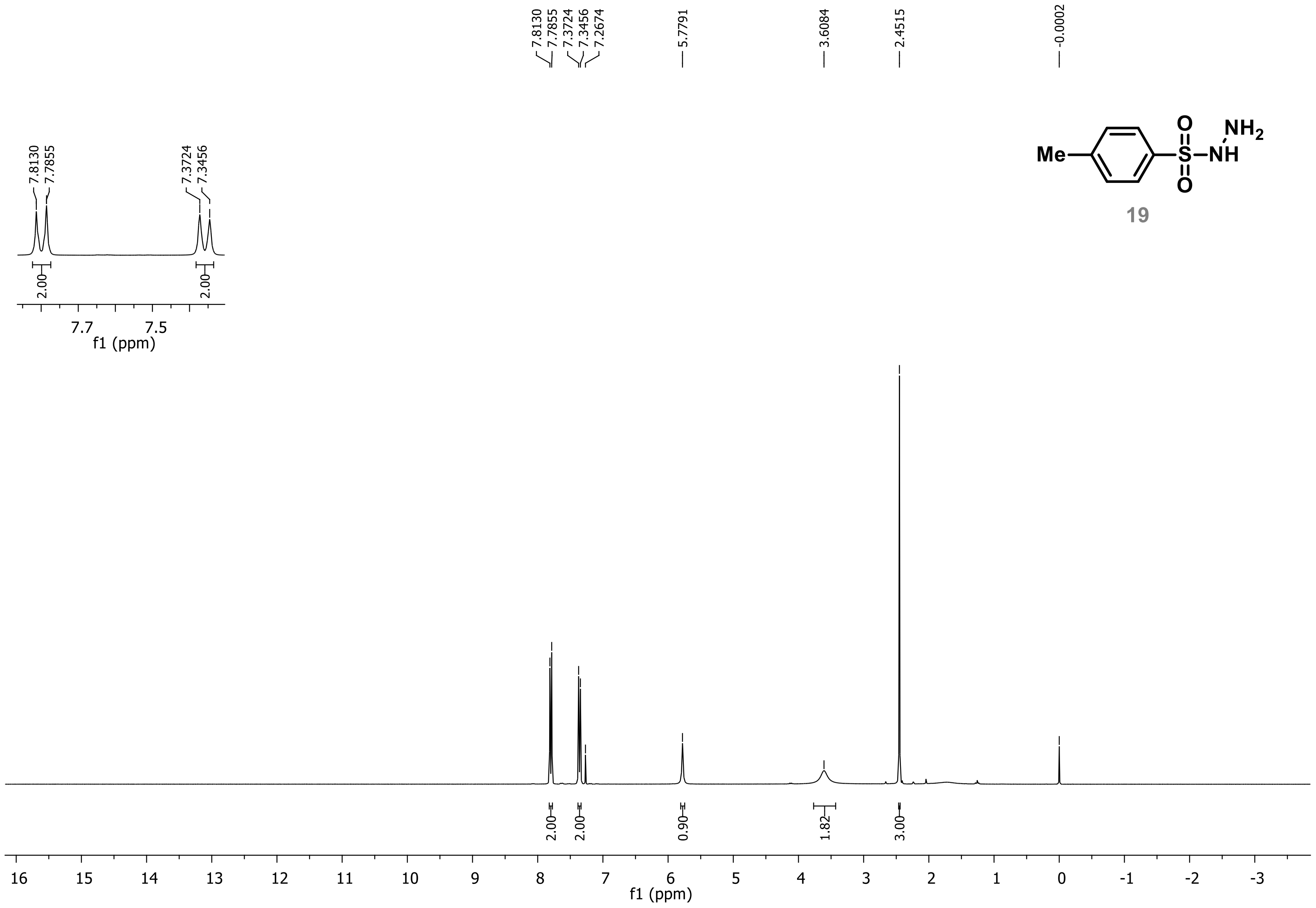

Figure S7. ^{1}H NMR spectrum (300 MHz, $CDCl_3$) of *p*-tosylhydrazide (**19**).

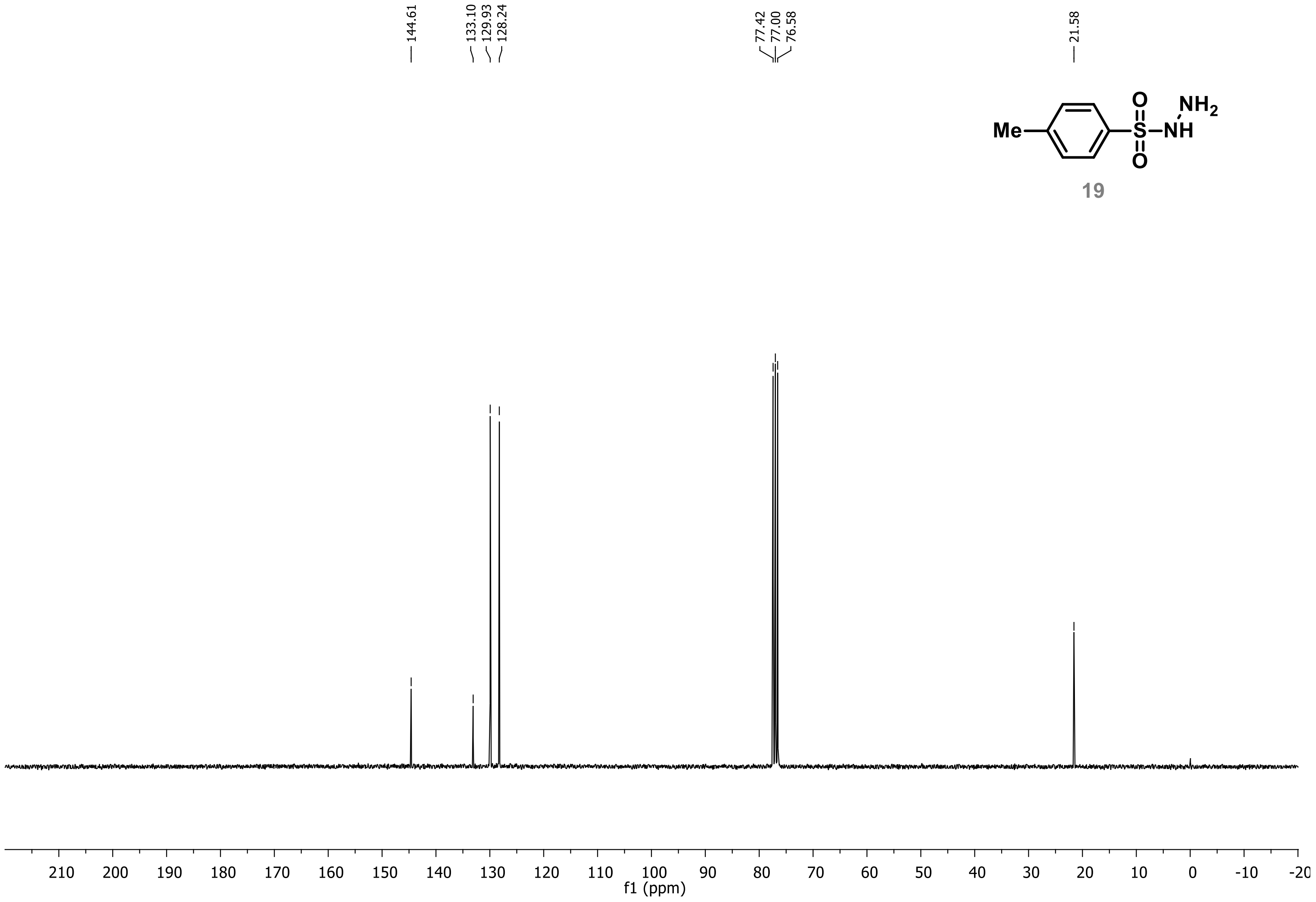

Figure S8. ^{13}C NMR spectrum (75 MHz, $CDCl_3$) of *p*-tosylhydrazide (**19**).

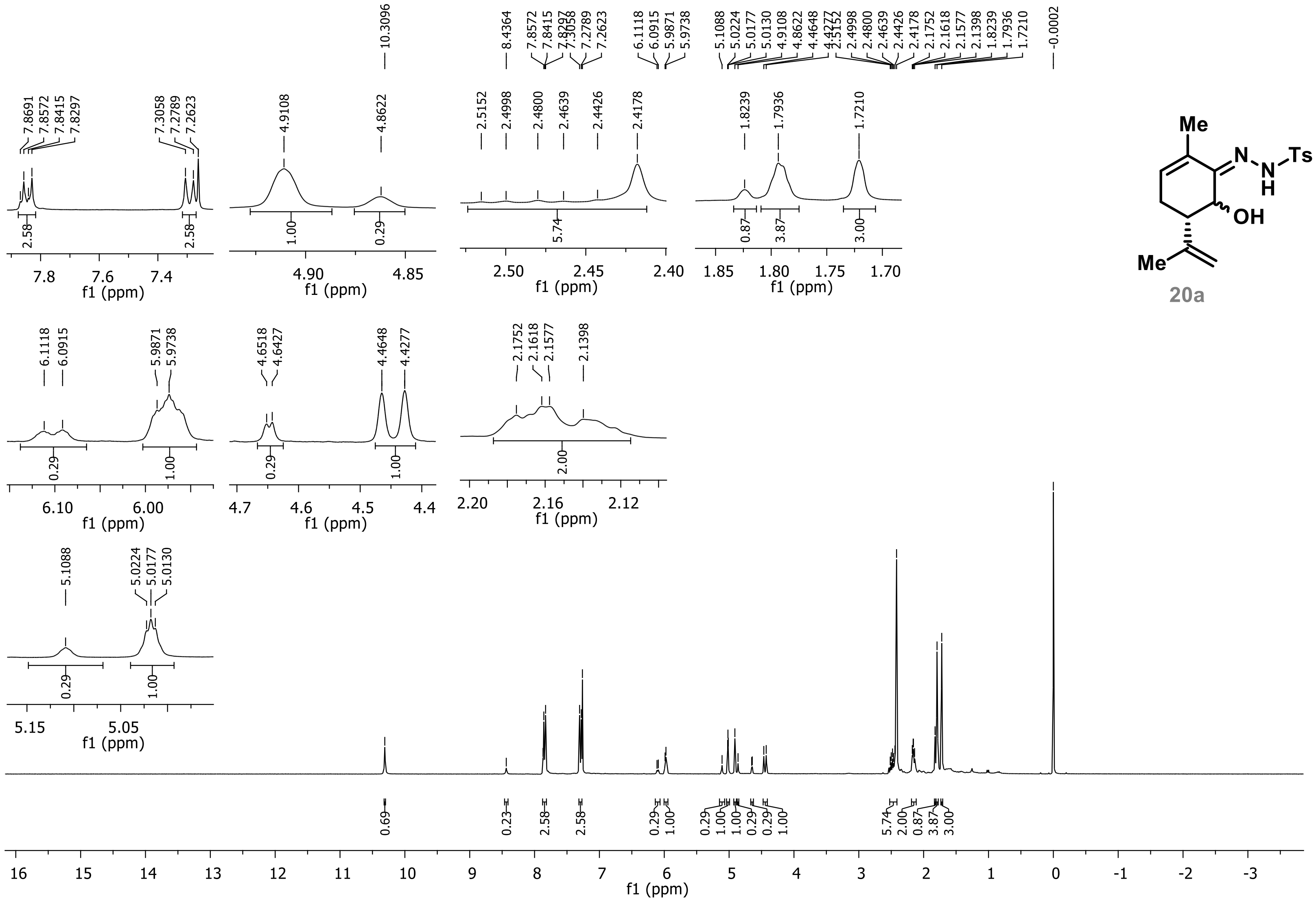

Figure S9. ^{1}H NMR spectrum (300 MHz, $CDCl_3$) of the mixture *trans*- and *cis*-*N*'-((5*S*)-6-hydroxy-2-methyl-5-(prop-1-en-2-yl)cyclohex-2-en-1-ylidene)-4-methylbenzenesulfonohydrazide (**20a**).

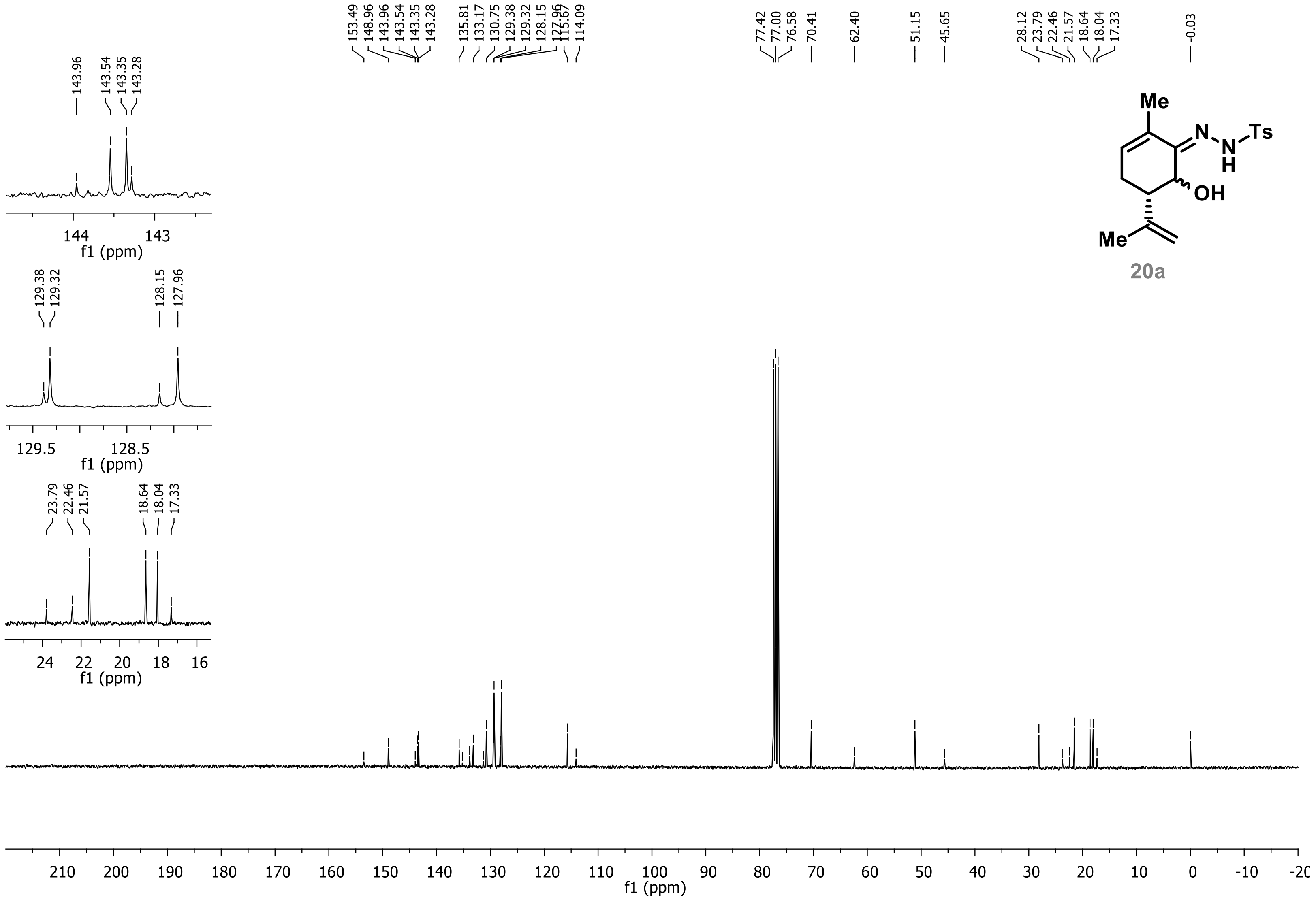

Figure S10. ^{13}C NMR spectrum (75 MHz, $CDCl_3$) of the mixture *trans*- and *cis*-*N*'-((5*S*)-6-hydroxy-2-methyl-5-(prop-1-en-2-yl)cyclohex-2-en-1-ylidene)-4-methylbenzenesulfonohydrazide (**20a**).

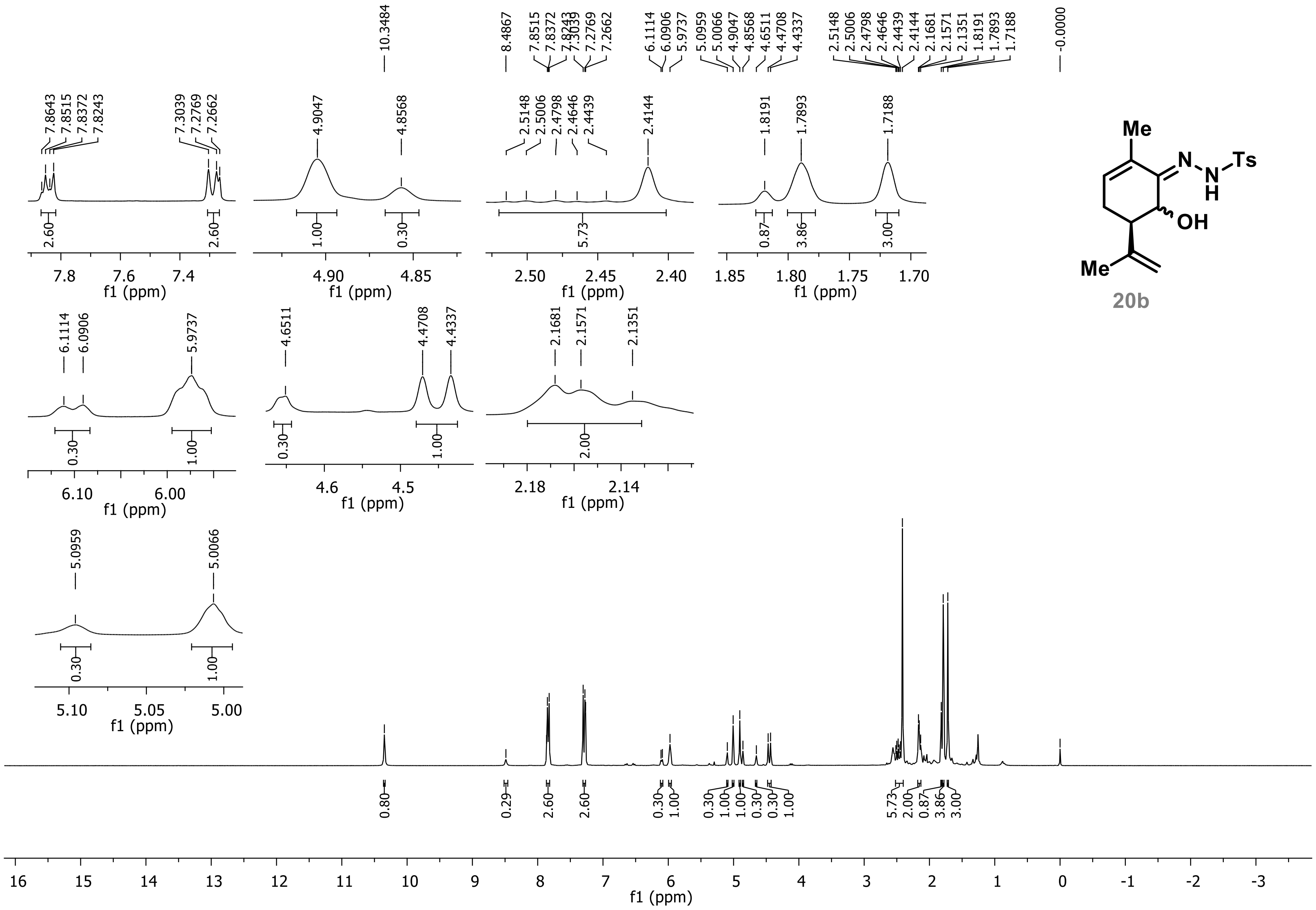

20b

Figure S11. ^{1}H NMR spectrum (300 MHz, $CDCl_3$) of the mixture *trans*- and *cis*-*N*'-((5*R*)-6-hydroxy-2-methyl-5-(prop-1-en-2-yl)cyclohex-2-en-1-ylidene)-4-methylbenzenesulfonohydrazide (**20b**).

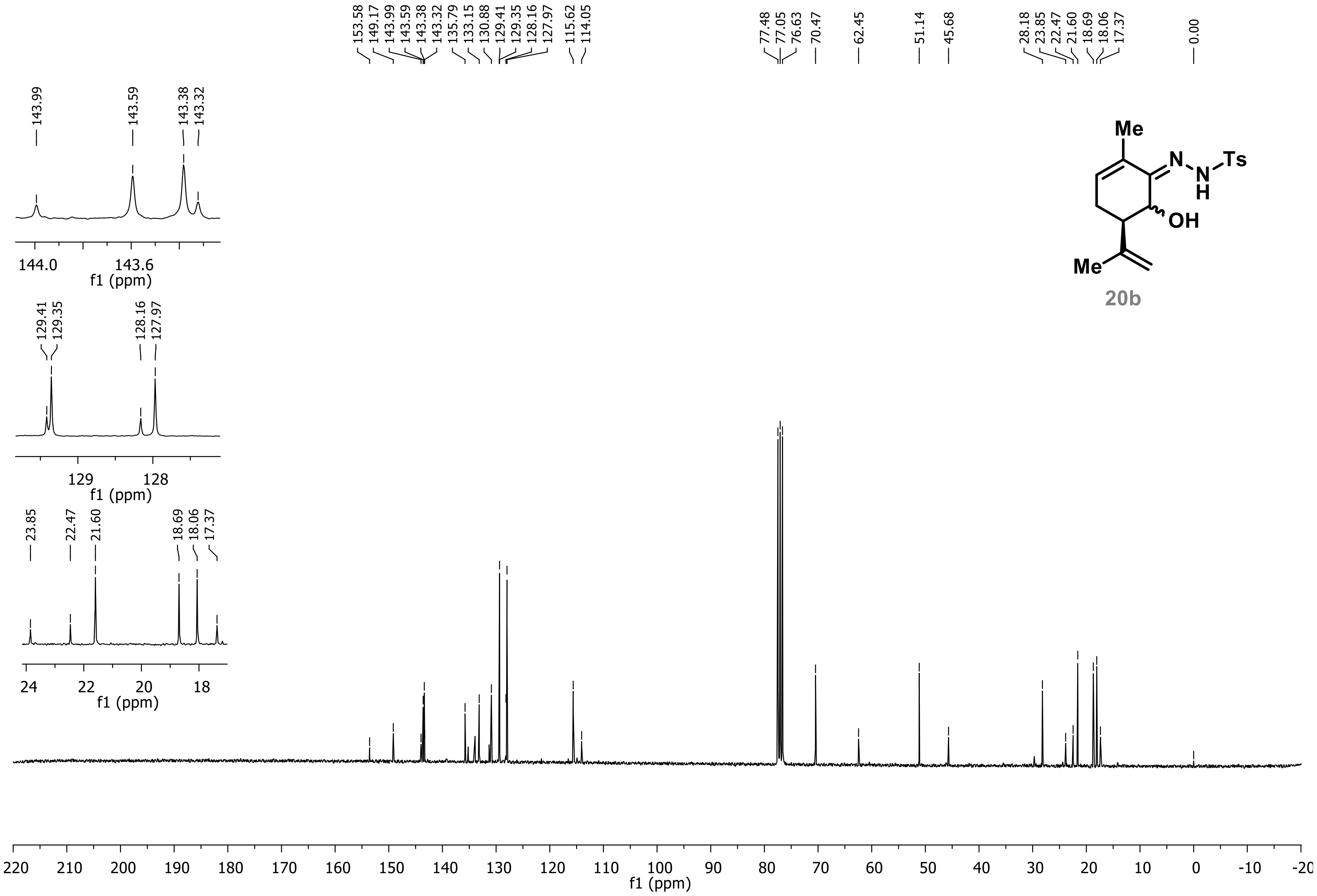

Figure S12. ^{13}C NMR spectrum (75 MHz, $CDCl_3$) of the mixture *trans*- and *cis*-*N'*-((5*R*)-6-hydroxy-2-methyl-5-(prop-1-en-2-yl)cyclohex-2-en-1-ylidene)-4-methylbenzenesulfonohydrazide (**20b**).

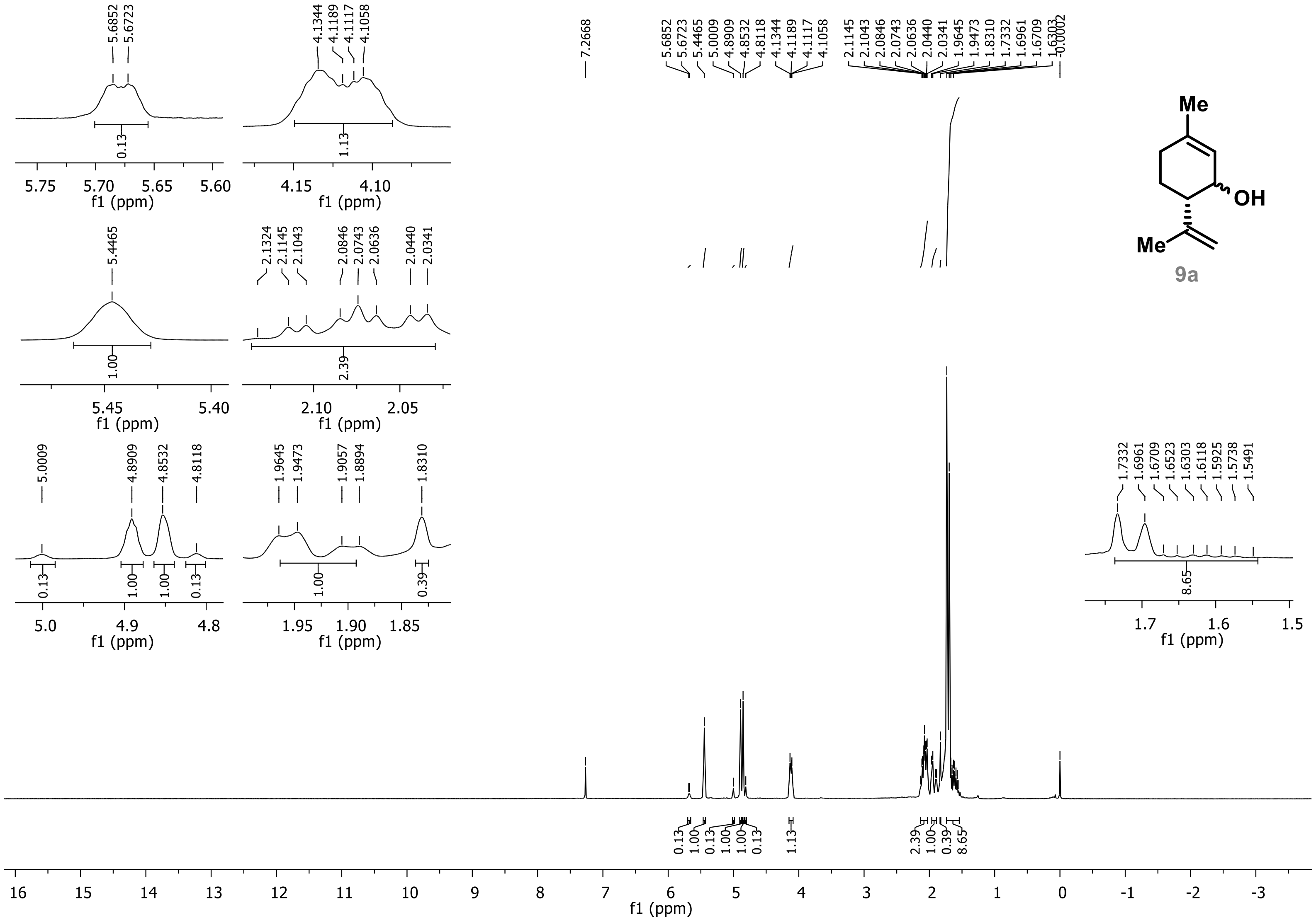

Figure S13. ^{1}H NMR spectrum (300 MHz, $CDCl_3$) of the mixture *trans*- and *cis*-(6*S*)-isopiperitenol (**9a**).

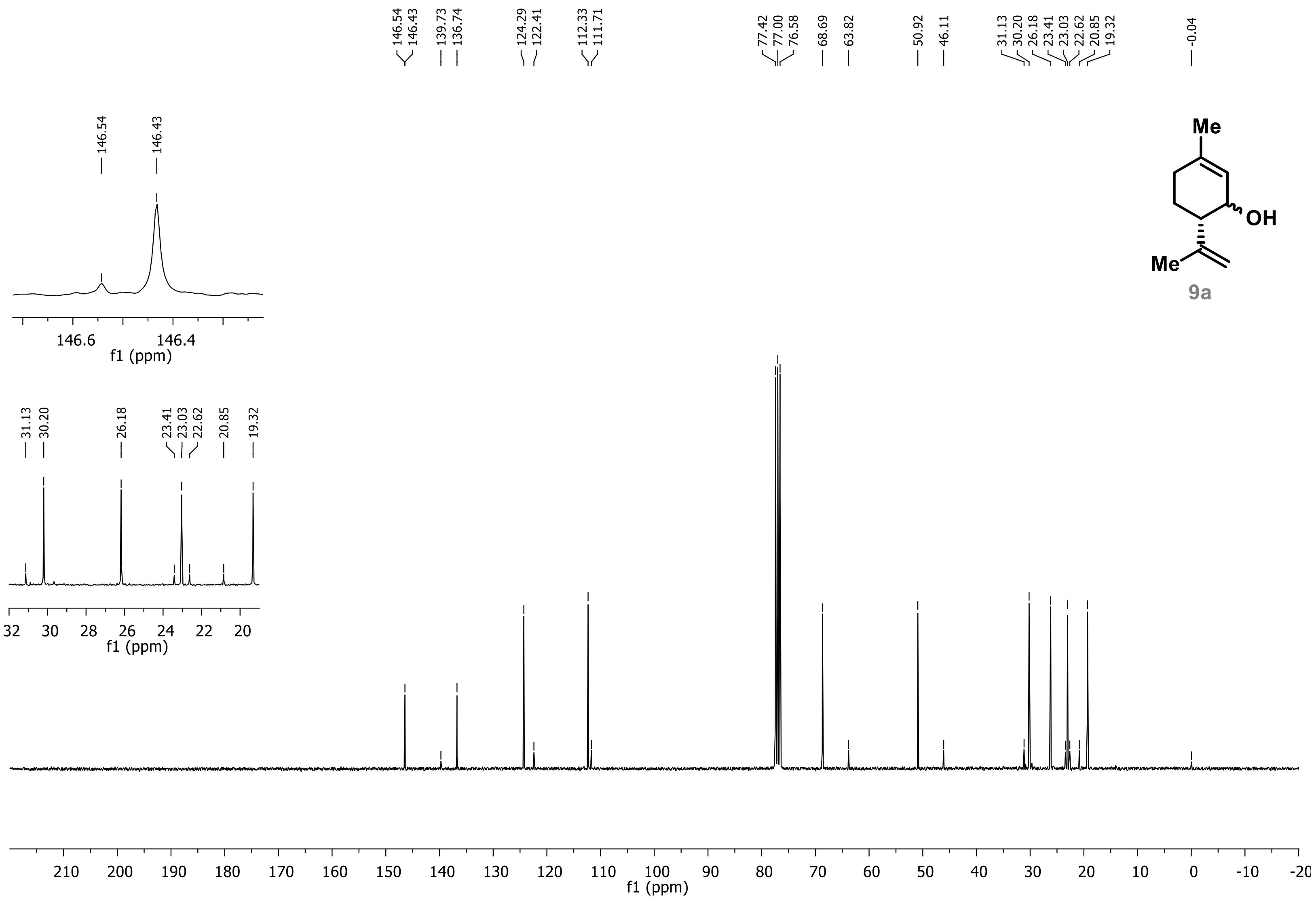

Figure S14. ^{13}C NMR spectrum (75 MHz, $CDCl_3$) of the mixture *trans*- and *cis*-(6*S*)-isopiperitenol (**9a**).

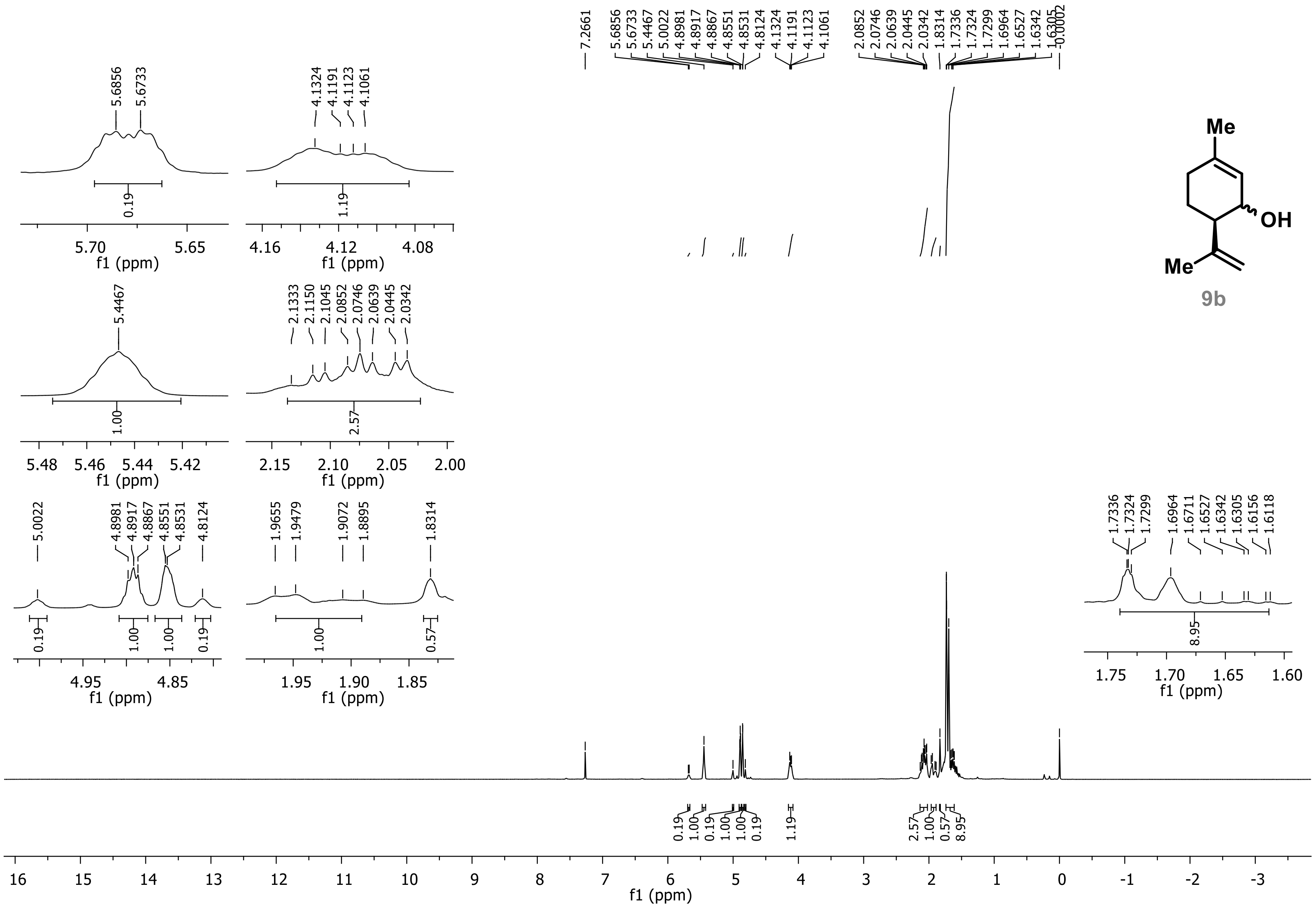

Figure S15. ^{1}H NMR spectrum (300 MHz, $CDCl_3$) of the mixture *trans*- and *cis*-(6*R*)-isopiperitenol (**9b**).

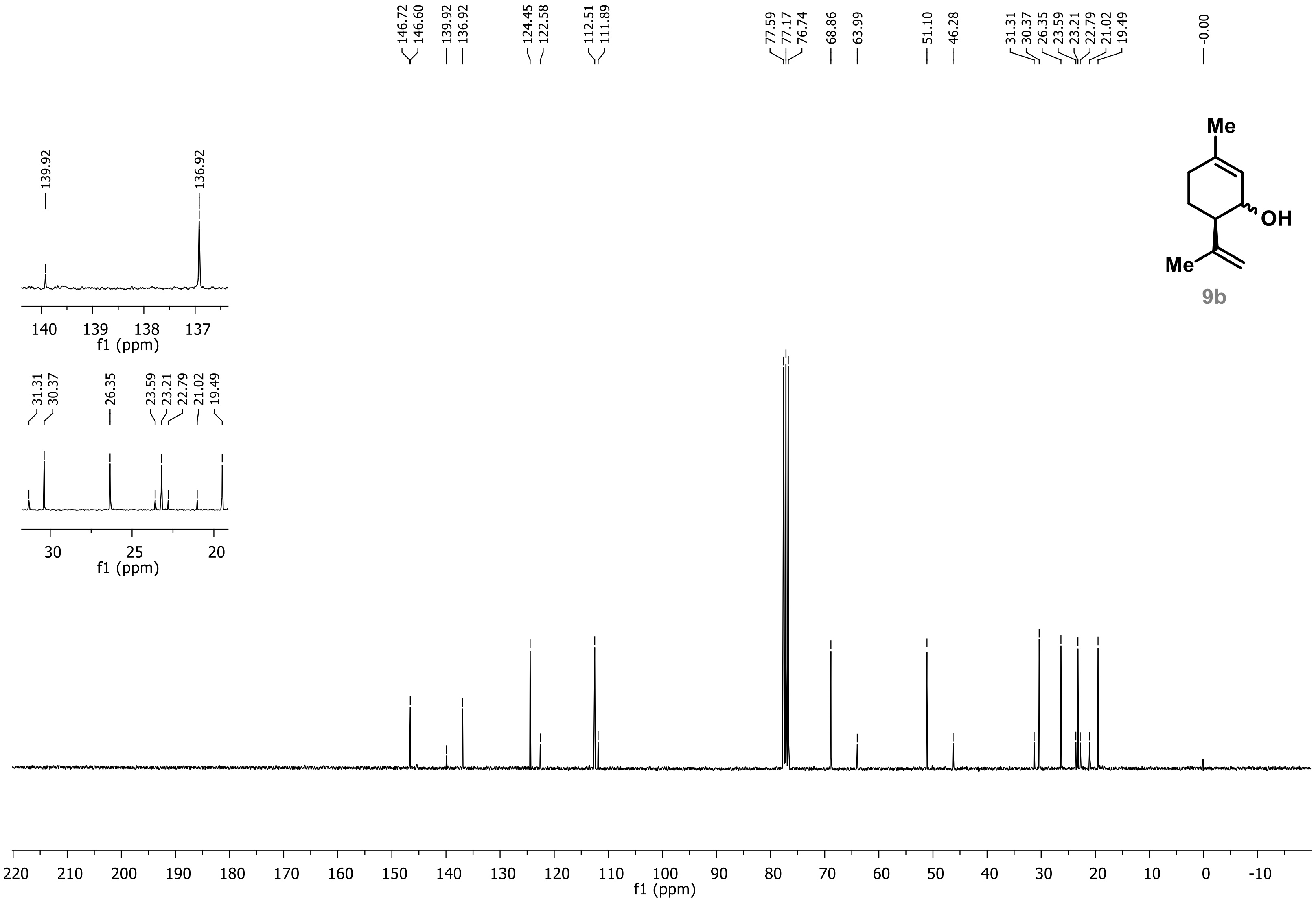

Figure S16. ^{13}C NMR spectrum (75 MHz, $CDCl_3$) of the mixture *trans*- and *cis*-(6*R*)-isopiperitenol (**9b**).

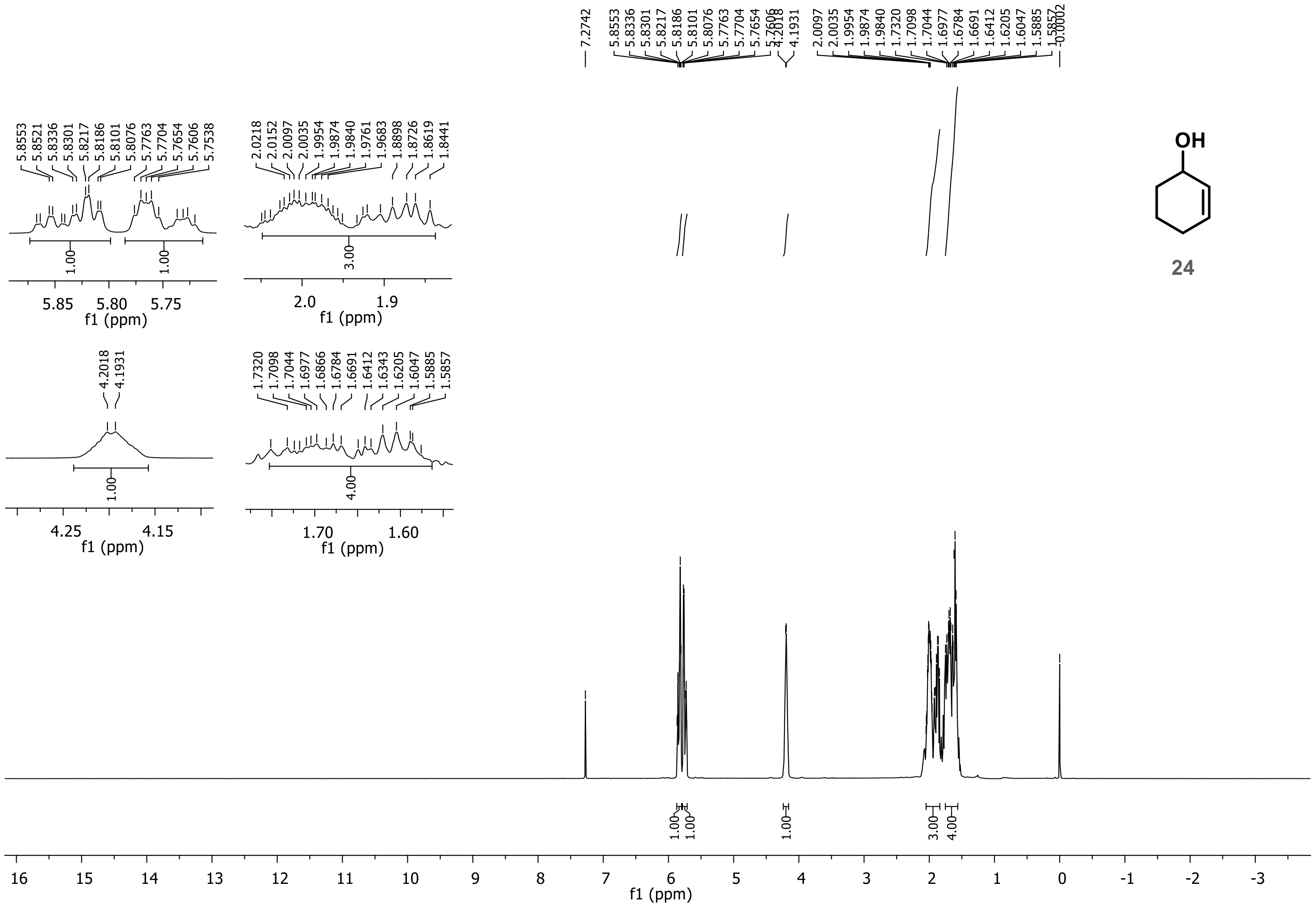

Figure S17. ^1H NMR spectrum (300 MHz, $CDCl_3$) of cyclohex-2-enol (**24**).

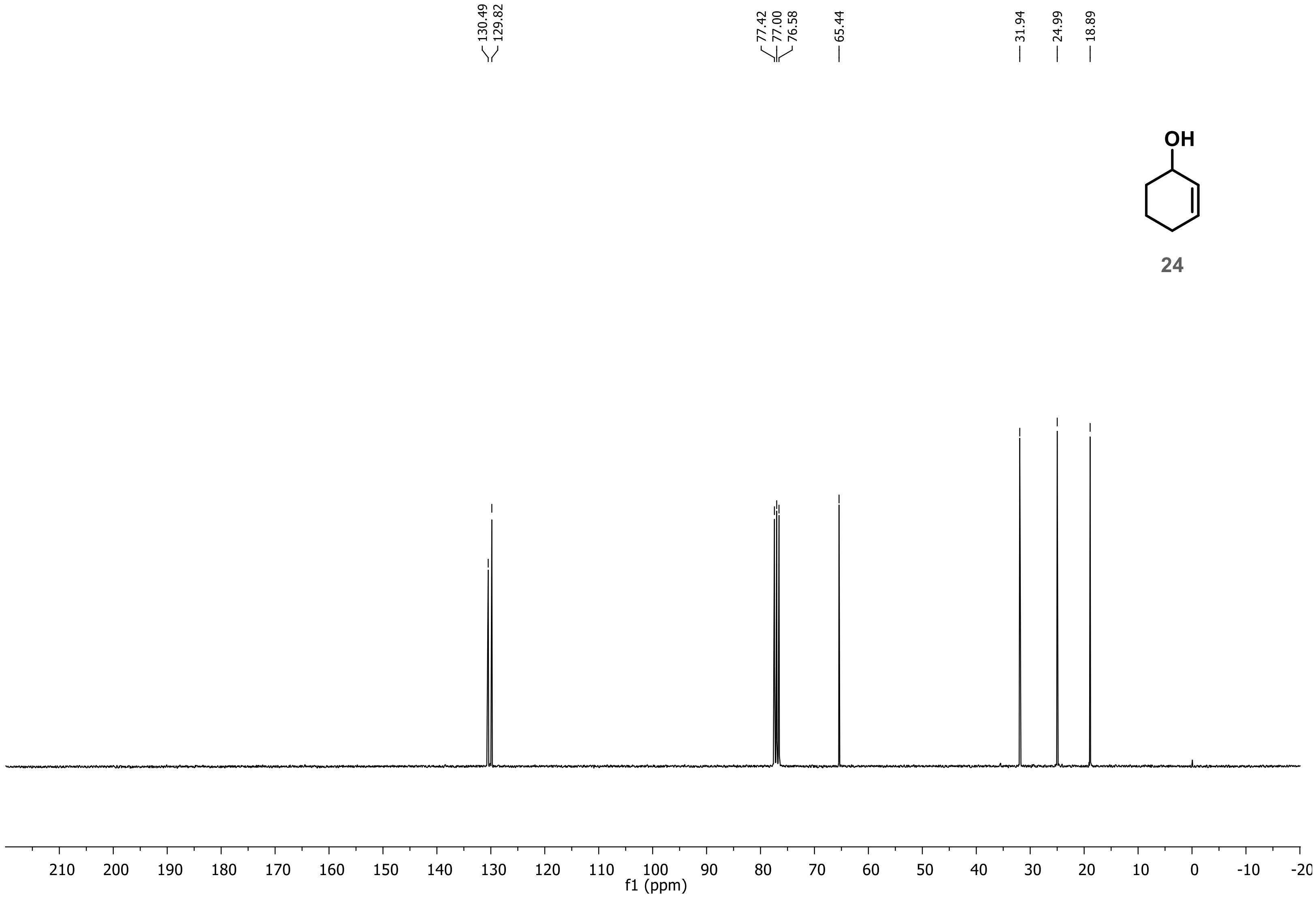

Figure S18. ^{13}C NMR spectrum (75 MHz, $CDCl_3$) of cyclohex-2-enol (**24**).

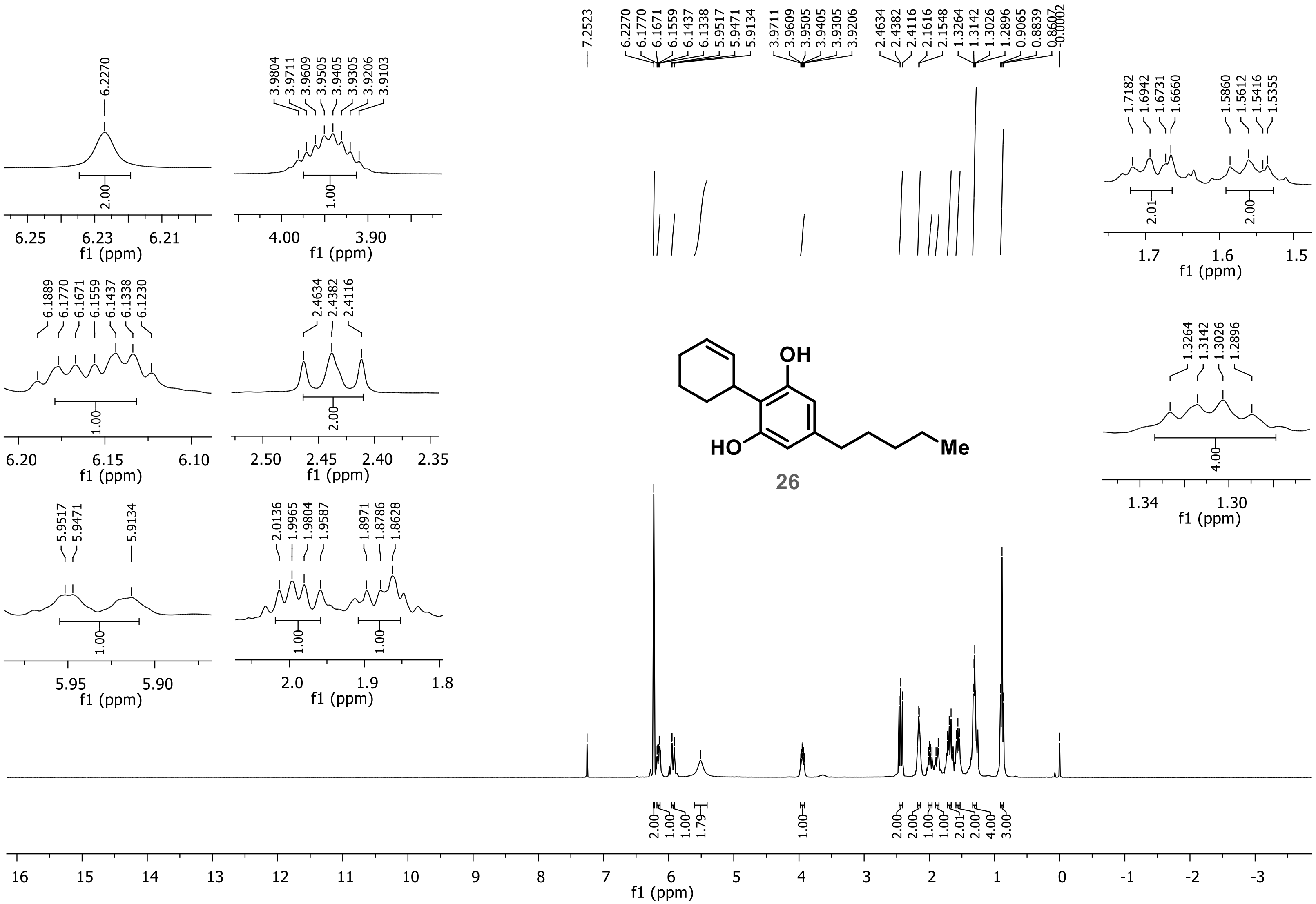

Figure S19. ^{1}H NMR spectrum (300 MHz, $CDCl_3$) of 2-(cyclohex-2-enyl)-5-pentylbenzene-1,3-diol (**26**).

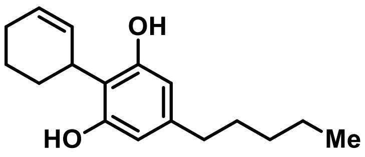

26

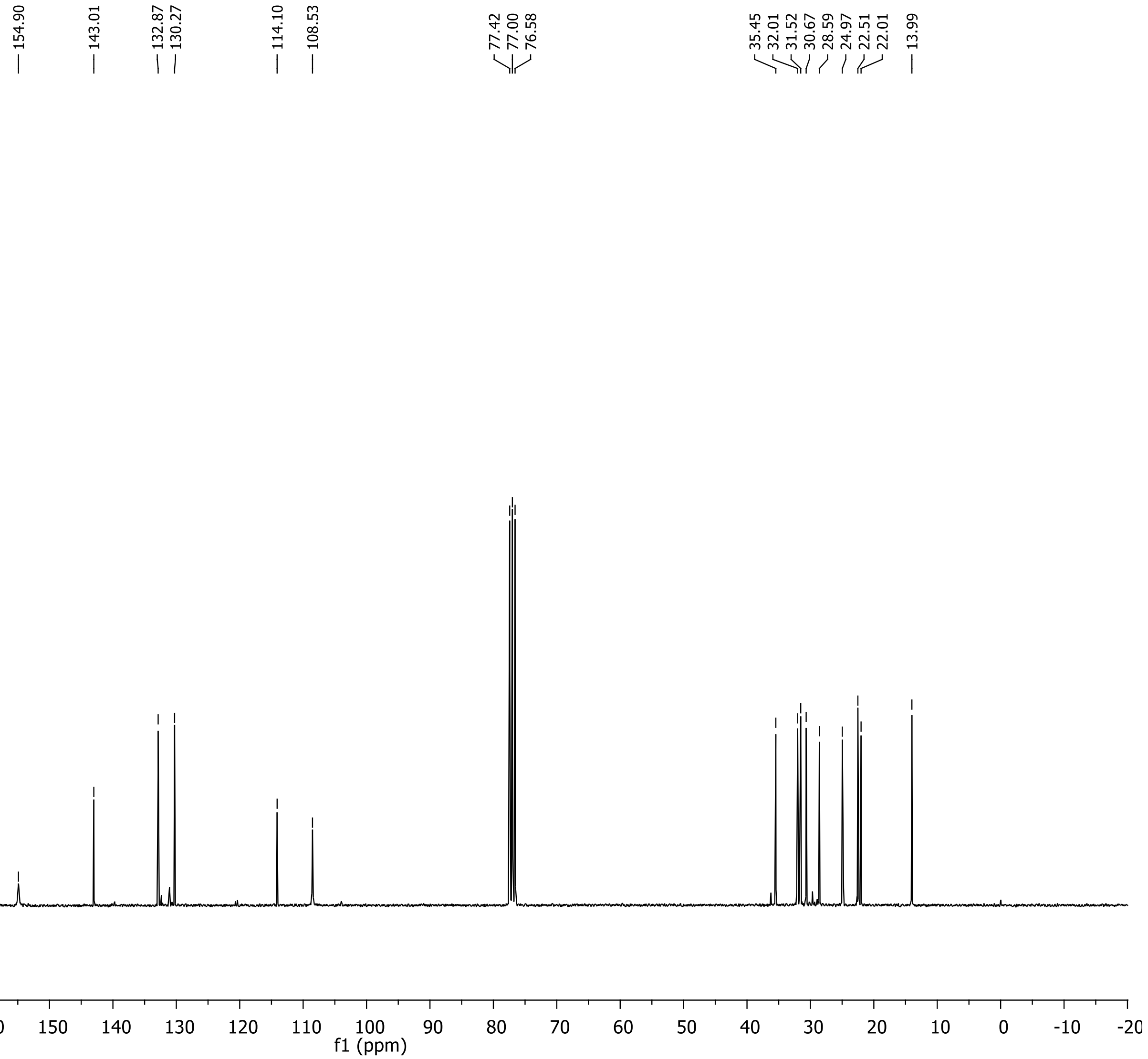

Figure S20. ^{13}C NMR spectrum (75 MHz, $CDCl_3$) of 2-(cyclohex-2-enyl)-5-pentylbenzene-1,3-diol (**26**).

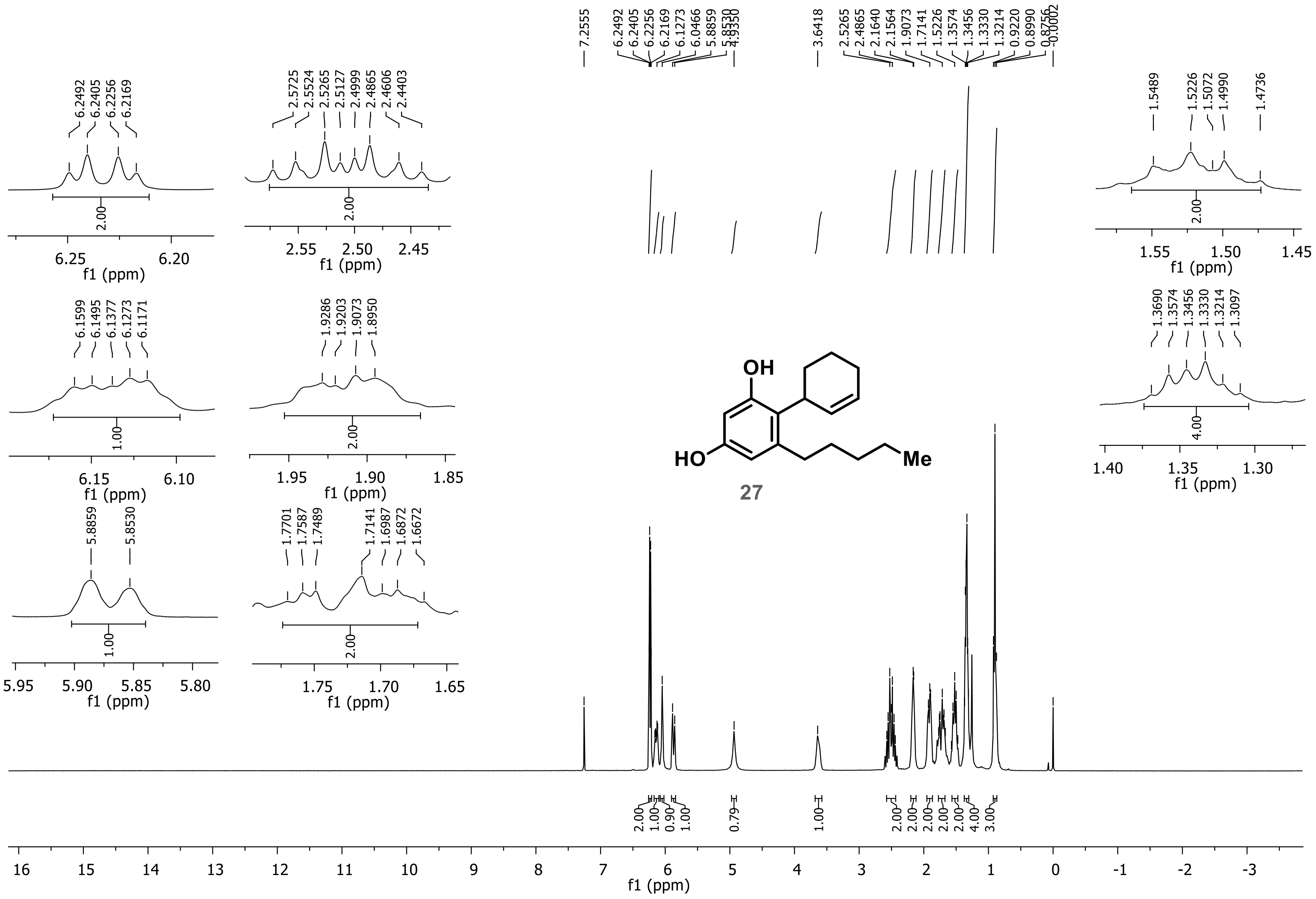

Figure S21. ^{1}H NMR spectrum (300 MHz, $CDCl_3$) of 4-(cyclohex-2-enyl)-5-pentylbenzene-1,3-diol (**27**).

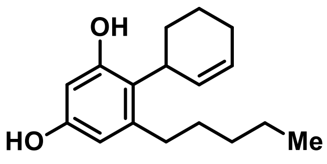

27

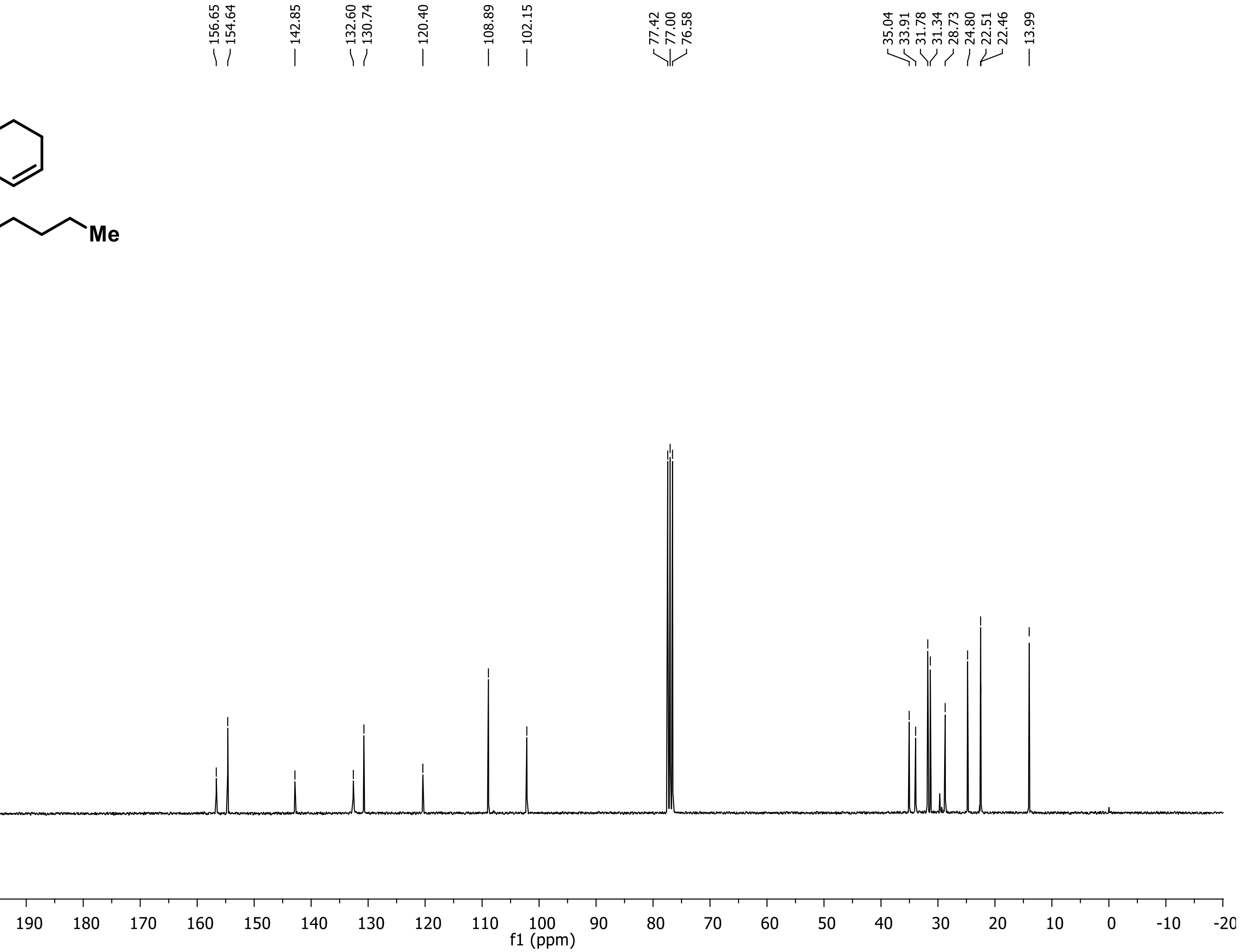

Figure S22. ^{13}C NMR spectrum (75 MHz, $CDCl_3$) of 4-(cyclohex-2-enyl)-5-pentylbenzene-1,3-diol (**27**).

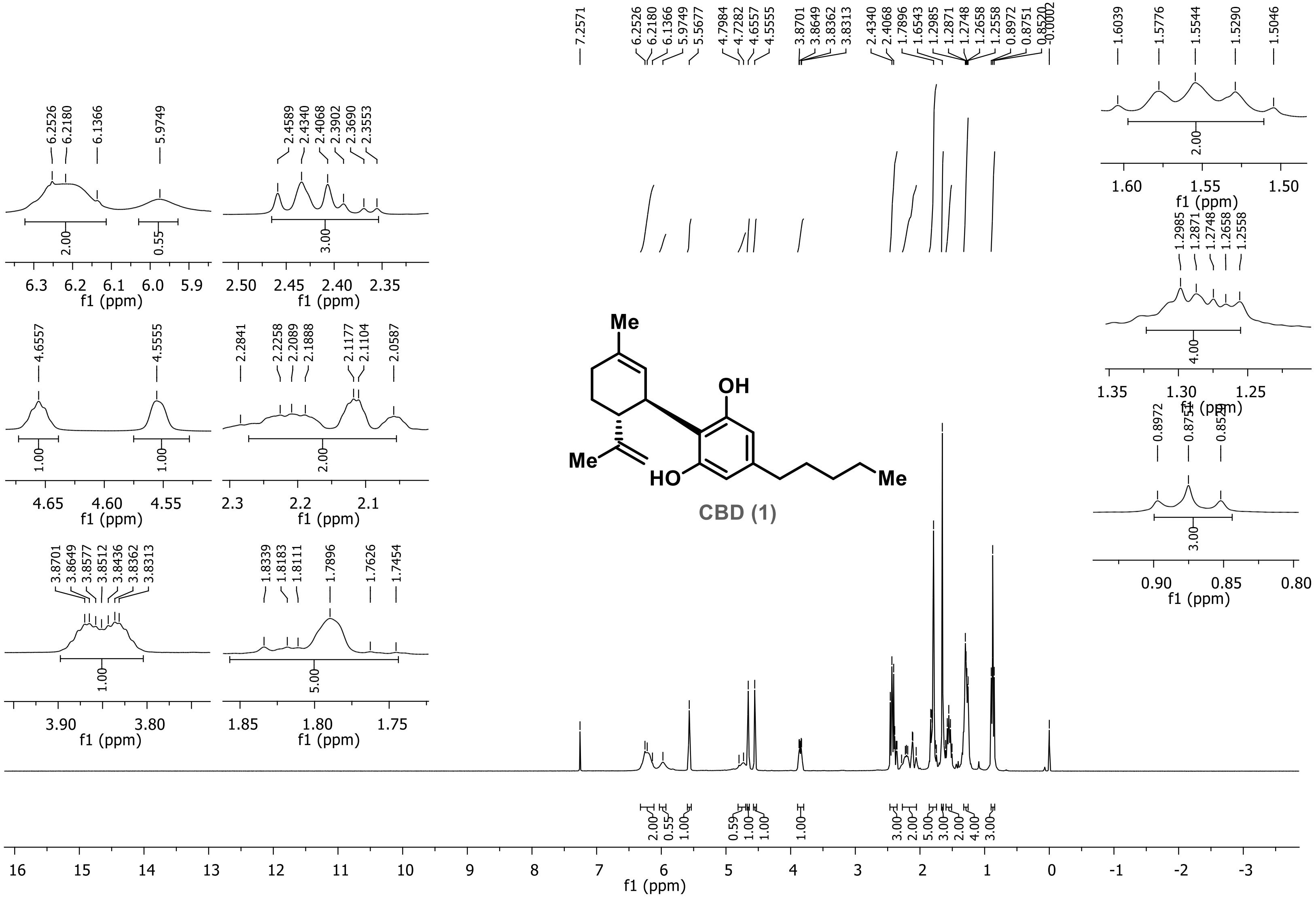

Figure S23. ^{1}H NMR spectrum (300 MHz, $CDCl_3$) of (-)-cannabidiol (CBD, **1**).

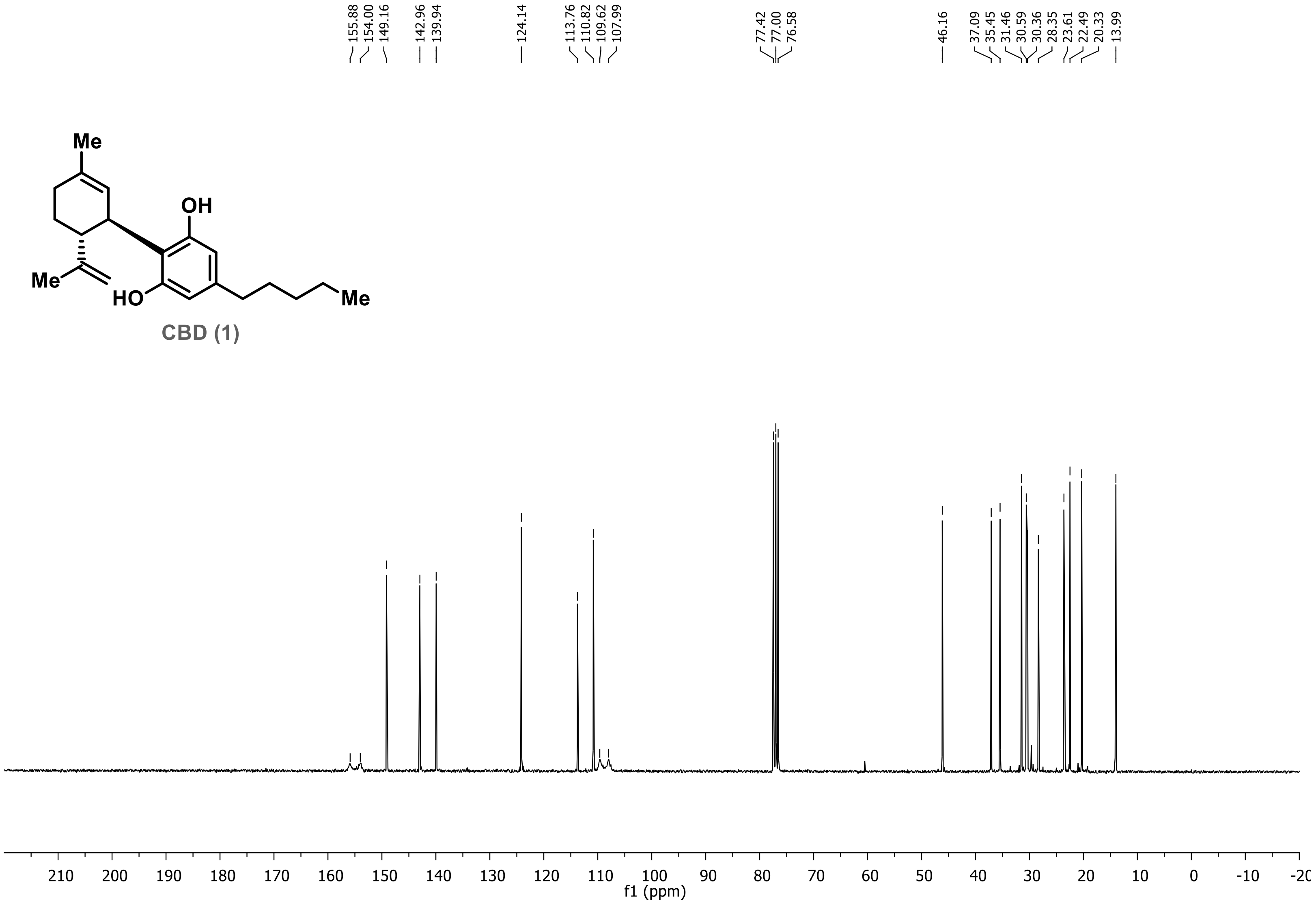

Figure S24. ^{13}C NMR spectrum (75 MHz, $CDCl_3$) of (-)-cannabidiol (CBD, **1**).

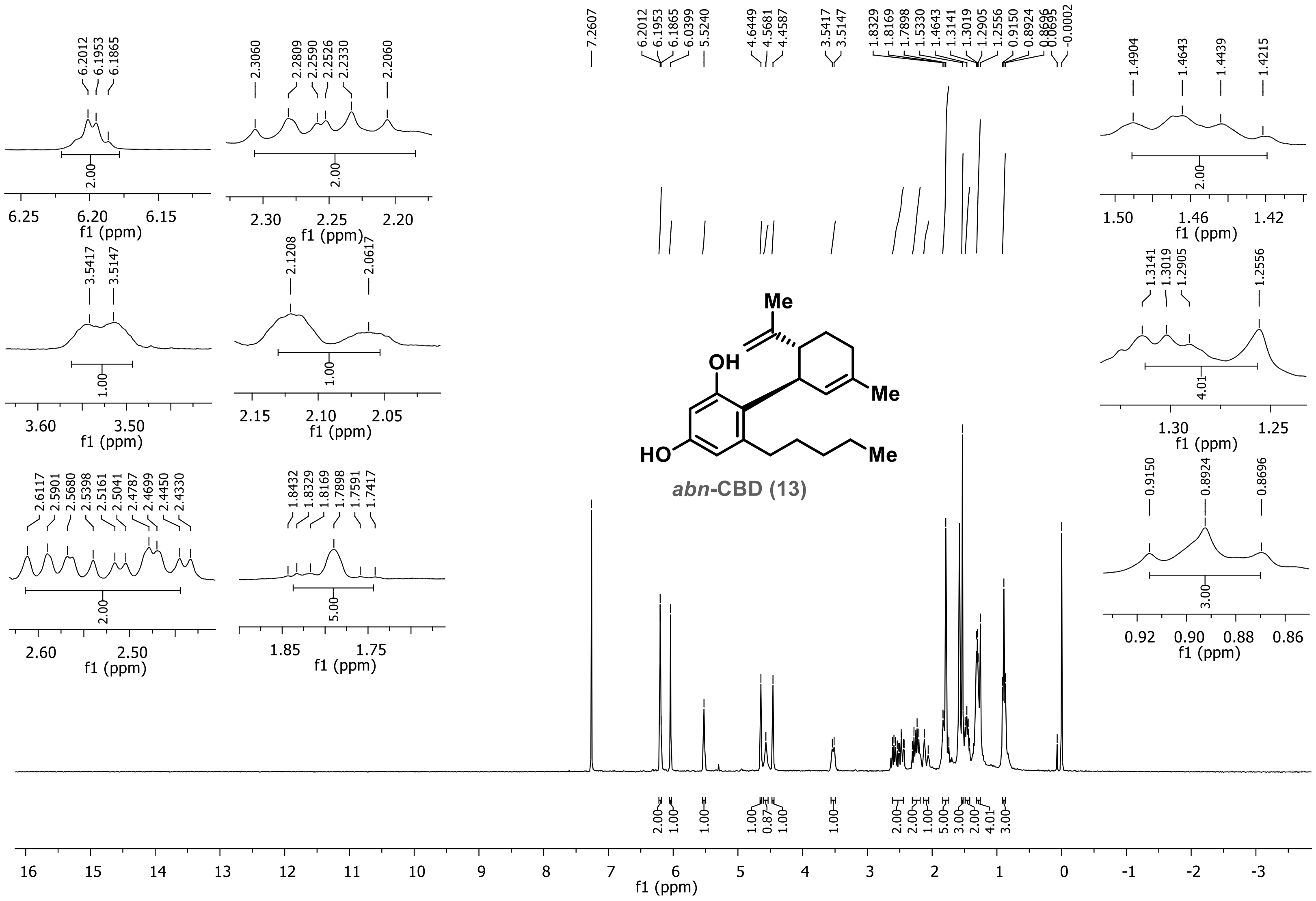

Figure S25. ¹H NMR spectrum (300 MHz, $CDCl_3$) of (-)-abnormal cannabidiol (*abn*-CBD, **13**).

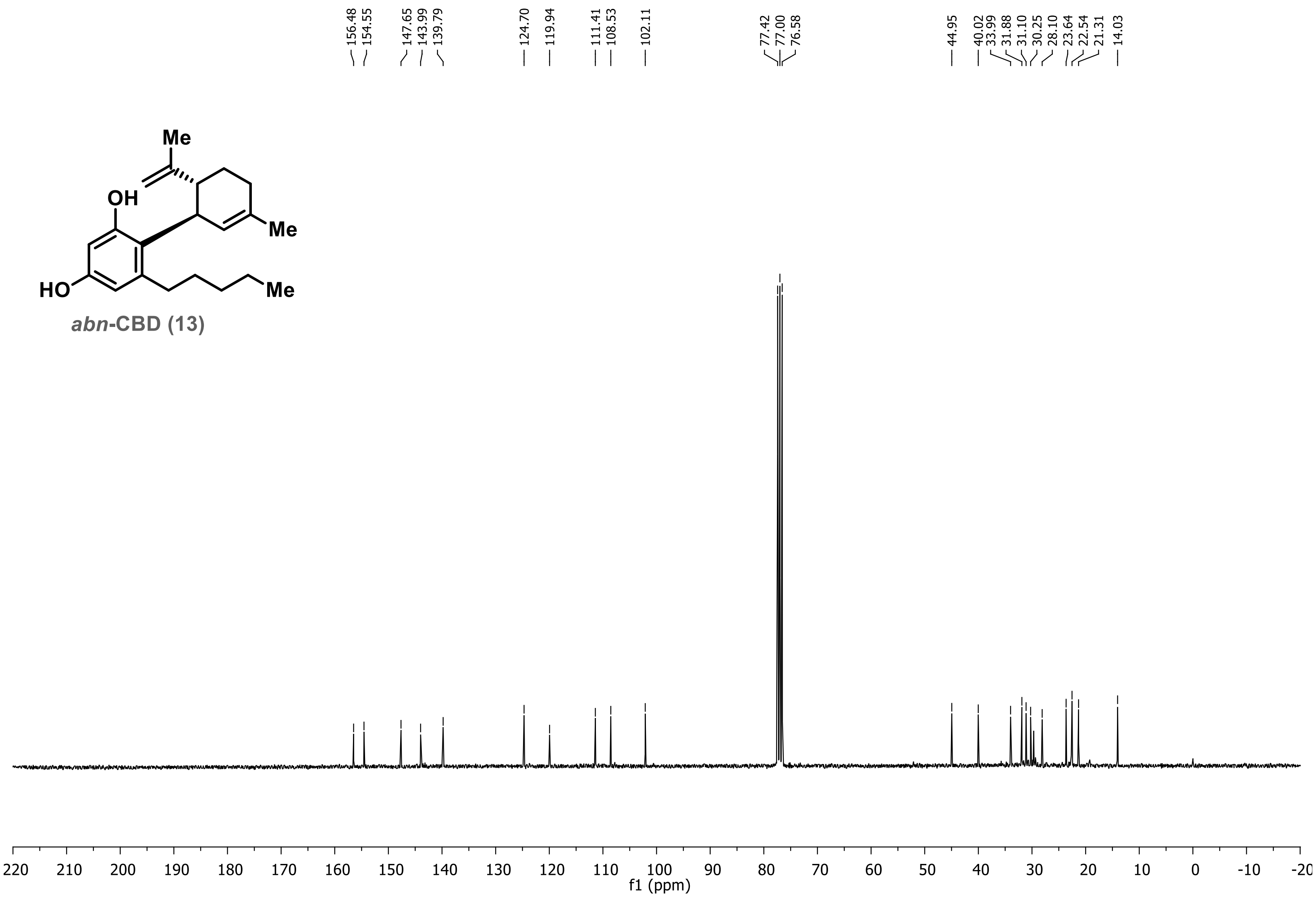

Figure S26. ^{13}C NMR spectrum (75 MHz, $CDCl_3$) of (-)-abnormal cannabidiol (*abn*-CBD, **13**).

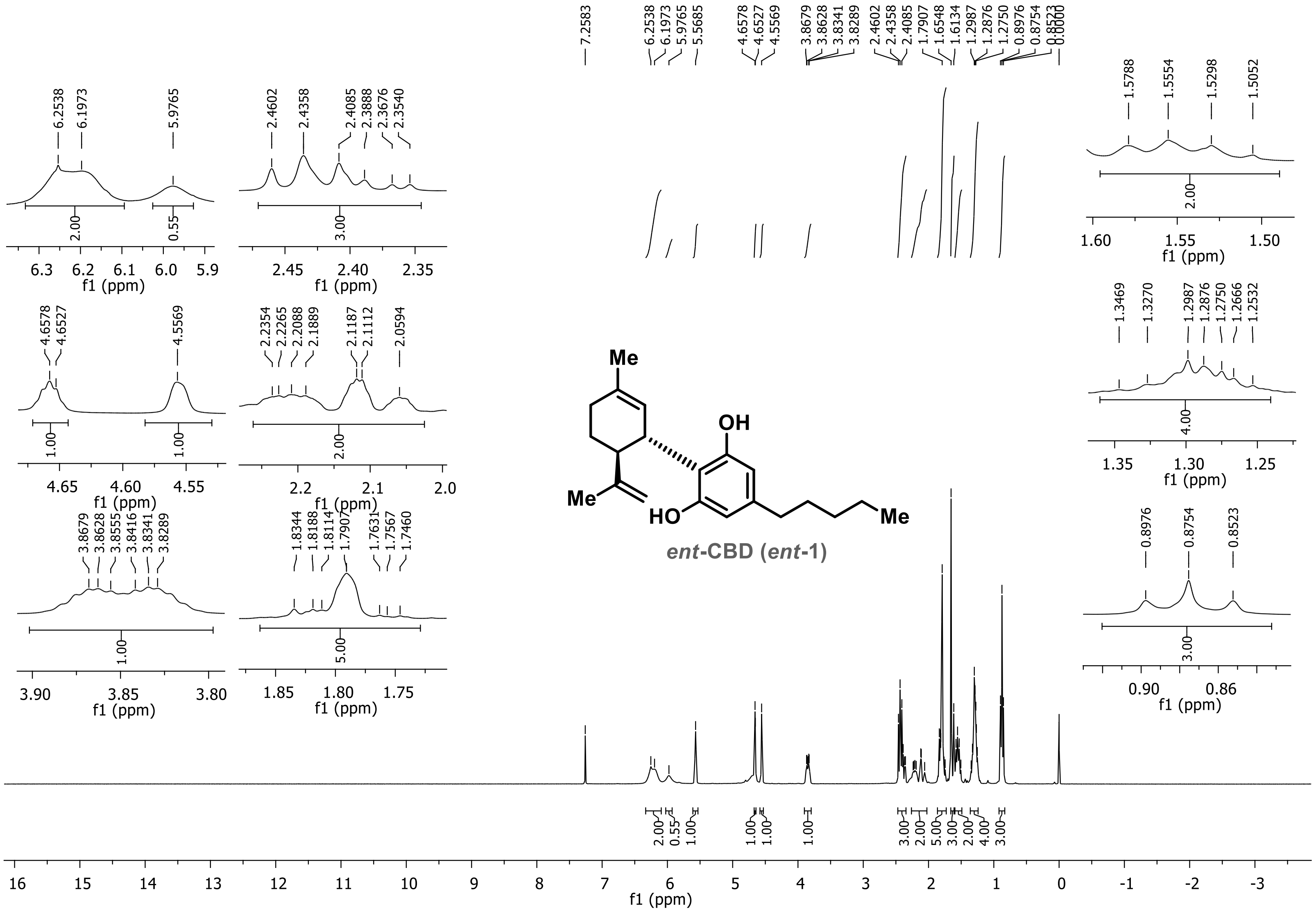

Figure S27. ^{1}H NMR spectrum (300 MHz, $CDCl_3$) of (+)-cannabidiol (*ent*-CBD, *ent*-**1**).

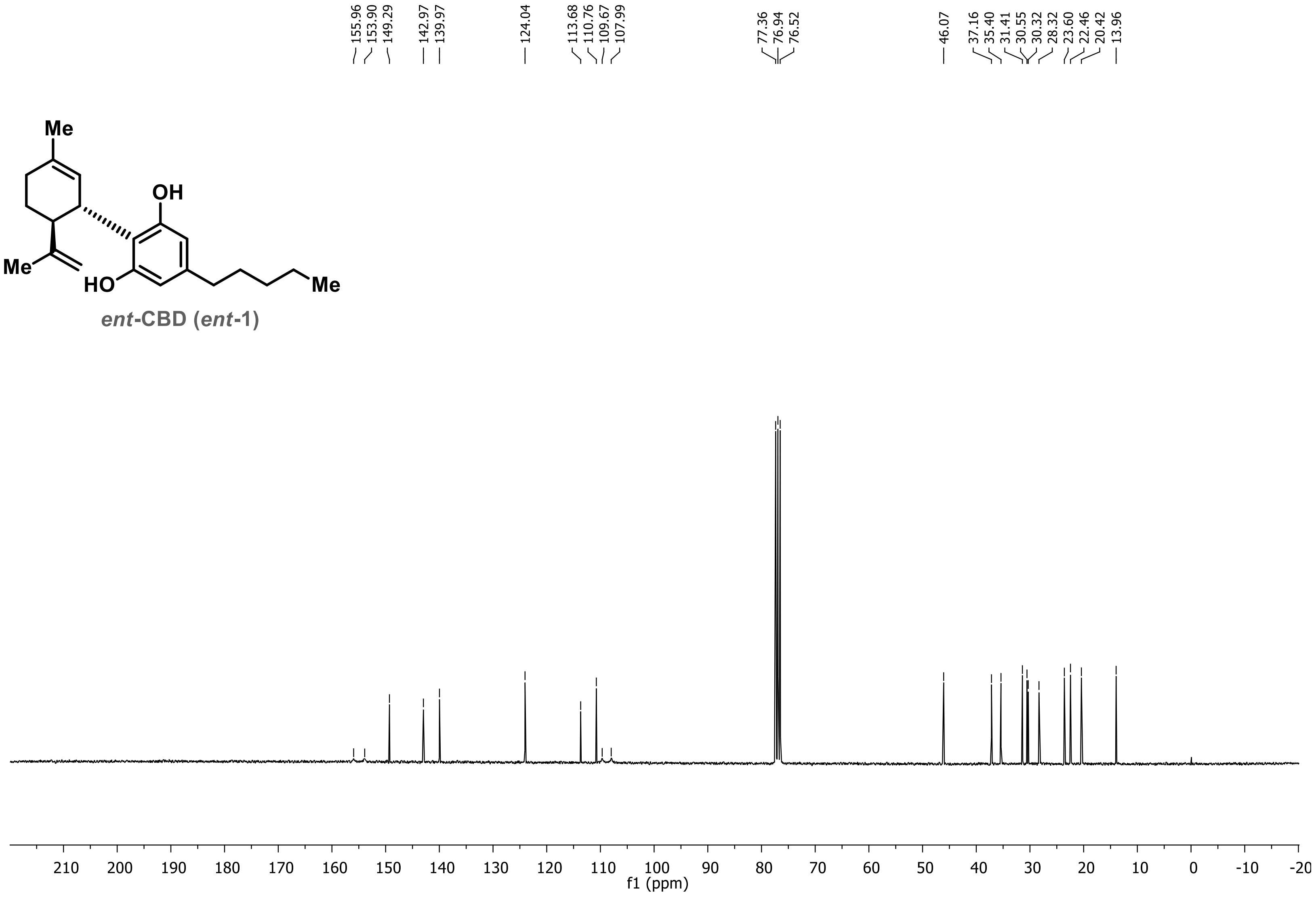

Figure S28. ^{13}C NMR spectrum (75 MHz, $CDCl_3$) of (+)-cannabidiol (*ent*-CBD, *ent*-**1**).

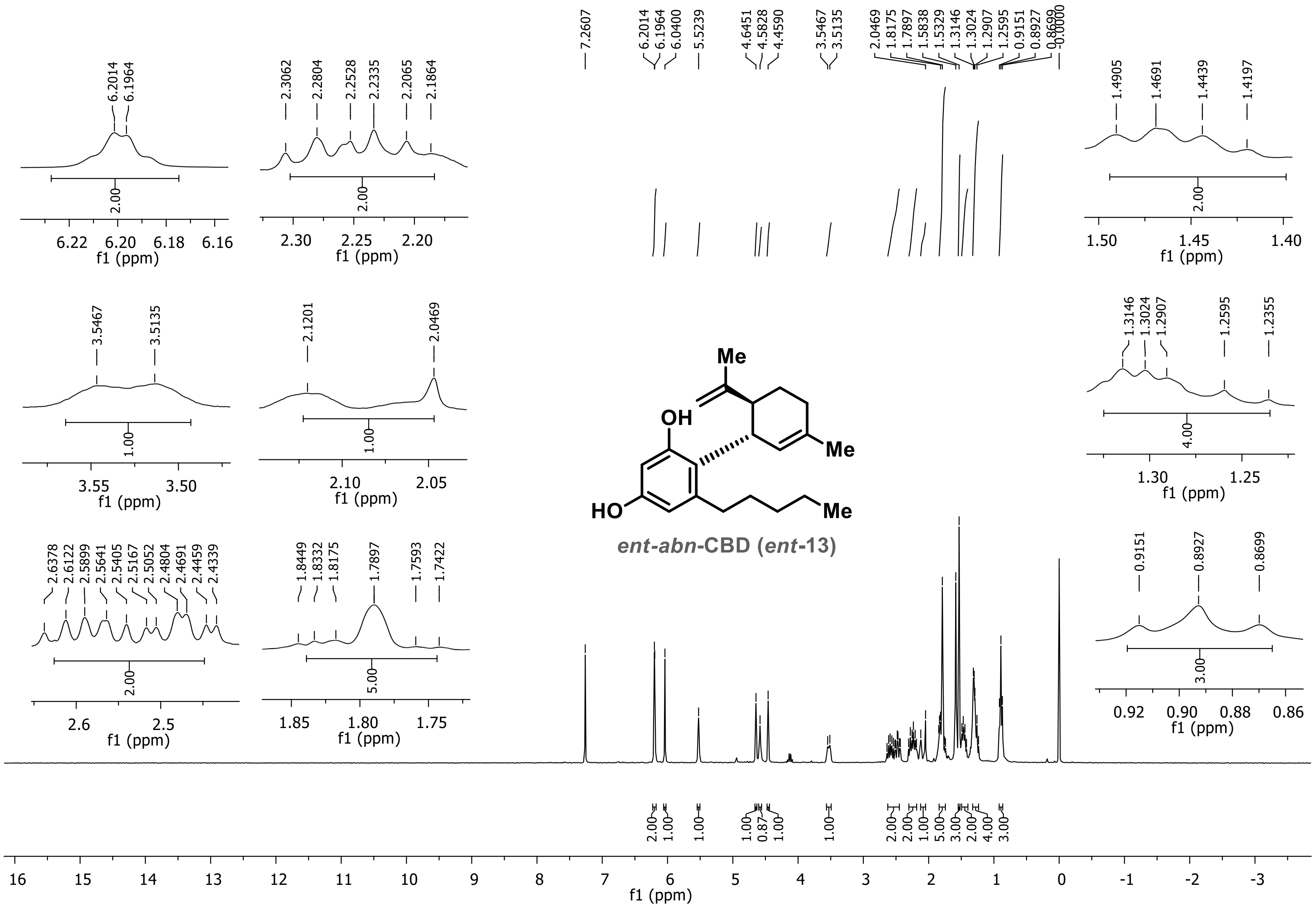

Figure S29. ^{1}H NMR spectrum (300 MHz, $CDCl_3$) of (+)-abnormal cannabidiol (*ent*-*abn*-CBD, *ent*-**13**).

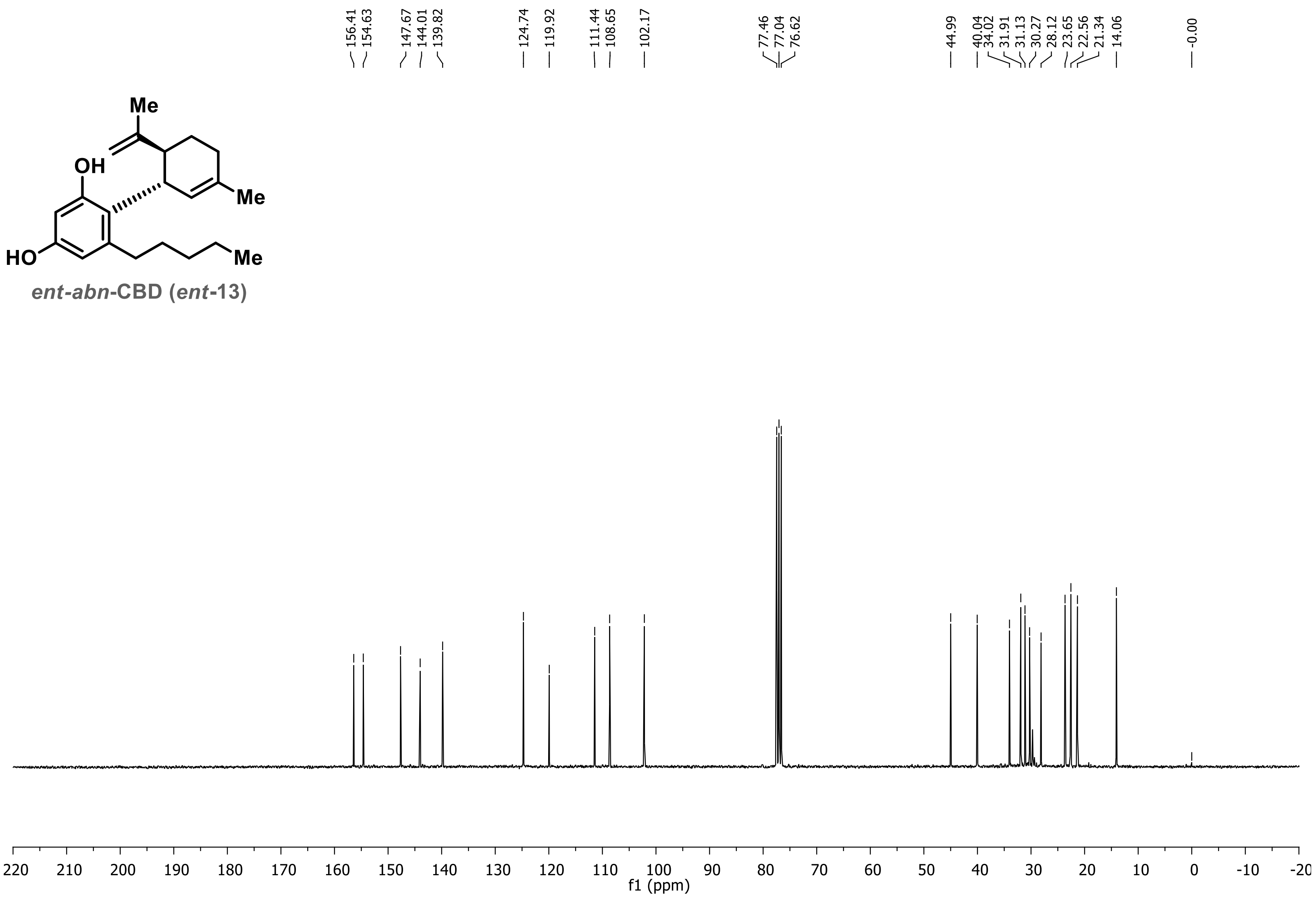

Figure S30. ^{13}C NMR spectrum (75 MHz, $CDCl_3$) of (+)-abnormal cannabidiol (*ent*-*abn*-CBD, *ent*-**13**).

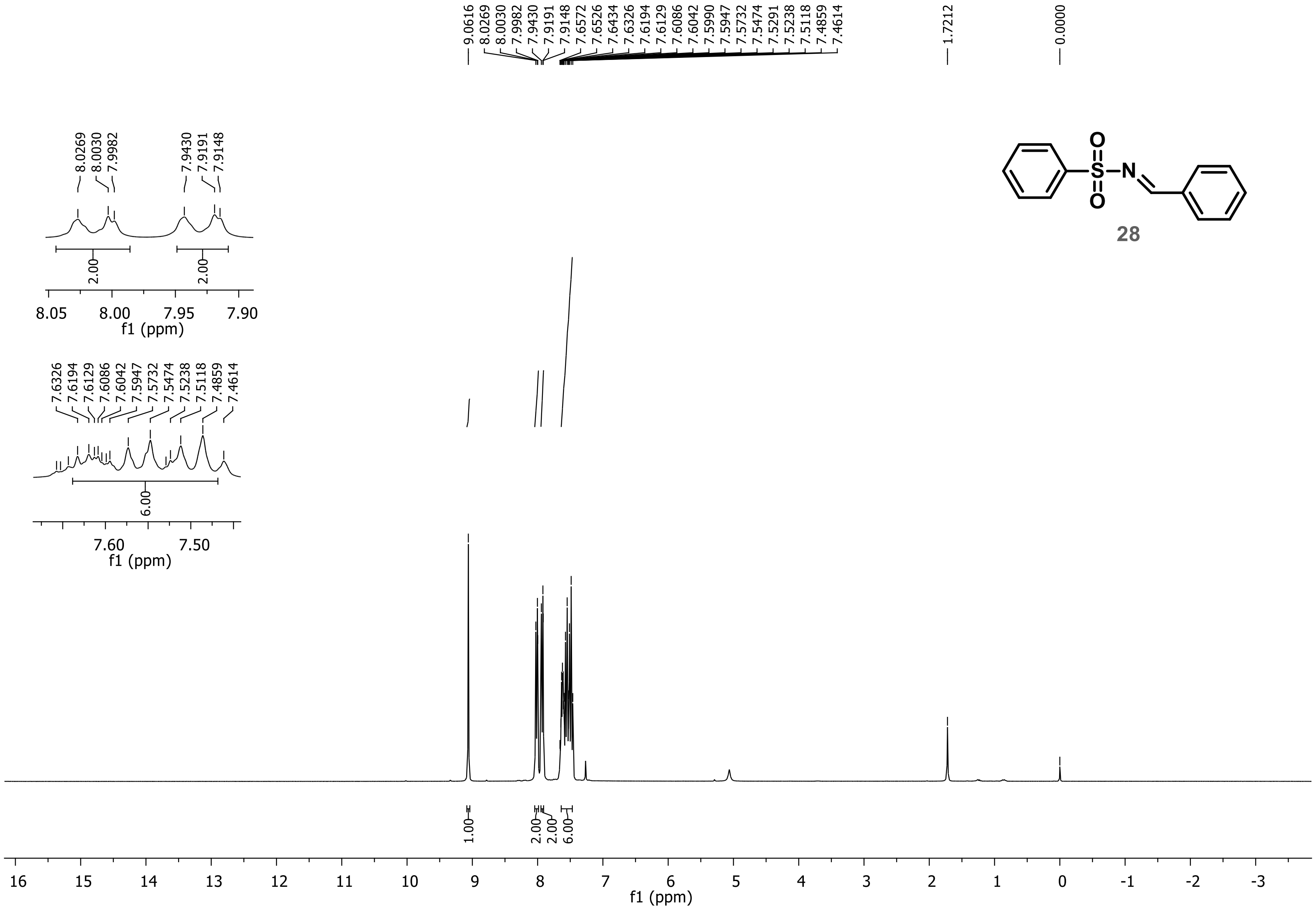

Figure S31. ^{1}H NMR spectrum (300 MHz, $CDCl_3$) of *N*-benzylidenebenzenesulfonamide (**28**).

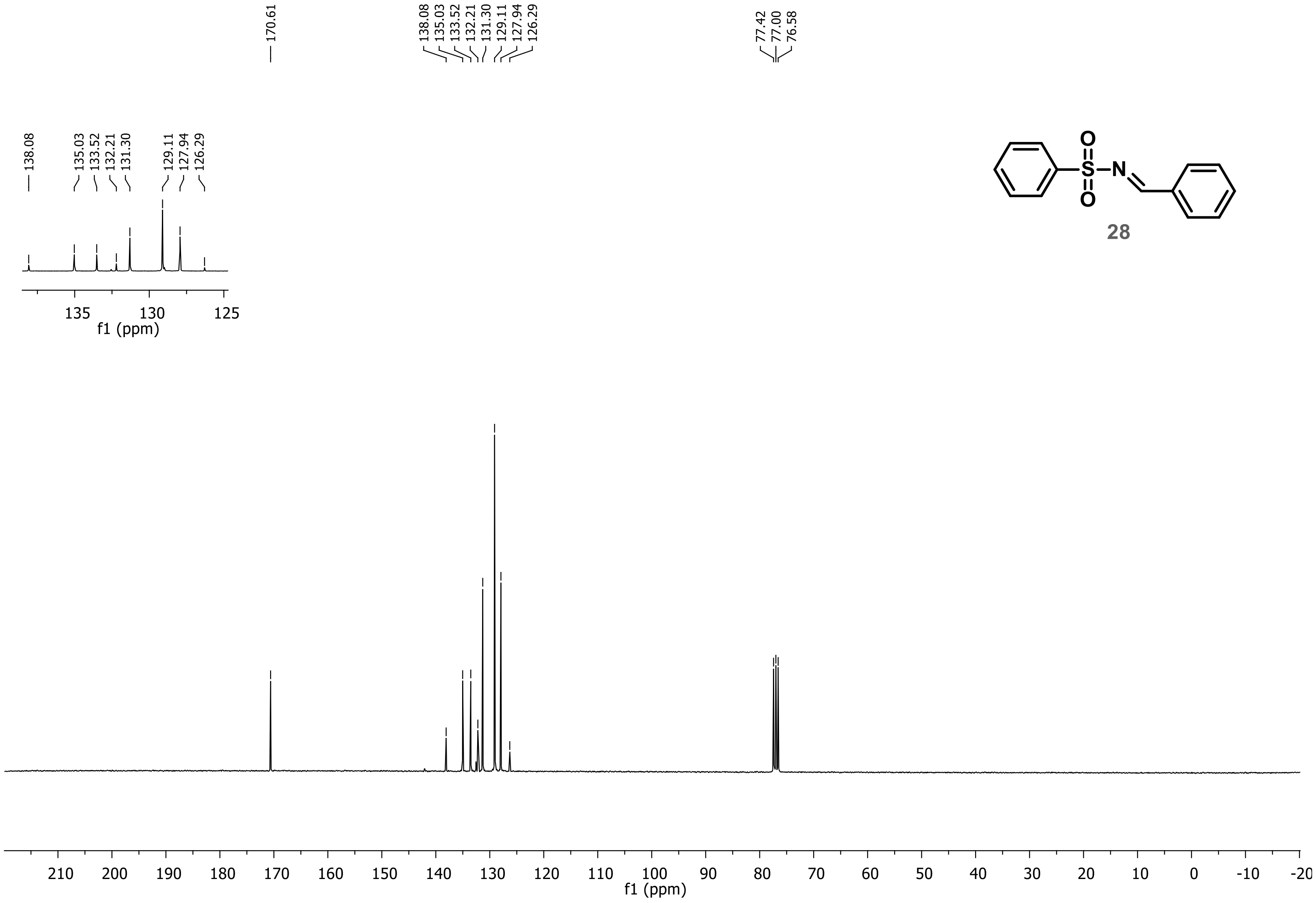

Figure S32. ^{13}C NMR spectrum (75 MHz, $CDCl_3$) of *N*-benzylidenebenzenesulfonamide (**28**).

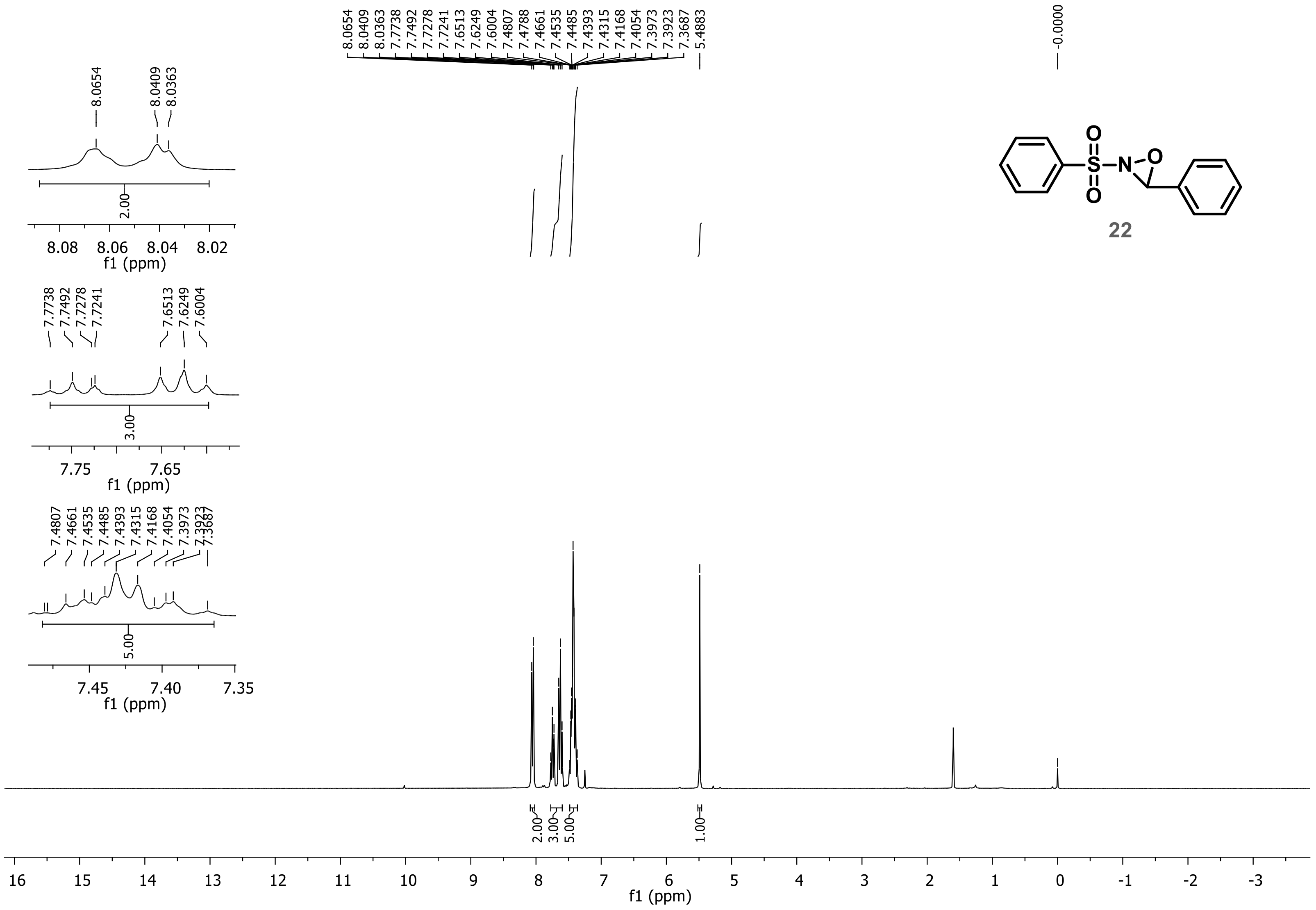

Figure S33. ^{1}H NMR spectrum (300 MHz, $CDCl_3$) of 3-phenyl-2-(phenylsulfonyl)-1,2-oxaziridine (**22**).

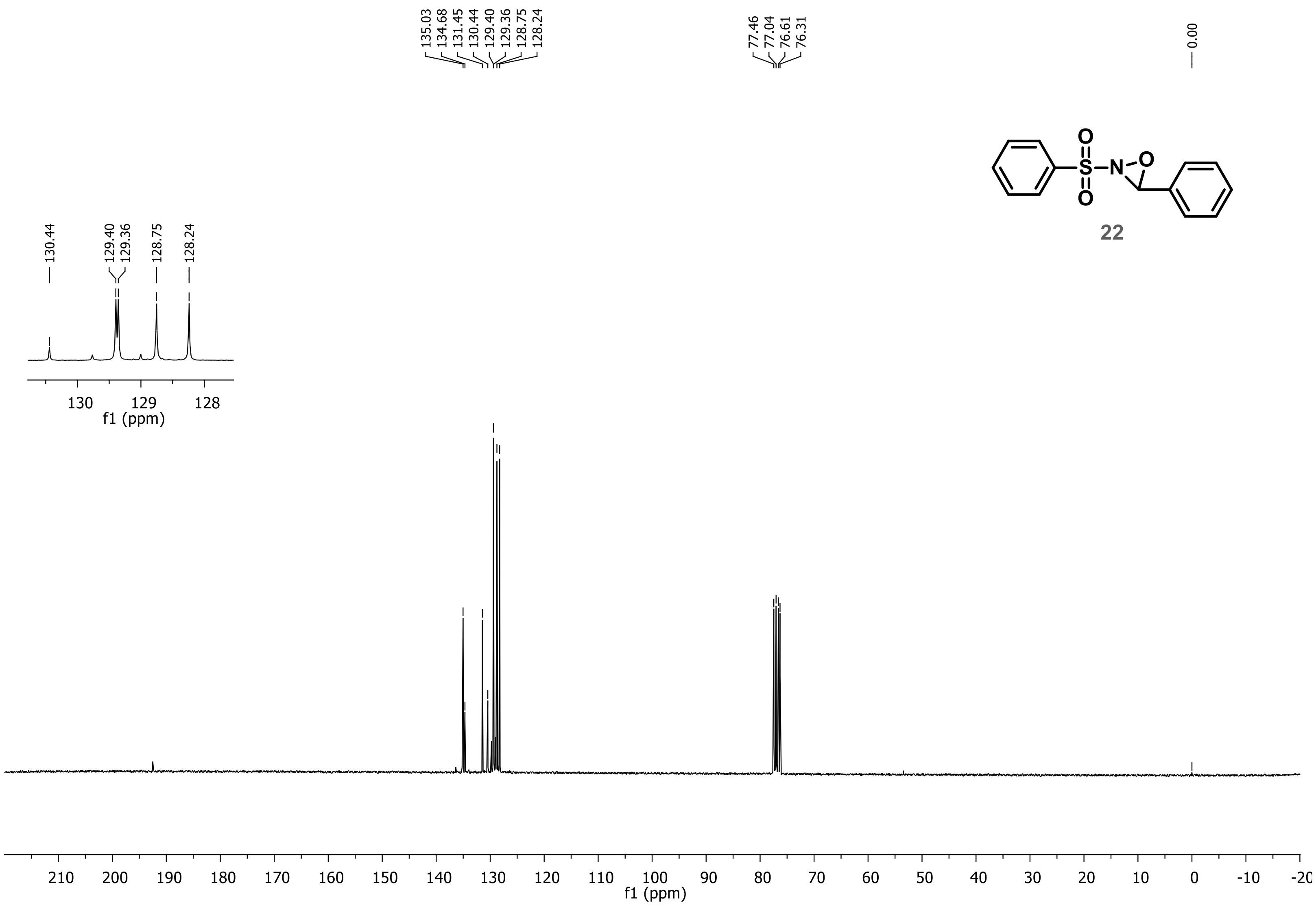

Figure S34. ^{13}C NMR spectrum (75 MHz, $CDCl_3$) of 3-phenyl-2-(phenylsulfonyl)-1,2-oxaziridine (**22**).

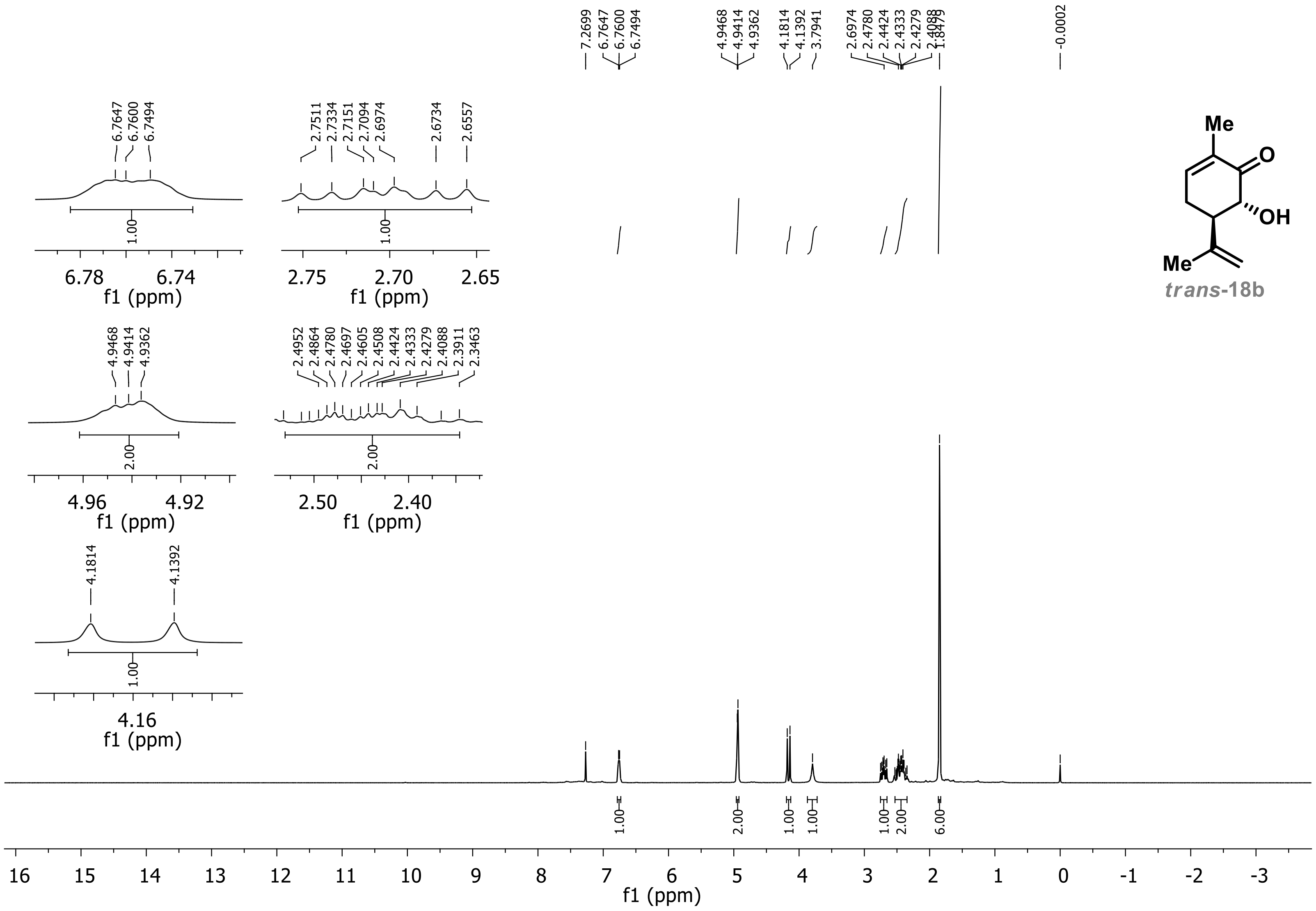

Figure S35. ^{1}H NMR spectrum (300 MHz, $CDCl_3$) of *trans*-(5*R*,6*R*)-6-hydroxy-2-methyl-5-(prop-1-en-2-yl)cyclohex-2-enone (*trans*-**18b**).

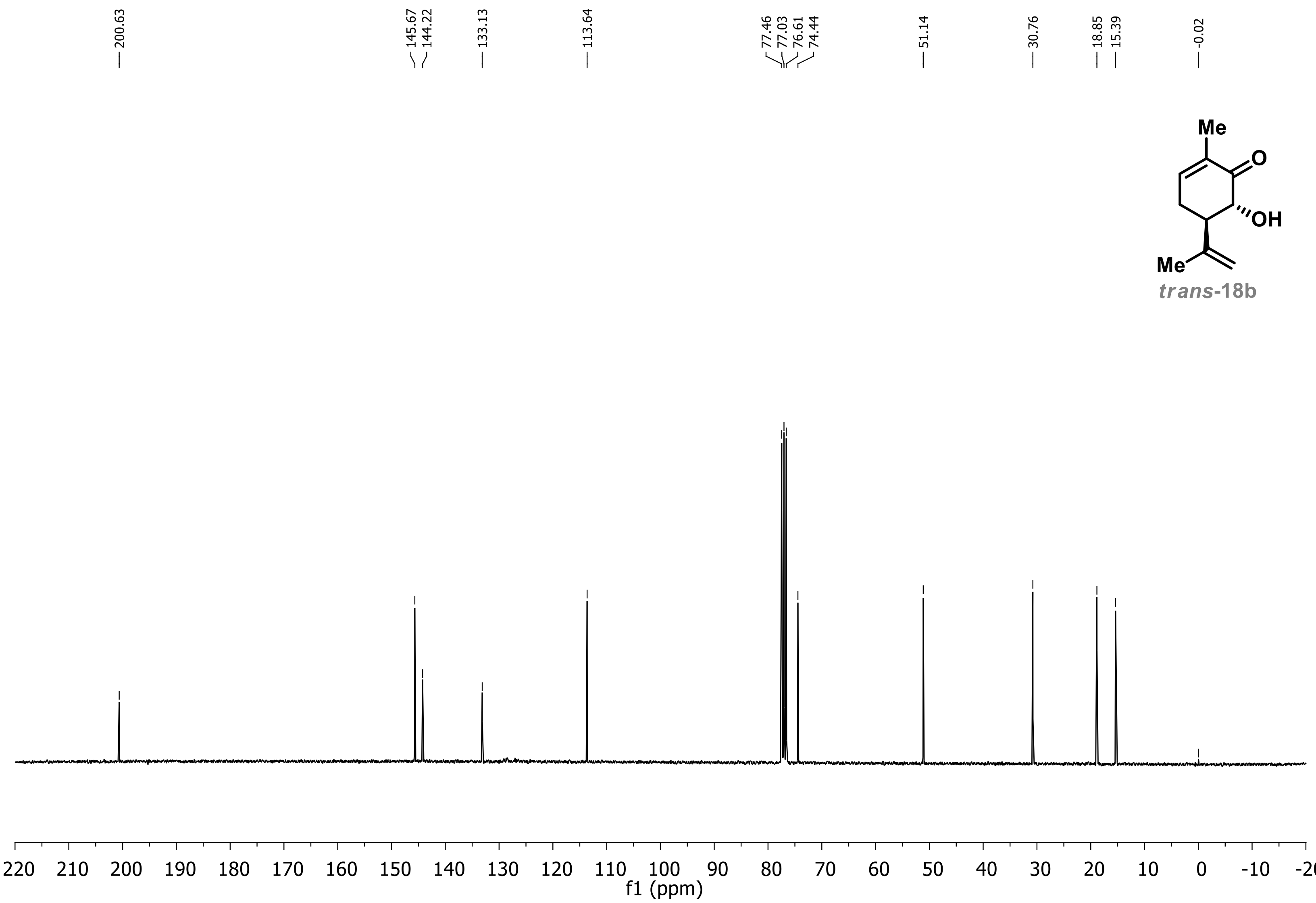

Figure S36. ^{13}C NMR spectrum (75 MHz, $CDCl_3$) of *trans*-(5*R*,6*R*)-6-hydroxy-2-methyl-5-(prop-1-en-2-yl)cyclohex-2-enone (*trans*-**18b**).

Determination of ^{1}H NMR yields

For each entry in **Table S2**, the solvent was removed after work-up. The total mass (65.1-73.3 mg) for each entry was measured. A sample (2.0-4.8 mg) was then added to an NMR tube (5 mm) containing 1,3-dinitrobenzene (2.9-5.2 mg), which was used as an internal standard. The NMR yields of compounds **26** and **27** were determined using the following equations and the values given in **Table S4**:[78]

$$\frac{I_1}{N_1} = \frac{I_2}{N_2} \quad \rightarrow \quad \frac{1}{N_1} = \frac{I_2}{N_2} \quad \rightarrow \quad N_2 = N_1 I_2$$

$$Yield\ (\%) = \frac{m_t}{m_s} \times \frac{N_2}{N_3} \times 100 \quad \rightarrow \quad Yield\ (\%) = \frac{m_t}{m_s} \times \frac{N_1 I_2}{0.3} \times 100$$

where I_1 and I_2 are the NMR integrals of the 1,3-dinitrobenzene (set to 1.00) and product (compound **26** or **27**) signals, respectively; N_1, N_2, and N_3 are the number of moles of 1,3-dinitrobenzene, product (compound **26** or **27**) (unknown), and cyclohex-2-enol (**24**) (0.3 mmol), respectively, and m_t and m_s are the total and sample masses, respectively.

Table S4. Data for determination of ^{1}H NMR yields (see **Table S2**).

Entry	Catalyst (mol%)	Total mass (mg)	Sample mass (mg)	Internal standard mass (mg)[a]	N_1 (mmol)	I_2 for compound **26**[b]	I_2 for compound **27**[c]
1	TMSOTf (20)	69.1	2.3	3.5	0.02082	0.20	0.18
2	AgOTf (20)	73.2	2.4	3.7	0.02201	0.17	0.15
3	$Cu(OTf)_2$ (20)	69.9	4.8	4.3	0.02558	0.06	0.06
4	$Sc(OTf)_3$ (20)	70.4	2.8	4.0	0.02379	0	0
5	$AlCl_3$ (20)	69.3	3.5	2.9	0.01725	0.12	0.12
6	$FeCl_3.6\ H_2O$ (20)	70.9	2.6	3.6	0.02141	0.16	0.15
7	$SnCl_4$ (20)	68.0	3.6	3.5	0.02082	0.18	0.15
8	MsOH (20)	72.4	3.2	5.2	0.03093	0.18	0.14
9	$TsOH.H_2O$ (20)	71.5	2.6	4.5	0.02677	0	0
10	CSA (20)	71.0	2.0	5.2	0.03093	0.11	0.08
11	CSA (10)	73.3	4.1	4.1	0.02439	0.30	0.26
12	CSA (5)	65.1	2.5	5.2	0.03093	0.12	0.11

[a] Signal used: triplet at 9.09 ppm (1H). [b] Signal used: multiplet at 3.94 ppm (1H). [c] Signal used: multiplet at 3.64 ppm (1H).

Biological assays

Animals. Experiments were conducted using 58 young adult (3-6 months old) and 58 aged (15-18 months old) male C57Bl-6 mice obtained from the Center of Experimental Models for Medicine and Biology (CEDEME - Federal University of São Paulo). Mice were housed in the animal facility of the Institute of Environmental, Chemical, and Pharmaceutical Sciences and maintained at 21-23 °C with a 12-hour light/dark cycle. All experiments were conducted during the light period (between 8:00 am and 3:00 pm). Mice were socially housed with 4-5 littermates per cage and had ad libitum access to food and water. Subjects were randomly assigned to different treatments (N = 8 per group). Every effort was made to minimize suffering, pain, or discomfort to the animals during the experimental protocol. The procedures complied with the Brazilian Law for Scientific Research on Animals (No. 11.794/2008) and the NIH Guidelines for the Care and Use of Nonhuman Animals in Research.

Drugs and administration. The following compounds were used in the study: diazepam at a dose of 1 mg/kg (an agonist of the α subunit of the $GABA_A$ receptor used as a positive control), *ent*-CBD (*ent*-**1**) at doses of 3 mg/kg, 10 mg/kg, and 20 mg/kg, and CBD (**1**) at doses of 3 mg/kg and 20 mg/kg (N = 8 per group/age). Diazepam was obtained from a commercial source. *ent*-CBD and CDB were synthesized as described above. Compounds were dissolved in a mixture of 18 parts 0.9% saline solution, 1 part DMSO, and 1 part Tween 12% for intraperitoneal (IP) injection. The vehicle solution used was a 1:1 mixture of DMSO and Tween 12% solution. All animals received their respective treatments (IP) 30 minutes before a PM-DAT training session. Drugs or vehicle were administered only before the training session, with no further administration before the test session. The doses chosen were based on previous studies with CBD reported in the literature.[38]

Apparatus. The plus-maze discriminative avoidance task (PM-DAT) apparatus was made of wood and positioned 50 cm above the floor. It consists of two enclosed arms (EA), each measuring 28.5 x 7 x 18.5 cm, arranged opposite two open arms (OA), each measuring 28.5 x 7 cm, in an L-shaped configuration. One of the enclosed arms, referred to as the aversive enclosed arm (AEA), contains a speaker that emits an 85 dB noise generated by a tone generator and a 100 W lamp. In contrast, the other enclosed arm, referred to as the non-aversive enclosed arm (NAEA), contains no stimuli (lamp and speaker)[39] (Figure S37).

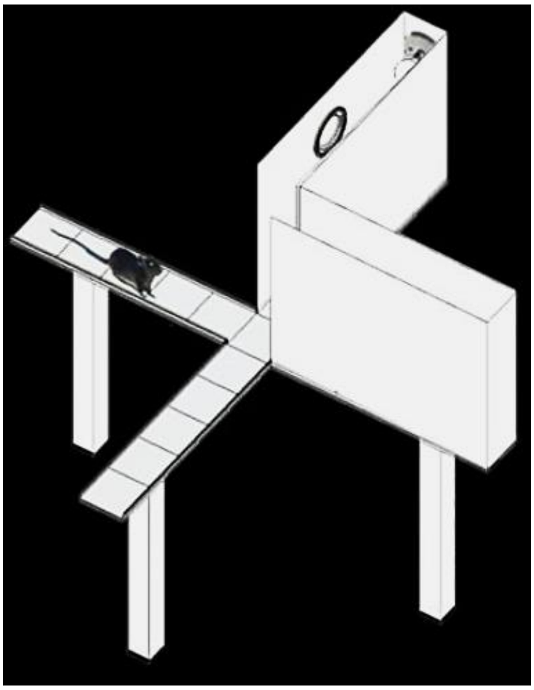

Figure S37. Illustration of the plus-maze discriminative avoidance task (PM-DAT) apparatus.

Handling. To minimize stress, animals were individually habituated to handling by the researcher, with each session lasting 5 minutes for two days. These sessions took place in the animal housing room. On both training and testing days, animals were transferred in their home cages to an anteroom 1-2 hours before the experiment and left undisturbed. After this acclimation period, animals were individually transferred to the experimental room in plastic maintenance cages with bedding identical to their home cages. The experimental sessions were performed during the light phase of the cycle.

Behavioral procedure. The PM-DAT is an adaptation of the well-established elevated plus maze that has been used to assess hippocampal-dependent memory, anxiety-like behavior, and spontaneous motor activity in mice. Animals were individually placed in the PM-DAT apparatus (Figure S37). During the training session (acquisition memory), mice were placed in the center of the apparatus facing an open arm (OA) and their spontaneous locomotor activity in all arms were recorded for 10 minutes. Upon entering the aversive enclosed arm (AEA) with all paws, aversive stimuli (a 100 W light and 85 dB noise) were simultaneously activated and remained on until the animal exited the AEA. Twenty-four hours later, during the test session (evocation memory), the mice were reintroduced to the apparatus and allowed to explore for 3 minutes without any aversive stimuli, only cues proximal and distal

were maintained. Both sessions were recorded with a digital camera mounted on the ceiling, and the light level in the room was controlled throughout. The apparatus was disinfected with 10% ethanol before each new animal was introduced for testing.[39]

Behavioural analysis. Anxiety-like behavior was assessed by calculating the percentage of time spent in open arms (OA) [% time in OA = (time spent in OA / total time spent in all arms) × 100]. Memory performance was assessed by calculating the percentage of time spent in the aversive enclosed arm (AEA) [% time in AEA = (time spent in AEA / total time spent in both enclosed arms) × 100] during the test session, reflecting long-term memory retention.[39] Spontaneous motor activity was evaluated using the number of entries into non-aversive enclosed arm (NAEA), as anxiety can reduce overall motor activity and decrease both the percentage of time spent in and the number of entries into aversive arms (open and enclosed).

Statistical analysis. One-way analysis of variance (ANOVA) was used to assess the effect of treatment on the variables. Post hoc Bonferroni's tests for multiple comparisons were used when necessary at a significance level of 5% or less ($p \leq 0.05$). All data are presented as mean ± SEM. All statistical analyses were performed using GraphPad Prism 9.0 software (GraphPad Inc., San Diego, CA, USA).

References

[51] W. L. F Armarego, D. D. Perrin, *Purification of Laboratory Chemicals*, 4th ed.; Butterworth-Heinemann: Oxford, **1996**.

[52] B. S. Furniss, A. J. Hannaford, P. W. G. Smith, Tatchell, A. R. *Vogel's Textbook of Practical Organic Chemistry*, 5th ed.; Longman Scientific & Technical, **1989**.

[53] S. C. Watson, J. F. Eastham "Colored indicators for simple direct titration of magnesium and lithium reagents", *J. Organomet. Chem.* **1967**, *9*, 165–168.

[54] J. Leonard, B. Lygo, G. Procter, *Advanced Practical Organic Chemistry*, 2nd ed.; CRC Press: Boca Raton, **1998**, 102.

[55] K. Mori, H. Takaishi "Synthesis of Mono- and Sesquiterpenoids, XVI Synthesis of (-)-Pereniporins A and B, Sesquiterpene Antibiotics from a Basidiomycete", *Liebigs Ann. Chem.* **1989**, 939–943.

[56] R. G. Doveston, R. Steendam, S. Jones, R. J. K. Taylor "Total Synthesis of an Oxepine Natural Product, (±)-Janoxepin" *Org. Lett.* **2012**, *14*, 1122–1125.

[57] A. B. Smith, T. Bosanac, K. Basu "Evolution of the Total Synthesis of (−)-Okilactomycin Exploiting a Tandem Oxy-Cope Rearrangement/Oxidation, a Petasis−Ferrier Union/Rearrangement, and Ring-Closing Metathesis", *J. Am. Chem. Soc.* **2009**, *131*, 2348–2358.

[58] F. Khachik, A. N.Chang "Synthesis of tetrahydrolipstatin", *J. Org. Chem.* **2009**, *74*, 3875–3885.

[59] T. L. Ho, C. Y. Su "Total Synthesis of (±)-Nudenoic Acid", *J. Org. Chem.* **2000**, *65*, 3566–3568.

[60] J. A. R. Salvador, M. L. Sá e Melo, A. S. Campos Neves "Copper-catalysed allylic oxidation of Δ^5-steroids by t-butyl hydroperoxide", *Tetrahedron Lett.* **1997**, *38*, 119–122.

[61] J. Q. Yu, E. J. Corey "Diverse Pathways for the Palladium(II)-Mediated Oxidation of Olefins by tert-Butylhydroperoxide", *Org. Lett.* **2002**, *4*, 2727–2730.

[62] T. K. M. Shing, Yeung, P. L. Su "Mild Manganese(III) Acetate Catalyzed Allylic Oxidation: Application to Simple and Complex Alkenes", *Org. Lett*. **2006**, *8*, 3149–3151.

[63] O. V. Ardashov, A. V. Pavlova, I. V. Il'ina, E. A. Morozova, D. V. Korchagina, E. V. Karpova, K. P. Volcho, T. G. Tolstikova, N. F. Salakhutdinov "Highly Potent Activity of (1*R*,2*R*,6*S*)-3-Methyl-6-(prop-1-en-2-yl)cyclohex-3-ene-1,2-diol in Animal Models of Parkinson's Disease", *J. Med. Chem.* **2011**, *54*, 3866–3874.

[64] L. Friedman, R. L. Litle, W. R. Reichle "p-Toluenesulfonylhydrazide", *Org. Synth.* **1960**, *40*, 93.

[65] J. Safaei-Ghomi "An Efficient Synthesis of Sulfonylhydrazides and Sulfonylsemicarbazides by Utilizing Alumina as a Catalyst", *J. Chin. Chem. Soc.*, **2007**, *54*, 1561–1563.

[66] A. N. Cheallaigh, D. J. Mansell, H. S. Toogood, S. Tait, A. Lygidakis, N. S. Scrutton, J. M. Gardiner “Chemoenzymatic Synthesis of the Intermediates in the Peppermint Monoterpenoid Biosynthetic Pathway”, *J. Nat. Prod.* **2018**, *81*, 1546–1552.

[67] A. Loupy, J. Seyden-Penne “The influence of lithium complexing agents on the regioselectivity of reductions of substituted 2-cyclohexenones by $LiAlH_4$ and $LiBH_4$”, T*etrahedron* **1980**, *36*, 1937–1942.

[68] L. Dialer, D. Petrovic, U. Weigl, Process for the production of cannabidiol and delta-9-tetrahydrocannabinol WO 2017/011210 A1, **2017**.

[69] T. M. Waugh, J. Masters, A. E. Aliev, C. M. Marson “Monocyclic Quinone Structure-Activity Patterns: Synthesis of Catalytic Inhibitors of Topoisomerase II with Potent Antiproliferative Activity”, *ChemMedChem* **2020**, *15*, 114–124.

[70] S. Beak, Y. Kim “A Simple and Convenient Method for the Synthesis of Olivetols”, *Bull. Korean Chem. Soc.* **1993**, *14*, 272–274.

[71] R. K. Razdan, H. C. Dalzell, G. R. Handrick “Hashish. X. Simple one-step synthesis of (-)-.DELTA.1-tetrahydrocannabinol (THC) from p-mentha-2,8-dien-1-ol and olivetol”, *J. Am. Chem. Soc.* **1974**, *96*, 5860–5865.

[72] S. J. Byard, S. A. Carr, J. F. DeFaria, C. N. Filer, J. M. Herbert, T. J. Forcada “The preparation of [*pentane*-5,5,5-^{3}H3]-abnormal-cannabidiol”, *J. Label Compd. Radiopharm* **2011**, *54*, 180–184.

[73] F. A. Davis, W. A. R. Slegeir, S. Evans, A. Schwartz, D. L. Goff, R. Palmer “Chemistry of the sulfur-nitrogen bond. VI. Convenient one-step synthesis of sulfenimines (S-aryl thiooximes)”, *J. Org. Chem.* **1973**, *38*, 2809–2813.

[74] H. Sharghi, M. Hosseini-Sarvari, S. A. Ebrahimpourmoghaddam “A novel method for the synthesis of N-sulfonyl aldimines using $AlCl_3$ under solvent-free conditions (SFC)”, *Arkivo*c **2007**, 255–264.

[75] J. L. G. Ruano, J. Aleman, C. Fajardo, A. Parra “A New General Method for the Preparation of N-Sulfonyloxaziridines”, *Org. Lett.* **2005**, *7*, 5493–5496.

[76] F. A. Davis, U. K. Nadir, E. W. J. Kluger “2-Arylsulphonyl-3-phenyloxaziridines: a new class of stable oxaziridine derivatives”, *Chem. Soc. Chem. Comm*. **1977**, 25–26.

[77] S. Issa, H. S. Toogood, L. O. Johannissen, J. Raftery, N. S. Scrutton, J. M. Gardiner “C3 and C6 Modification-Specific OYE Biotransformations of Synthetic Carvones and Sequential BVMO Chemoenzymatic Synthesis of Chiral Caprolactones”, *Chem. Eur. J.* **2019**, *25*, 2983–2988.

[78] Y. Qu, C. Tsuneidhi, H. Tateno, Y. Matsumura, M. Atobe “Green synthesis of α-amino acids by electrochemical carboxylation of imines in a flow microreactor”, *React. Chem. Eng*. **2017**, *2*, 871–875.
